# Subclinical cardiovascular disease and utility of coronary artery calcium score

**DOI:** 10.1016/j.ijcha.2021.100909

**Published:** 2021-11-17

**Authors:** Cihan Durmuş Saydam

**Affiliations:** Muhsin Yazıcıoğlu Cad., No:10 Üst Kaynarca Pendik, Istanbul 34899, Turkey

**Keywords:** FRS, SCORE, SCVD, CACS, CVE, ASCVD

## Abstract

ASCVD are the leading causes of mortality and morbidity among Globe. Evaluation of patients’ comprehensive and personalized risk provides risk management strategies and preventive interventions to achieve gain for patients. Framingham Risk Score (FRS) and Systemic Coronary Risk Evaluation Score (SCORE) are two well studied risk scoring models, however, can miss some (20–35%) of future cardiovascular events. To obtain more accurate risk assessment recalibrating risk models through utilizing novel risk markers have been studied in last 3 decades and both ESC and AHA recommends assessing Family History, hs-CRP, CACS, ABI, and CIMT. Subclinical Cardiovascular Disease (SCVD) has been conceptually developed for investigating gradually progressing asymptomatic development of atherosclerosis and among these novel risk markers it has been well established by literature that CACS having highest improvement in risk assessment. This review study mainly selectively discussing studies with CACS measurement. A CACS = 0 can down-stratify risk of patients otherwise treated or treatment eligible before test and can reduce unnecessary interventions and cost, whereas CACS ≥ 100 is equivalent to statin treatment threshold of ≥ 7.5% risk level otherwise statin ineligible before test. Since inflammation, insulin resistance, oxidative stress, dyslipidemia and ongoing endothelial damage due to hypertension could lead to CAC, ASCVD linked with comorbidities. Recent cohort studies have shown a CACS 100–300 as a sign of increased cancer risk. Physical activity, dietary factors, cigarette use, alcohol consumption, metabolic health, family history of CHD, aging, exposures of neighborhood environment and non-cardiovascular comorbidities can determine CACs changes.

## 1 Introduction

Cardiovascular diseases and events due to atherosclorosis are leading causes of mortality and morbidity among Globe. Atherosclerosis develops gradually without giving a clinical event and is affected from how do we live as in life style factors. Evaluation of patients’ comprehensive and personalized risk, provides risk management strategies and preventive interventions to achieve gain for patients. Prevention should be delivered by promoting healthy lifestyle behaviour [2]. Risk assessment should be done in lifetime continuum because of dynamically changing Cardiovascular risk level and matching preventive interventions with individual’s absolute risk [1], [18]; moreover, as a result of population level primordial, primary and secondary prevention strategies [27], [28], two thirds to three fourths of reduction in CVD mortality rates in several countries during previous decades had been explained with population shifts in risk factor levels [18]).

## 2 Risk assessment models

There are some risk assessment systems targeting apparently healthy people to estimate total cardiovascular risk especially for those at high risk, for instance Systemic Coronary Risk Evaluation (SCORE) score and Framingham Risk Score (FRS) which are endorsed by European and American guidelines respectively.

## 3 Definitions of SCORE and FRS, and risk assessments of CVD and CHD

SCORE risk system estimates individuals’ 10 year risk of mortality due to CVD and utilizes variables of Sex, Age, Total Cholesterol, Total Cholesterol/HDL-C ratio, SBP and smoking history [2]. European Guidelines on CVD Prevention don’t recommend systematic CV risk assessment in men < 40 of age and women < 50 years of age unless have documented previous history of CVD, DM, kidney disease or end-organ damage at 3C class of recommendation and level of evidence [2].

Framingham risk system estimates 10 year risk of developing first ASCVD event and provides gender and ethnicity specific results in age range of 40 to 79. Variable of this risk assessment system includes age, total cholesterol, high density lipoprotein cholesterol, systolic blood pressure, diabetes mellitus and current smoking status [1].

Clinical guidelines impose thresholds on risk factor levels to guide decision making and the association of risk factor levels with CVD risk is continuous and graded across all levels [18]. SCORE system stratify patients according to their calculated SCORE risk as low risk for patients’ SCORE < 1%, low-to-moderate risk when SCORE is below 5%, high risk in a range from 5% to below 10% and very high risk whom SCORE is equal>10% threshold. AHA stratify patients on 3 categories which are low risk level for FRS < 10%, intermediate risk for FRS from 10% to 19% and high risk for FRS ≥ 20%. However, These scoring models can miss some (20%-35%) of future cardiovascular events [23], [38], [39].

Beyond those conventional risk models, as a well-established concepts [1], both European and American guidelines recommend recalibration of risk assessment with additional novel risk parameters and reclassification especially for patients at risk stratification thresholds i.e. on intermediate risk [2], [5], [6], [29]. Those novel markers should add significant incremental prognostic information to a model with traditional risk factors [3]. Estimated probabilities at decisional thresholds may require additional nonconventional risk factors and some imaging techniques may be considered as risk modifiers to improve accuracy of risk prediction [1], [2]. Those well studied imaging methods are coronary artery calcium score, atherosclerotic plaque detection, ABI and carotid ultrasound for intima media thickness measurements [2], [5], [10], [20]. Noninvasive techniques continue to evolve and improve detecting subclinical disease, as well as possibly intervening and preventing clinical events [21], [22]. More accurate risk evaluation should be done in correlation with total cardiovascular risk as including subclinical cardiovascular changes in addition to conventional risk assessment [4], [19], [44]. According to both AHA and ESC, when risk based decision is uncertain, assessing family history of premature CVD (positive sign if presents with first degree relatives of Male < 55 years of age or Female < 65 years of age) , hs-CRP (with threshold of 2 mg/dl), CAC score (with cut-off of 300 Agatston units or 75 percentile for age, sex, and ethnicity), and ABI (risk sign if its < 0.9) should be considered for inform decision making but routine measurement of CIMT is not recommended in clinical practice for risk assessment for a first ASCVD event[1], [2]. Furthermore, for the same setting American Society of Echocardioraphy Consensus reports plaque detection, that is focal wall thickening in at least 50% or greater of surrounding vessel wall or focal region protruding into lumen in size of IMT measurement with threshold of 1.5 mm, as a considerable risk modifier in CV risk prediction [2], [33], [34]. Furthermore, arterial stiffness [7], that is commonly measured using either Arterial Tonometry (aortic pulse wave velocity-aPWV [35]) or Flow Mediated Dilatation-FMD (arterial augmentation index-AAI) [61], may serve as a useful biomarker improve CV risk assessment for patients at around decisional thresholds, however assessing vascular function is not recommended in population or asymptomatic adults for risk modification [2], [19], [24].

## 4 Subclinical cardiovascular disease

Subclinical disease can be defined as in entity gradually progressing to clinical presentation with symptoms and subclinical disease is an earlier, more medically treatable state than clinical disease [3], [15], [16], [17], [32]. Risk factors may results in the development of subclinical atherosclerosis which precedes the occurence of clinical events by years to decades [14], [18]. Thus a long latent period of CVD during which it remains subclinical, making it an ideal target for screening tests [9], [23]. Although, Polonsky et al. suggest measures of CVH involving pathways other than atherosclerosis related CAC and CIMT, and measures of cardiac stress such as LVMI [8].

According to AHA, construct of ideal cardiovascular health suggest simultaneous presence of 4 favorable health behaviors, 4 favorable health factors and absence of clinical CVD; moreover, since smoking status appears in both list, AHA defined a total of 7 health behaviors and factors [18]. In a community based HeartSCORE cohort, association between CVH and CVD was observed in inverse relation [8], [25] and higher level of ideal CVH was associated with decreased burden of subclinical CVD, lower levels of CAC, lower CIMT and improved endothelial function without difference when adjusted with age, sex, race, income, education and marital status [12]. Similarly Polonsky et al. observed significant and substantial associations of high and moderate levels of CVH with lower risk for CVD remained with partial attenuation after adjustment for measures of subclinical CVD, which are CAC, CIMT and LVMI; while they also showed increasing significant hazard ratios for relation between CVH and CHD after adjusting with each of those subclinical CVD measures [8].

## 5 Measurement of CACS

Coronary artery calcium is quantified through multi slice(detector) non-contrast cardiac CT and any potential focus of CAC is defined as 3 or more adjacent contagious pixels(voxels) in area size of 1.0 or 1.87 mm2, with a CT number > 130 HU in a field of view with total lenghth of 35 cm [31], [36], [37]. Current generation of multi detector CT can acquire up to 128–320 sections of heart simultaneously with ECG gating in either prospective or retrospective mode and Coronary calcificattion is determined in the prospective axial mode at a predetermined offset from the ECG-detected R-wave within temporal resolution proportional to gantry speed [39]. Those acquired images are contagious slices without any interslice gaps in 3 mm size [30]. Agatston scoring method bases on weighted sum of any hyperattenuated lesion, multiplying area of calcium by it’s weighting factor depending on maximum plaque attenuation [30], [38], [39]. Numbers of density weighting factors 1 to 4 are defined arbitrarily with intervals of CT number above the threshold of 130 HU according to peak CT number of each lesions as 130 HU to<200 HU, 200 HU to<300 HU, 300 HU to<400 HU and beyond 400 HU, respectively [40]. Similarly CAC density score is the average density factor of all CAC areas by dividing CAC Agatston score by the total CAC area, ranging from 1 to 4 [48]. Identical to Agatston method ,Volume scoring method uses same scanning parameters, though it doesn’t rely on lesion density by the number of density weighting factor and it focuses on estimating true volume of calcified plaques, in doing so for each calcified lesion sufficing attenuation and area thresholds, assigned numerical values of each voxels by isotropic interpolation are multiplied by their volume, and then the total volume score is summed for all individual lesions regardless of their regional distribution [41], [42]. However, this method has sensitivity to partial volume especially in small plaques with high attenuation and variability of the test due to position of plaque in acquired axial slice; moreover calcium volume score can overestimate calcium content [38], [40]. Unlike Agatston score and volume score, mass score measures an absolute mass of mineral directly in milligrams and provides more accurate, less variable and more reproducible quantification of coronary calcium than Agatston Score and Volume Score, respectively [43], [45]. For calculating mass score calibration phantom by calcified cylinder with known calcium concentration, which have been placed beneath the thorax of the subject before examination, is used during image acquisition and calibration factor is determined through dividing cylinder’s known concentration with subtracting mean CT number of water in HU unit from mean CT number of cylinder in HU unit [43]. Product of calibration factor, volume of lesion and mean CT number of each lesion in HU unit gives the mass score for each lesion, and then total mass score is calculated as the sum of the mass of all individual lesions without accounting their regional distribution [41]. All 3 scores are tightly correlated and small differences in reproducibility between these scores may not be clinically significant [43]. Agatston score remains gold standard CAC scoring method [41] as most extensively studied and rightly most widely accepted method [43].

## 6 Distribution and prevalence of CAC on demographic features

McClelland et al. [52], a MESA study with follow-up of 6814 patients, measured higher non-zero CAC score among male patients a 60% compare to female participants at 38%. Detrano et al [53], a MESA follow-up study of 6722 participants along with a median time of 3.9 years, prevalence of CAC was measured for 4 racial groups in both genders as follows: Whites (M:70.4%, F:44.7%), Black (M:52%, F:37%), Hispanic (M:56.6%, F:34.8%) and Chinese (M:59.2%, F:41.9%). Same study showed that rate of detectable calcium in relation to age increases more steeply at concave down among males compare to females at concave up; moreover, gender difference was most apparent for whites, particularly in the middle age range. At older ages, above 70 years of age for men and 75 years of age for women, Chinese had lowest CAC score, while at younger ages lowest values of CAC score were measured for Hispanic women and Black men. Whites had been observed consistently with higher CAC score at any age group than the other 3 racial groups. A CARDIA study [54], 5115 participants aged 18 to 30, at the year of 25 in follow-up measured non-zero CAC score at prevalence of 41.8%.

## 7 Predictive, recalibrating and risk stratifying value of CAC

Net Reclassification Improvement is defined as a summation of two subtraction operation, where probability of being correctly reclassified category or event is subtracted by probability of being incorrectly reclassified category or event, for high risk and low risk [50].

Yeboah et al. [55], a MESA report of 1330 patients with intermediate Framingham Risk Score from 5% to 20% and without diabetes mellitus in median follow-up of 7.6 years, showed that by addition of CAC to FRS, 25.5% of events group had been classified to high risk and 40.4% of the non-event group had been classified to low risk, and therefore NRI had been calculated as 0.659 or 65.9%. In this follow-up study 5 more novel risk markers, which are Carotid IMT, ABI, Brachial FMD, hs-CRP and Family History, were added to baseline model of FRS. In addition of those novel risk markers to FRS, NRI for those models of FRS + CAC, FRS + ABI, FRS + CRP and FRS + FH were measured as 0.659, 0.036, 0.079 and 0.160 respectively for incident CHD, and 0.466, 0.068, 0.037 and 0.040 respectively for incident CVD. As a result, this study showed, CAC has the highest improvement in recalibration of risk prediction and re-classification.

Yeboah et al. [56], a MESA study involving 6355 participants with mean follow-up time of 7.5 years, used Structural Equation Modeling (SEM) and conventional interaction analyses to analyze the relation between conventional cardiovascular risk factors and measures of subclinical disease including CAC score, logarithmic function of ln(CAC + 25), CIMT and brachial FMD. According to results of this study, ln(CAC + 25) was accounted with largest portion of risk associated with risk factors and is followed by CAC, CIMT and FMD, respectively. Ln(CAC + 25) could have share of risk associate with age and male sex as 80.2% and 52.2%. However, Yeboah et al. concluded that exception of associated risk with this two variables, majority of effects of traditional cardiovascular risk factors on risk for clinical events were not reflected in those subclinical disease measurements, current sublinical CVD markers might not be optimal intermediaries for gauging upstream risk factor modification and highlighted the need for improvement in quantifying the intermediate steps in the pathways from risk factors to clinical events. SEM as a multivariate statistical framework [57], was used for modelling complex relationship between directly observed subclinical measurements and indirectly observed(latent) variable for assessing the association between directly observed and multiple phenotypes of multiple phenotypes of interest as CVD-events.

Polonsky et al [58], as a MESA substudy including 5931 non-diabetic individuals with a median follow-up time of 5.8 years, reported NRI for the entire study cohort by adding CACS to the predictive model as 0.25 and NRI for events and non-events were reported as 0.23 and 0.02 respectively. Furthermore, improvement in risk stratification was observed more balanced between event and nonevents for intermediate risk individuals than the overall cohort, where 0.29 for events and 0.26 for nonevents were calculated as NRI of 0.55.

Valenti et al. [59], cohort study comprising 9715 consecutive asymptomatic individuals within mean follow-up time of 14.6 years, revealed CAC > 0 is strong predictor of death with HR of 2.67 (CI 2.29–3.11) compared to individuals with CAC = 0 and in presence of any CAC risk increase proportionally with severity of CAC score but independently of the FRS and NCEP, and without gender difference. Furthermore, CAC score of 0 was detected in association within lower vascular age contrasting with chronological age and this finding became more pronounced with increasing age and male gender, for instance ≥ 80 years old male patients with zero CAC were represented equivalent to the chronological age of a 50 year old man within general population, similarly patients with CAC = 0 had lower mortality rate than the equivalent category of chronological age in general population and the CAC = 0 score among individuals at low-to-intermediate risk presented at least 15-year warranty period, when annual mortality rate remains below 1% to define individuals in low risk, regardless of age and gender.

Budoff et al. [62], a MESA follow-up study consisting 6809 individuals with median observance of 3.75 years, discerned traditional absolute CAC cutpoints of 100 and 400 correspond quite closely to the 75′th and 90′th overall percentile according to the best fitting model and 75′th percentile and 90′th percentiles for the cohort were shown as 88 and 398, respectively. In comparison of percentile distribution and absolute score of ln(CAC + 1) by ROC-curve and R-squared test have shown log(CAC + 1) outperforming age, gender and race/ethnicity specific percentiles. However, Hoffman et al. [64], a cohort study involving 3238 Offspring and Third Generation subjects in Framingham Heart Study, revealed modest degree of disagreement between absolute and relative cutpoints in 12.6% and 17.6% of participants for Agatston Score > 400 vs 90′th percentile and Agatston Score > 100 vs. 75′th percentile, respectively.

Bertoni et al. [51], as a review study on MESA reports for DM, highlighted diabetes strongly associates with incidence and progression of CAC score and emphasized predictive strength of CAC as improving risk assessment more than any of other novel risk markers. However, they also mentioned that association between diabetes and CVD is not fully mediated through CAC score.

Consistently by Budoff et al. a prospective multi-ethnic study of 6814 participants (51% women) with median follow-up of 11.1 years and aged 45–84 years, showed CAC strongly predict ASCVD in same magnitude of effect regardless of gender, age groups, race, and ethnicity, and zero CACs carries a low 10 year risk and could be used to down-stratify a patient’s risk based on global risk assessment for possible reconsideration of aspirin and statin, however CAC score ≥ 100 indicates at least a 7.5% 10 year risk of ASCVD [46].

A MESA study with median follow-up of 11.0 years, a total of 3398 participants free of clinical event, [47] according to Cox models fully adjusted for individual CVD risk factors, where hazard ratio represent 1 SD of the independent variable, demonstrated ln CAC volume score adjusted for CAC density was a highly significant predictor of CHD and CVD with HRs of 1.73 (CI: 1.45–2.05) and 1.61 (CI: 1.39–1.85), respectively; and CAC density adjusted for lnCAC volume score at each SD increase significantly associate with 28% (CI: 0.60–0.86) risk reduction for CHD and 25% (CI: 0.65–0.87) risk reduction for CVD. Inverse relation between CAC density and CHD or CVD consistenly associated across levels of CAC volume and was most strongly observed in first quartile of volume. Furthermore, Net Reclassification Index Analysis showed adding CACS (Agatston) to conventional risk asessment (pooled cohort estimation-PCE) improved classification by 25% for both CVD and CHD, while highest reclassification was measured in model adding both lnCAC volume score and CAC density to PCE risk assessment at 35% for both CVD and CHD.

A MESA Analysis [49], involving 6814 participants with age range of 45 to 84 in a cross-sectional design, ln CAC volume score was discerned in positive association with age, male gender, college or greater level of education, total cholesterol, diabetes, antihypertensive medication use, BMI, family history of MI and amount of alcohol consumption, while inversely associate with high annual income and walking at brisk, and average paces. CAC density was shown in positive association with high-income, HDL-c, and brisk walking pace, while male gender, diabetes, and BMI were in inverse association.

A MESA study [13], enrolled 6814 adults without ASCVD, revealed prevalance of hard ASCVD and CHD events are higher among those with a FH of premature CHD compared to those without at an adusted HR’s of 1.35 (1.07–1.71) and 1.41 (1.05–1.88), respectively. Highest hard CHD events was observed in those with a FH of premature CHD and CAC ≥ 400 at 12.1 per 1000 person years. For patients with FH of premature CHD, patient group of CAC ≥ 400 had HR of 2.80 (1.44–5.43) relative to CAC = 0, similarly for patients without FH of premature CHD, patient group of CAC ≥ 400 had HR of 3.22 (2.14–4.84). However, any significant interaction between FH and CAC in the association with ASCVD events wasn’t observed (p = 0.28); moreover, any significant interaction between CIMT (either by percentile category or by the quartile) with ASCVD events based on FH status wasn’t observed (p = 0.21).

Durhan et al. [119], involving 2921 Turkish patients at mean age of 51 years in retrospective study design, showed CAC-score has superior predictive value for estimating critical stenosis risk compared to SCORE risk on ROC curve as AUC = 0.889 contrast with AUC = 0.773, and combined use of CACS and SCORE improved the risk estimation to AUC = 0.890.

## 8 Interpretation and reporting of CAC

Society of Cardiovascular Computed Tomography (SCCT) classify Agatston score by measurement intervals of 0, 1–99, 100–299 and ≥ 300 to risk levels as very low, mildly increased, moderately increase and moderate to severely increased, respectively [65]. For all noncontrast chest CT examinations SCCT recommends evaluation and reporting of CAC at Class 1 recommendation and thoracic aortic calcification at class 2b recommendation; moreover, for those imaging SCCT suggest reasonable to perform ordinal assessment of CAC at class 2a recommendation and Agatston CAC scoring at class 2b recommendation [65]. SCCT indicates estimating CAC score as none, mild, moderate or severe at class 1 recommendation [65]. SCCT defines CAC ordinal scores on 12 points and 30 points scales [65].

## 9 CAC-Development with progressing risk factors

In a MESA cohort sub-study including 5756 participants aged 45 to 84 years on average age of 62 years over an average of 2.4 years follow-up time [69], significant associations of several cardiovascular risk factors with risk of incident detectable CAC in RR and progression CACS in Agatson Unit have been measured respectively as per additional 10 years of age (RR: 1.39, 17.5 A), male gender (compared to female gender; RR: 1.43, 10.9A), ethnicity (ie. Chinese compared to Whites; RR:0.61, −9.4 A), BMI (RR:1.04, 0.9 A), 10 units of higher SBP (RR:1.07, 1.9 A), 10 units of higher DBP (RR:1.12, 2.6 A), use of antihypertensive medication (compared to non-user; RR:1.52, 8.0 A), 10 mg/dl of higher LDL (RR:1.03), 10 mg/dl of higher HDL (RR:0.89), logTriglyceride (RR:1.34, 6.6 A), use of lipid lowering medication (compared to non-user; RR:1.41, 9.8 A), former smoker (compared to never smoking; RR:1.22, 6.1 A), current smoker (compared to never-smoking; RR:1.29), impaired fasting glucose (RR: 1.35), untreated DM (RR:1.76, 14.4 A), treated DM (RR:1.66, 26.8 A), family history of CVE (RR:1.31, 9.0 A), and logCRP (RR:1.06, 2.6 A).

A cross-sectional study by Y. Ohmoto-Sekine et al. [105] as analysis of 1834 Japanese participants volunteered for simultaneous CAC scoring and lung cancer screening at one CT-imaging, demonstrated males in contrast with females CAC score increase by aging begins 10 years earlier with more gradual annual rate nonetheless cumulatively male gender significantly associated with higher CAC score per se, moreover, in univariate analysis for CACs >100 BMI, waist circumference, hypertension or medication, dyslipidemia or medication, diabetes or medication, fasting blood glucose, HbA1c ≥ 6.5%, eGFR < 60 mL/min/1.73 m^2^, uric acid ≥ 7.1 mg/dl, metabolic syndrome, smoking history and alcohol consumption by ≥ 20 g/day significantly associated yet in multivariate analysis of significant or near-significant (p < 0.2) covariates of univariate analysis only dyslipidemia (OR:1.74) and fasting glucose (1.01) along with aging per year (OR:1.10) and male gender (OR:2.46) remained significant with CACs > 100.

Spring et al. [111] including 3558 participants of CARDIA longitudinal study aged 18–30 years old at year 0 cohort baseline with re-examination on year 20 into cohort analysis, where evaluating odds of CAC > 0 as surrogate markers of subclinical atherosclerosis measurement depending on change in composite measurements of healthy lifestyle factors (HLFs) encompassing absenteeism from cigarette smoking, race and gender adjusted physical activity level ≥ 60th percentile, BMI < 25 kg/m2, limitation of alcohol intake ≤ 15 g per day for females and ≤ 30 g per day for males, and healthy diet correlating with low daily intake of saturated fatty acids and high daily intake of calcium, potassium and fiber, reported an uniform linear effect in CAC > 0 as per one unite change in HLF positively or negatively significantly associated respectively at rates of OR = 0.85 or OR = 1.17.

### 9.1 Physical activity

Greater physical fitness level has been shown to improve progression of CACS in a study with sample size of 678 aged 50–65 years [11] as patients with high fitness level have lower Odds ratio for CACS ≥ 100 compared with the low fitness level after adjusting for age, gender, familial history of CVE, education level, life exposure to smoking and perceived stress at OR of 0.47 (CI: 0.23–0.96). Malik [121] reported continuous association between METs (Metabolic Equivalents of Task) and CAC-score, where at each 1 MET increase in exercise capacity results 66.2 AU lower CAC-score, however, for moderate intensity exercise (≤8.2 METs to < 10.6 METs) CAC-score remained similar with increasing minutes of exercise per week. Kleiven [122] demonstrated hours of endurance training per week had no association with progression of CAC, nevertheless correlated well with reported MET-h/week. Kermott [123] observed U-shaped distribution of CRF, which is defined by Functional Aerobic Capacity (FAC) measured in treadmill testing, on CAC-score across which those with FAC of 100% to 129% (Age:52.7 years, FRS:7.1, MET:12.2) had significantly lower CAC-score levels compared with FAC of ≤ 69% (Age:51.9 years, FRS:8.0, MET:7.2) and ≥ 130% (Age:55.4 years, FRS:8.2, MET:14.8) regardless of family history (FH) of CAD/premature CAD; however, those with highest CRF category of FAC ≥ 130% had lower level of LDL and Blood Glucose and higher level of HDL, moreover, in subgroup analysis of patients with ≥ 60 years of age adjusted with FH of CAD higher FAC categories associate with lower FRS, and consistently in initial overall-analysis participants grading FAC ≥ 130% had higher mean-age and rate of FH of premature CAD to a trend of higher FRS. Aengevaeren [124] observed each MET-hour/week associated with higher prevalence of CAC > 0 (OR = 1.02), across exercise volumes (<1000, <2000 and > 2000 MET-min/week) CAC-score categories (0, ≤100 and > 100 AU) significantly increase on which particularly for lifetime exercise volume of > 2000 MET-min/week vs < 1000 MET-min/week associated with higher prevalence of any CAC > 0 (OR = 3.2), higher CAC scores (9.4 vs 0), higher calcification (CAC) area (4.3 vs 0), greater number of calcification regions (2vs0), higher plaque prevalence (77% vs 56%), lower prevalence of mixed plaque (48% vs 69% with OR = 0.35), more likely only calcified plaque (OR = 3.57), and plaque prevalence and plaque morphology change into less common mixed plaque were only mediated by very vigorous and vigorous exercise intensities in positive graded response, respectively. Malik [121] noted no adverse effects of increasing either exercise-volume (duration) or exercise intensity (moderate or high intensity) on CAC-score. Hamer [125] observed participants with CAC = 0 compared to CAC ≥ 400 completed 8-ft walking course 0.16 s faster, and objectively assessed faster walking speed associated with lower risk of having CAC ≥ 100 (OR = 0.62). Susan et al. [83] reported per 1-SD increase in CACs (138.8 AU) significantly associated with annual 0.003 point-score greater decrease in self-reported walking pace yet remained similar across CACs categories.

According to Ekblom-Bak et al. Presence of MetS (Metabolic Syndrome) increase prevalence of CACS ≥ 100 72% compared with no MetS [11]. By Arnson et al. [68], a follow-up study involving 10,690 asymptomatic patients with mean age 55.7 ± 11.0 years along mean follow-up time of 8.9 ± 3.5 years, showed that amount of exercise was in proportional inverse association with risk factors including hypertension, diabetes, statin use, smoking, family history of premature CAD, LDL, VLDL, triglyceride values, BMI, blood pressure measurements, fasting blood glucose levels, and resting heart rate; moreover median CAC score, and frequency of CACS ≥ 400 and mean number of CAC plaques were highest in the no-exercise group; whereas highly active group had highest median HDL. Jae et al. [126] presented those with CMS (Cardiometabolic Syndrome) compared to without CMS had more CVD-risk factors, increased risk of SCVD, higher levels of CRP (0.06 vs 0.09 mg/dL) and CAC-score (2 vs 6 AU), lower peak HR (152vs150) and VO_2_peak (31.8 vs 29.9) both indicating low fitness, higher risk of CAC > 0 (OR = 1.41) in which number of CMS components correlated with presence of CAC > 0; nonetheless higher fitness levels attenuate these associations at which for each 1 MET (Metabolic Equivalents of Task, 3.5 mL/kg/min) increase in fitness associated with 10% lower odds of having CAC > 0 and for fit individuals regardless of having CMS prevalence of CAC > 0 remained similar.

In a prospective cohort sub-study of CARDIA (The Coronary Artery Risk Development in Young Adults) [60] recruiting 4872 young adults aged 18–30 years with median 26.9 years of follow-up, after full adjustment for age, race, sex, obesity, CVD risk factors, and LV mass index each additional minute of exercise test duration associate with 15% lower hazard of all-cause mortality (CI: 0.80–0.91), 12% lower hazard of incident CVD (CI: 0.81–0.96), 24% lower beta value for LV mass index (at year 25 of follow up; CI: −0.45 to −0.03), and 9% lower beta value for global longitudinal strain (at year 25 of follow up; CI: −0.14 to −0.05); however, exercise test duration was not associated with CAC at follow-up years of 15, 20, and 25. LF-Defina et al. [127] reported men with accumulated high level of PA (Physical Activity) of ≥ 3000 MET-min/week compared to reference group of men with low level of PA < 1500 MET-min/week significantly associated with decreased risk of all-cause mortality for score categories defined by any CAC-score ≥ 1 (HR = 0.63) or CACs < 100 (HR = 0.52) yet similar for CACs ≥ 100, and in response to increased PA-level increased risk of having CAC ≥ 100 (RR = 1.11), weakly physical activity duration and absolute MET-min/week, cardiorespiratory fitness, V_O2max_, proportion of V_O__2__max_ ≥ 50 mL/kg/min and HR-recovery at 1 min also lowered maximum-HR; moreover, across PA categories (<1500, <3000 and ≥ 3000 MET-min/week) specifically CVD mortality rates remained similar in CACs categories through cut-offs of 1 AU or 100 AU at which for moderate level of PA CVD and even all-cause mortality rates remained similar compared to those with low PA level, nonetheless, for participants with < 100 AU high vs low PA level significantly associated with lower odds of all-cause mortality.

Shah et al. [60] considered cardiovascular benefits of fitness extends beyond prevention of CAC and it’s progression, and in concise subclinical CVD-CAC as an important biomaker may not fully capture the benefits of fitness on cardiovascular health. Rozanski et al. [128] noted no significant difference in mortality rates per 1000 person years between CACS ≥ 400 with high self-reported PA level vs CACS < 100 with low self-reported PA level. Kermott et al. [129] discerned a CAC Score ≥ 100 rather than CACS < 100 can be significantly predicted by combined explanatory variables of Framingham score and self-reported exercise intensity level. Radford et al. [130] reported total CVD-incidence rates in CAC ≥ 400 vs CAC = 0 stratify with CRF (Cardiorespiratory Fitness) of 5 METs and 15 METs as 5-fold and 2-fold increase.

Arnson [68] noted combined effect of CACS and reported exercise activity on risk of ACM (Annual Cardiovascular Mortality), there was no significant difference in survival according to reported exercise among patients with a CAC score of 0; however, among participants with CAC score of 1 to 399 most sedentary individuals associated with decreased survival and among patients with CACS ≥ 400 survival rates decrease progressively with each level of decreasing physical activity. Rozanski et al. [128] revealed relation of self-reported physical activity (PA) level and all-cause mortality (ACM) varies with CAC-score categories that a CAC score of 0 to 99 sustained similar low ACM across low, moderate and high PA levels, in CAC score category of 100 to 399 only low vs high PA category had significantly greater risk of ACM (OR:2.07), and in category of CACS ≥ 400 progressively both moderate vs high (OR:1.68) and low vs high (OR:2.35) associated with increased risk of ACM. Radford [130] observed at each additional MET of CRF associates with 11% lower risk of CVD-events, moreover higher CRF attenuates CVD-events risk associates with higher CAC in any 4 CACs-categories defined by cut-off scores of 0, 100 and 400. Malik [121] showed in time-to-event analysis among participants with CACs ≥ 400 those achieving ≥ 8.2 METs (along median exercise volume of 240 min/week or ≥ 10.6 METs along median 213 min/week; around ≥ 2000 METs-min/week) compared to < 8.2 METs had significantly fewer CVD-events, yet a non-significant trend toward lower CAC-score. Similarly, Rozanski et al. [128] discerned those with CACs ≥ 400*High-PA vs CACs0-99*Low-PA had statistically similar mortality rate per 1000 person-years (19.9 vs 16.3 per 1000 person-years) and multivariable nominal logistic regression revealed Aging (per 5 years, HR:1.70), Low vs High PA (HR:1.55), CACs ≥ 400 vs CAC = 0 (HR:1.56), DM (HR:1.39) and smoking (HR:1.39) significantly associated with all-cause mortality.

### 9.2 Dietary factors

#### 9.2.1 Change in CAC-s related with Macronutrient intakes

A cohort MESA sub-study by Hu et al. [70] including 5614 participants with mean age of including 5614 participants with mean age of 62.5 years, reported no significant trends across the overall Low Carbohydrate Diet score quintiles, which are defined by assigned decile points particular to respective macronutrient intake intervals and overall-LCD score is the sum of decile numbers of respective total carbohydrate, protein and fat intake, with either the incidence of detectable CAC or the progression of CAC among those with baseline of CAC > 0, regardless of the sources of protein and fat as animal-based, which includes higher intake of dairy-products and saturated fat yet lower intake of vegetables, or plant-based, which includes higher intake of vegetables, nuts and monounsaturated/polyunsaturated fat yet lower intake of dairy-products.

A cohort study by Sung et al. [71] enrolling 10,793 healthy Korean adults with mean age of 40.7 years, demonstrated that highest energy intake group and highest fat intake group had a lower rate of subjects with non-zero CAC score, while highest carbohydrate intake group had the highest percentage of subjects with CAC; however, in multiple regression analysis they illustrated that CAC scores were no significantly different among tertile groups of each macro-nutrient intake, however, the study population in cross-sectional design includes considerably young and relatively healthy occupational subjects of whom high-energy intake positively correlate with physical activity, and study didn‘t evaluated qualitative values of macro-nutrient intake.

Gao et al. [131] observed an inverse non-linear association of carbohydrate intake with CAC-s progression which is most pronounced for male-vs-female genders and white-vs-black ethnicities, across 3 categories of carbohydrate intake as percentage intervals of total energy (low < 43%, moderate 43%-53% and high ≥ 53%) most significant difference in risk of CAC-progression was for those High-vs-Low carbohydrate intake (HR = 0.731, 95% CI:0.552–0.968) at which in LCD-score (Low Carbohydrate Diet Score) analysis, as the summary for intake of carbohydrate and either animal or plant sourced protein and fat, animal-based LCD score significantly associated with CAC-progression (HR = 1.456, p = 0.041) yet plant-based LCD score remained similar for risk of CAC-progression (HR = 1.016, p = 0.884).

A cohort study by Rozanski et al. [132] including 15,368 participants with median follow-up period of 12.1 years to examine relations of self-reported frequencies on 10-unit scale for physical activity and intake of low-saturated fat intake, which was subsequently categorized inversely to 4 saturated-fat score categories noted as low[8,10], moderate[6,7], high[3,5] and very-high[0,2], with CACs and serum lipid panel, observed a significant stepwise trend across increasing self-reported saturated fat consumption categories with higher levels of cholesterol, LDL and TG, and lower HDL and physical activity yet no significant difference for CACs, moreover, by Kaplan-Meir survival curve participants with self-reported very-high saturated-fat category had lowest survival rate in adjusted model (of age, gender, hypertension, diabetes, dyslipidemia and smoking) but further adjustment for self-reported physical activity attenuates these associations, nonetheless, survival rate differences among 4 saturated-fat categories were modified with CACs categories (of 0, 1–399 and > 400 AU) as gaining strength with increasing CACs category and for CACs > 400 a stepwise decrease in survival as high and very-high vs low/moderate saturated-fat categories.

#### 9.2.2 Dietary patterns

A cross-sectional study by Gripeteg et al. [95], including 706 Swedish adult participants to analyze compound association of Healthy Food Index (HFI) compiled from consumption frequencies of 17 food items in 5 groups (as vegetables, fruits, nuts, vegetable oils of olive and canola, and fatty fish) and Cardiorespiratory Fitness Index (CRF) predicted by the Eklom-Bak equation derived from quotient of difference in heart rate over power, with CAC score demonstrated HFI significantly associated with higher rates of zero-CAC against non-zero CAC, and interaction of HFI and CRF significantly negatively associated with CAC-score in regression analysis.

Talaei et al. [96], a follow-up study recruited 57,078 Chinese adults aged 45 to 74 in Singapore with a mean follow-up of 17.2 years, presented the DASH score, obtained through participants reported frequencies of consumption for 7 food groups and 1 mineral (as high intake of fruits, vegetables, nuts, dairy products, and whole grain and low intake of sodium, red meats and sugar-sweetened beverages), significantly associated with lower risk of CVD, CAD, and stroke mortality; however after interaction of current smoking with adherence to DASH-diet, which significantly correlated with higher intakes of calcium, potassium, magnesium, fiber, folate, vitamin D, and polyunsaturated versus saturated fatty acids, those significant associations weakened and became borderline-significant as p = 0.08 for CVD and stroke mortalities, and nonsignificant as p = 0.7 for CAD mortality.

Frolich et al. [133] examining the relation between dietary patterns (Animal fat/alcohol, Health-conscious, Traditional German/less alcohol, Mediterranean-like, Western) and progression of CAC, reported compared to reference group of/with animal fat/alcohol dietary pattern smaller risk for rapid progression in CAC-score after 5 years of follow-up as exceeding > 120% of expected CAC-score percentile, which was defined by a formula derived in Heinz-Nixdorf Recall Study (Erbel, 135) to predict age and gender-specific CAC-score percentile changing over baseline CAC-score percentile, was most significantly observed for those with Mediterranean-like dietary pattern for both genders (Male RR = 0.61 and Female RR = 0.59), moreover, among females Health-conscious (RR = 0.63) and Traditional German/less alcohol (RR = 0.69) dietary patterns also associated with reduced risk for rapid progression of CAC percentile score yet Western dietary pattern remained similar.

Anderson et al. [134] presented over longitudinal 10 years of follow-up by fully adjusted model, reported among subjects with no CAC-s at baseline through 5 dietary calcium intake quintiles, highest vs lowest associated with decreased risk of CAC > 0 incidence (RR = 0.73, 95% CI:0.57–0.93) yet calcium supplement use attenuates this significant association (RR = 0.74, CI:0.51–1.07) besides in the same fully adjusted model calcium supplement use associated with 22% increased risk of having CAC > 0 incidence across which for calcium intake of combined quintiles 2-to-5 of calcium supplement use risk of having CAC > 0 remained similar but for lowest quintile calcium intake through supplementation use posed significant risk (RR = 1.41, CI:1.02–1.97); and for participants with CAC > 0 baseline dietary intake of calcium didn‘t increase the risk of CACs progression (Log-transformed CACs changes within inter-scan period).

#### 9.2.3 Specific food items

A cross-sectional study by Chun et al. [93] involving 22,210 Korean adult participants on median age of 40 years and comparing consumption of sugar-added (sweetened) beverages versus no or low intake, demonstrated higher intake associated with younger-age, male-gender, current-smoker, physically active, higher probability of college-degree attainment, and higher odds of hypercholesterolemia history and family history of premature ASCVD; and compared to non-drinkers attendants with ≥ 5 sugar-added carbonated beverages per week had significant 1.86 CAC score ratio and 1.27 odds ratio of having non-zero CAC score.

Sekikawa et al. [94] studying association of two marine-omega-3 fatty acids (OM3) of Docosahexaenoic acid (DHA) and Eicosapentaenoic acid (EPA) with coronary artery calcification (CAC) and CAC Density score (CDS) in cross-sectional design recruiting 1086 male patients aged 40 to 79 from Japanese general population, where OM3 consumption is very high, observed only Docosahexaenoic acid (DHA) significantly inversely associated with Coronary Calcium Score (CCS) but not with CDS, however, only serum EPA significantly inversely associated with CDS after model adjustment for age, used CT-device type, CAC score, hypertension, diabetes, LDL, HDL, smoking pack-year, and BMI, along with for CRP, triglycerides, lipid-lowering medication, and histories of CVD and CKD.

A case controlled retrospectively matched cohort study by Feuchtner et al. [224] including 53 patients taking Omega-3 supplementation (either DHA or EPA at 1 g) with mean duration of 38.6 ± 52 months and their propensity score matched 53 control participants according to no intergroup difference in age, gender, BMI, conventional risk factors, ASA and statin medications to examine influence of Omega-3 (n-3) polyunsaturated fatty acids (PUFA) on CAD plaque burden assessed by CTA, reported Omega-3 group compared to control group had significantly lower G-score of mixed noncalcified plaque burden (4.5 vs 7.4), lower prevalence of high-risk plaque (HRP) burden (3.8% vs 32%) and lower number HRP-count (3vs23), higher mean intraplaque CT-density of low-attenuated plaque at either HU of region of interest (131.6 vs 62.1) or HU of lens (132 vs 52.1) reflecting more like denser fibro-calcific other than lipid-rich changes, higher spotty calcification (18.8% vs 11.3% or 1 case vs 6 cases) and but lower rate of napkin ring sign (3.7% vs 20.9%) reflecting lipid-rich necrotic core, but stenosis severity remained similar for both groups.

Ghosh et al. [135] showed self-reported whole-milk consumer ≥ 1 time/month vs rare/never whole-milk consumer < 1 time/month associated with lower CAC-s baseline (43.7% vs 50.9%), lower CAC-s progression (OR = 0.836, p = 0.009), lower traditional risk factors and higher SFA (Short-Chain Fatty Acids) levels of which caproic acid, butyric acid and caprylic acid have significantly higher plasma levels yet only caproic acid mediates reduced CAC-s progression (23.7%, c‘=-0.238, p = 0.034).

Choi et al. [115], including 23,417 asymptomatic Korean adults from the Kangbuk Samsung Health Study into cohort analysis, demonstrated eggs consumption ≥ 7 eggs/week significantly associates with higher prevalence of any non-zero CAC > 0 (OR:1.80), CAC 1–100 (OR:1.21) and CAC > 100 (OR:1.75) compared to reference egg consumption < 1 egg/week, and each increase of 1 egg/day associates with higher prevalence of any non-zero CAC > 0 (OR:1.54), CAC 1–100 (OR:1.16) and CAC > 100 (OR:1.36), moreover, those positive associations becomes stronger among participants with higher BMI and lower vegetable intake.

However, Qin C. et al. [116], recruiting almost half a million Chinese adults aged 30–79 years followed along period of median 8.9 years on ongoing China Kadoorie Biobank (CKB) cohort study, observed a significant trend of increasing egg consumption stratum, such as 1 egg each day compared to almost non-consuming per week, associates with lower risk of CVD (OR:0.89), ischaemic heart disease (IHD; OR:0.86), major caronary event (MCE; OR:0.74) and ischaemic stroke (OR:0.90); and similarly egg consumption strata inversely associated with lower mortality rates caused by CVD (OR:0.82) and haemorrhagic stroke (OR:0.72).

A randomized trial by Zeb et al. [225] including 65 participants aged mean 60 ± 9 years at intermediate risk randomized to experiment group receiving Aged Garlic Extract (250 mg) + Supplements of Vitamin B6 (12.5 mg), Vitamin B12 (100 µg), Folate (300 µg) and L-arginine (100 mg) and placebo group on 1-year of follow-up to investigate potential effects of experiment intervention on regional fat depots of EAT (Epicardial Adipose Tissue), PAT (Pericardial Adipose Tissue), PaAT (Periaortic Adipose Tissue) and SAT (Subcutaneous Adipose Tissue), reported experiment group compared to placebo group had significantly lower EAT (OR:0.63), PAT (OR:0.72), PaAT (OR:0.81) and SAT (OR:0.87) in multiple logistic regression model (adjusted for age, gender, DM, HTN, Hypercholesterolemia, FH of premature-CHD, smoking status, statin therapy and BMI).

#### 9.2.4 Dietary blood workups

Won [136] showed TyG index, which is formulated by exponential function of Ln(Triglyceride[mg/dl]*Glucose[mg/dl]/2) as the surrogate marker of Insulin Resistance (IR) across it‘s increasing tertiles positively correlated with incidence of CAC progression, which is defined by difference of ≥ 2.5 over baseline in square-root of CAC-score (Δ√Transformed CAC-score) through follow-up, Δ√Transformed CAC-score and Annualized-Δ√Transformed CAC-score; however, in adjustment for categorical CAC-score across TyG tertiles and TyG index per 1 unit increase CAC progression and Annualized Δ√Transformed CAC-score remained significant for those with baseline CAC-s ≤ 100.

Park [137] demonstrated across TyG index Δ√Transformed CAC-score and Annualized-Δ√Transformed CAC-score and risk for CAC progression and incidence of CAC > 0 increased.

Generoso [138] in cross-sectional analysis observed when adjusted with TG-level per 1-SD decrease in Total HDL-c, HDL_2_-c and HDL_3_-c and HDL_2_-c/HDL_3_-c ratios have no significant association with Ln(CAC + 1), having CAC > 0 vs CAC = 0, having CAC ≥ 100 vs CAC < 100 and ln(CAC), whereas adjustment with LDL-c didn t attenuated relations of Total HDL-c, HDL2-c and HDL3-c with either Ln(CAC + 1) or CAC > 0 vs CAC = 0.

Bittencourt [139] demonstrated per 1 SD increase in Log-transformed Triglyceride-rich Lipoprotein (Ln[TRL-C], OR:1.20), LDL-c (OR:1.28) and Non-HDL-Cholesterol (OR:1.36) associated with increased OR for CAC > 0 in multivariate logistic regression model with adjustment for age, gender, DM, HTN, SBP, LDL-c, Ln[TRL-C], HDL-c, BMI and Ln[hs-CRP].

YM Eun [140] in retrospective study design based on medical records illustrated fasting plasma glucose (FPG) across prediabetic (Impaired Glucose) range significantly associates with higher risk of having CAC > 0 at which compared to subjects with FPG < 100 mg/dl those with either FPG ≥ 110 mg/dl or those with 110 ≤ FPG[mg/dL] < 120 (OR:2.50) and 120 ≤ FPG[mg/dL] < 126 (OR:3.57) had higher odds after adjustment for age, gender, BMI, WC, SBP, DBP, smoking, AST, ALT and rGTP, moreover, with same multivariable logistic regression model higher BMI, SBP, TG, LDL and rGTP posed significantly higher odds.

### 9.3 Cigarette use

A cohort study by Leigh et al. [141] including 3356 participants with ≥ 100 cigarettes use in lifetime aged mean 62.1 ± 9.9 years with mean 11.1 ± 2.9 years of follow-up examining predictivity of CAC and PCE seldom or combined for CVE among both over-all cohort and lung cancer screening eligible participants without signs or symptoms of Lung CA, which was defined as being aged 55 to 77 years, current smoker or former smoker who has quit within last 15 years and having tobacco smoking history of ≥ 30 pack-years, reported pack-years of smoking in CAC-score categories of CAC = 0 and CAC 1–300 correlated with increased risk of ASCVD-events yet for those with CAC > 300 pack-years of smoking correlated with fewer risk of ASCD-events, moreover, according to C-statistics of ROC-curve analysis addition of log-transformed Ln[CAC + 1)] into PCE (Pooled Cohort Estimation) model improved predictivity of ASCVD-events for study population but for those with LCSE predictive value of compound model remained similar and marginally weak besides the same compound model didn‘t improve NRI (Net Reclassification Index) either for study population or for those with LCSE, nonetheless for events groups of both overall-study and LCSE-participants compound model improved re-classification as 1.8% and 16.0%, respectively, furthermore, for participants with baseline CACs = 0 6.7% in overall-cohort and 14.2% in LCSE subset participants had ASCVD-events during follow-up, as a summary these findings suggest down-stratification by CACs = 0 may not be applicable for patients with smoking history but particularly for LCSE-patients.

Al-Rifai [142] in cross-sectional analysis including 9411 participants without lipid-lowering treatment aged mean 57 years examining correlates of smoking, which was noted with causing morphological and functional damage to endothelium, vasomotor dysfunction, inflammation, IR and oxidation of atherogenic lipoproteins, in SCVD observed smoking status of ever smoking associated with increased odds of having CAC > 0 among participants with untreated LDL-c < 70 mg/dL calculated by using either Friedewald Equations (OR:1.92, 95% CI:1.02–3.62) or Martin/Hopkins Equations (OR:2.29, 95% CI:1.02–3.62) in multivariable adjusted model-2 (of age, gender, ethnicity, education, study site and conventional risk factors); furthermore, in multivariable adjusted model smoking burden through per 1 SD increase in pack-years of smoking significantly associated with increased odds of having CAC > 0 among participants with untreated LDL-c < 70 mg/dL by Martin/Hopkins Equations (OR:1.55, 95% CI:1.07–2.23).

Hisamatsu et al. [117], recruiting 1019 the Shiga Epidemiological Study of Atherosclerosis (SESSA) participants derived from general Japanese population aged 40 to 79 years into the study analysis, revealed current-smoking excepts for former-smoking significantly associated with higher prevalence of any non-zero CAC > 0 (OR:1.79), CAC ≥ 100 (OR:2.06) and CAC ≥ 400 (OR:2.64), cumulative exposure through pack-years of smoking associated with higher prevalence of non-zero CAC > 0; and attenuation of subclinical atherosclerosis as returning to normal never smoking status takes ≥ 10.4 years at a non-zero 1 < CAC < 100 score, and ≥ 24.4 years at both 400 > CAC ≥ 100 and CAC ≥ 400 scores.

Hisamatsu et al. [117], recruiting 1019 the Shiga Epidemiological Study of Atherosclerosis (SESSA) participants derived from general Japanese population aged 40 to 79 years into the study analysis, revealed current-smoking excepts for former-smoking significantly associated with higher prevalence of any non-zero CAC > 0 (OR:1.79), CAC ≥ 100 (OR:2.06) and CAC ≥ 400 (OR:2.64), cumulative exposure through pack-years of smoking associated with higher prevalence of non-zero CAC > 0; and attenuation of subclinical atherosclerosis as returning to normal never smoking status takes ≥ 10.4 years at a non-zero 1 < CAC < 100 score, and ≥ 24.4 years at both 400 > CAC ≥ 100 and CAC ≥ 400 scores.

A follow-up study by Corrall et al. [143] recruiting 3189 participants among CARDIA cohort study with 25 years of follow-up period from baseline to outcome examination investigating interactions of cumulative smoking and alcohol exposures and cumulative depressive symptoms with CACs, demonstrated at outcome examination cumulative alcohol use as drink-years and smoking as pack-years had graded increasing response with increasing CACs severity-group, moreover, in multinomial logistic regression at each 10 pack-years of smoking significantly associated with CACs 1–99 vs CACs = 0 (OR:1.35) and CACs ≥ 100 vs CACs = 0 (OR:1.46) besides cumulative Smoking*Depressive symptoms term had significant interactions for CACs ≥ 100 and CACs 1–99, furthermore, among overall study population compared to participants with CACs = 0, packyears = 0 and TWA-AUC CES-D = 0 for any pack-years of smoking > 0 increasing levels of cumulative depressive symptoms across TWA-AUC CES-D scores 2, 10 and 16 associated with greater odds of CACs ≥ 100 vs CACs = 0 and CACs 1–99 vs CACs = 0 besides these association become stronger with increasing level of pack-years of smoking reaching OR of 3.40, 4.82 and 6.25 for CACs ≥ 100 and OR of 2.60, 3.28 and 3.91 for CACs 1–99 respectively at 30 pack-years of smoking, nonetheless, in multinomial regression analysis adjusted for Alcohol Use*Depressive Symptom interaction term Smoking*Depressive symptoms modification remained significant only for CACs ≥ 100 vs CACs = 0 and Alcohol Use*Depressive symptoms modification had significant interaction for only CACs 1–99 vs CACs = 0 yet per 10-pack years of smoking remained significantly associated with CACs 1–99 and CACs ≥ 100, moreover, compared to CACs = 0, Pack-years = 0 and CES-D = 0 for both increasing pack-years > 0 of 10, 20 and 30 years and across CES-D scores of 2, 10 and 16 associations with CACs 1–99 vs CACs = 0 and CACs ≥ 100 vs CACs = 0 were more obvious with greater odds among participants with drink-years = 0 compared to median alcohol use of drink-years = 8 reaching highest OR = 9.10 at pack-years = 10*CES-D = 16 for higher risk CACs ≥ 100 excepts pack-years = 10*CES-D = 16 for moderate risk CACs 1–99 as non-significant trend, in addition, among participants with 8 drink-years and 0 pack-years risk moderate-risk CACs 1–99 inversely associated with grading CES-D = 2 (OR:0.96), CES-D = 10 (OR:0.80) and CES-D = 16 (OR:0.70) compared to reference group; but in overall these findings suggest cumulative smoking level synergistically with cumulative depressive symptoms is contributory to CAC-development, which was explained with shared overlapping mechanisms of dysfunctional anti-inflammatory response or systemic inflammation, endothelial dysfunction, oxidative stress and behavioral mechanisms.

Another report of the reviewed cohort study by Corrall et al. [144] with same study design and participants but examining interactions of different clusters of cumulative depression symptoms (negative affect, anhedonia and somatic symptoms) with cumulative smoking exposure in CACs at year-25 outcome examination, reported each depressive symptom cluster and cumulative smoking exposure significantly but weakly correlated as negative affect (r = 0.077), anhedonia (r = 0.037) and somatic symptoms (r = 0.066), moreover, depressive symptoms subscales*smoking interaction remained significant for only somatic symptom cluster and CES-D, nonetheless, in logistic regression models for each depressive symptom clusters and CES-D adjusted for sociodemographic (gender, ethnicity and age), clinical (TC, SBP, DBP, BMI and Diabetes) and behavioral covariates (physical activity, alcohol use and depressive symptoms) at 25% cluster and CES-D scores with increasing cumulative smoking exposure > 0 of 10-packyears, 20-packyears and 30-packyears significantly associated with CACs > 0 through increasing odds compared to reference participants with CACs = 0 and 0-cluster score and most evident association was represented for somatic symptom clusters then CES-D score and both scores reached highest odds at 50% cluster score with 30-packyears as OR:6.68 and OR:5.74, respectively, however, main effects of symptoms clusters couldn‘t reach significance with CACs without cumulative smoking exposure, whose main effect had significant association with non-zero CACs (OR:1.42), and these results suggest confirming synergistic interaction of cumulative smoking exposure with cumulative depression symptoms for CACs > 0 incidence.

According to Miranda et al. [72], which is a cross sectional study comprising 4426 individuals, coffee consumption (>3 cups/day) inversely associate with CACS ≥ 100 at odds ratio of 0.33 (95% CI: 0.17–0.65) after adjustment; however, deleterious effect of cigarette consumption overwhelms the benefits of coffe intake and only never smokers associate significantly with lower odds of coronary calcification (odds ratio:0.37, where CI: 0.15–0.91) for habitual coffe intake up to 3 cups/day.

Kianoush et al. [73] argued that cigarette smoking causes both acute and chronic cardiovascular risk, which necessitate different measures of exposures and biomarkers, moreover, they suggested hsCRP for studying inflammatory subclinical domain [75] due to acute exposure of tobacco, and CAC score or presence of CAC to detect asymptomatic coronary atherosclerosis that heralds evident CVD [74] after a given long latency period. According to their cohort study, after full multivariable adjustment, compared with never smokers, current smokers had higher ln-transformed hs-CRP (beta = 0.24), 70% higher odds of having hsCRP ≥ 2 mg/L and 83% higher odds of CAC > 0, although no association had been observed among former smoker for those parameters. However, among ever smokers, they observed that each 5-year increase in pack-years had associated with higher levels of ln(hs-CRP) (beta = 0.02) and 10% higher odds of CAC > 0, moreover, each 5 year period passed since quitting smoking had associated with lower ln(hs-CRP) (beta = -0.03) and 13% lower odds of CAC > 0. Similarly, they had illustrated that for each 10 cigarettes/day increase in smoking associates with higher level of ln(hs-CRP) (beta = 0.10) and 26% higher odds of CAC > 0. Similarly on relation between cigarette consumption and subclinical cardiovascular disease, McEvoy et al. [75] reported that compared to never smokers, current smoker had 80% higher odds of hs-CRP ≥ 2 mg/L and former smokers had 20% higher odds of hs-CRP ≥ 2 mg/L. Participants with highest quartile of pack years compared to it’s lowest quartile had higher prevalence of hs-CRP ≥ 2 mg/L at odds ratios of 1.7 (CI: 1.3–2.2) and 2.6 (CI: 1.7–4.1) among former smokers and current smokers, respectively. They observed that current smokers had 38% higher odds of CAC score > 75′th percentile compared to never-smokers, and for each 5-year interval of abstinence from smoking former smokers had lower odds ratio of CAC > 0 as 0.94 (CI: 0.90–0.97).

### 9.4 Alcohol consumption

A cross-sectional study by Ogunmoroti et al. [77] selecting 6506 participants of MESA-cohort aged 62 years of age examining relations of questionnaire acquired Life’s Simple 7 (LS7) metric measurements (including parameters of BMI, Physical Activity in MET-min/week, Healthy Diet Score, Total Cholesterol, SBP, DBP, and FBG), which were evaluated per parameters as poor, intermediate and poor, with alcohol-intake status and level, reported across alcohol use levels (never-drinker, former-drinker, <1 drink/day, 1–2 drinks/day, >2 drinks/day and binge drinking) in graded-significant trend healthy diet score decreased and proportion of current-smokers increased, moreover, never-user vs > 2 drinks/day at 5 LS7-metrics excepts PA and BG and former-user vs > 2 drinks/day at 5–6 LS7-metrics excepts BMI and PA had greater proportion of ideal point, furthermore, in multivariable model adjusted for socio-demographic factors (of age, gender, ethnicity, education, income and health insurance) compared to reference never-user category among overall-study population former users, <1 drink/day and 1–2 drinks/day had no significant benefit for average vs inadequate and optimal vs inadequate CVD-Health and those with > 2 drinks/day had significantly lower CVH for average vs inadequate (OR:0.61) and optimal vs inadequate (OR:0.29) yet these relations interacts with gender and among females > 2 drinks/day had no significant change in CVH, <1 drinks/day with average vs inadequate CVH (OR:1.33) and 1–2 drinks/day with optimal vs inadequate CVH had significant associations, nevertheless, as implication authors discussed suggesting 1–2 drinks/day to anyone could be inappropriate with warning high risk of addiction potential besides noting usual short-term problems with alcohol and worldwide alcohol-related disease and injury burden.

Study by Chevli et al. [145] as a cross-sectional observation with standardized questionnaire administered in interview, which measures alcohol intake on mutually exclusive intervals of never, 1–3, 4–7 and > 7 drinks/week, including 706 South Asian male participants, demonstrated compared to never drinkers those with > 7 drinks/week had lower odds of having intermediate (OR:0.31, 95% CI: 0,10–0.93) and ideal (OR:0.14, 95% CI:0.03–0.60) cardiovascular health on Life‘s Simple 7 (LS-7) metrics yet this association interacted with age and those with < 58 years of age CVH remained similar with alcohol intake, nevertheless those with binge drinking (≥5 drinks in a single day within last month) compared to never drinkers had significantly lower odds for ideal CVH (OR:0.03, 95% CI: 0.003–0.36) regardless of age group.

A cross-sectional analysis by Mahajan et al. [146] with self-administered standardized questionnaires recruiting around 1000 healthy middle-aged males (aged 40 to 49) from ERA-JUMP Study registry as 300 participants from US Whites and Japanese American equally, 100 US Black and 300 Japanese living in Japan, demonstrated heavy alcohol consumption (>3 drinks/day in which 1 drink equals to 12.5 g of alcohol equivalent to either 350 mL regular beer, 150 mL of wine, 45 mL distilled sprit or 110 mL of sake) significantly associated with CACs progression in Tobit conditional regression analysis at which Ln[CAC + 1] is outcome variable through linear trend (OR:2.60, 95% CI:1.14–5.96), and in ordinal logistic regression analysis alcohol consumption level had quadratic trend of having more severe CACs (OR:2.44, 95% CI:1.32–4.51) on 4 CACs categories (0–9, 10–99, 100–299 and ≥ 300), moreover, light drink (≤1 drink/day) or moderate drink (more than > 1 drink/day and less than ≤ 3 drink/day) had null association with CACs progression in either Tobit conditional regression or Ordinal logistic regression in which invalidate U or J shaped association between CACs and alcohol intake as suggesting no health benefit of light-to-moderate drink; while authors note limitations for lack of examining drinking patterns, underreported alcohol use, which may be to evade social embarrassment, and remaining possible residual confounders.

Chevli [147] including 906 South Asian participants with structured interview questionnaire in cross sectional design showed compared to never drinkers those with 4–7 drinks/week had lower risk of having CAC 1–300 (OR:0.34) and CAC > 300 (OR:0.28) in multivariable logistic regression (with adjustment) yet in univariate comparison by chi-squared test never drinkers had lowest CACs > 0 prevalence, furthermore those with > 7 drinks/week had increased cIMT (increase of 0.096) but not a significant change in CACs, nevertheless authors suggested some J-shaped association for CACs to alcohol intake, while author notes limitations for possible residual confounding despite some adjustments, underreported alcohol intake and low number of participants with > 14 drinks/week.

Lee [148] discerned those with CAC > 0 had greater number of drinking days/week (β = 0.090, p < 0.05).

A cohort study by McClelland et al. [76] involving 6791 participants aged 45–84 with 2 to 4 years of follow-up, found no J-shaped association between alcohol consumption and CAC at baseline regardless of beverage type except consuming threshold > 20 g of beer/day and no relation between baseline, incidence and progression of CAC score with alcohol consumption unless consumes > 2 drinks/day or > 20 g of liquor/day, moreover, alcohol consumption > 2 drinks/day vs. never-drinkers associate with greater prevalence of HTN, higher SBP and DBP, and lower fibrinogen level.

Baek [149] recruiting 10,568 Korean KNHANES registered adults and 9586 Korean KOICA registered adults in cross-sectional design with deriving retrospective patient records, demonstrated compared to no use of alcohol and smoking in KHANES participants those with smoking (OR:2.37), alcohol use (OR:3.09) and concurrent use (OR:4.59) had greater risk of having high TyG (Triglyceride Glucose) index, consistently also for KOICA registry smoking (OR:1.33), alcohol use (OR:1.42) and concurrent use (OR:1.94) associated with greater risk of high-TyG, moreover, in KOICA registry no regular exercise also posed greater odds of high-TyG (OR:1.26).

Kimani [150] in cross-sectional design including only 1035 Japanese males presented 1 SD increase in alcohol intake (g/day) significantly associated with having 5‘th percentile AAC (Abdominal Aortic Calcification) vs having < 5‘th percentile score whereas this percentile comparison couldn‘t reach significance for CAC-s on both age-adjusted (AAC OR:1.45) and multivariable adjusted models (AAC OR:1.42), similarly in log-transformed calcification scores log[AAC + 1] rather than log[CAC + 1] could reach significant association with 1 SD increase of alcohol intake (g/day).

Pedrosa [151] in cross-sectional analysis recruiting 2433 ELSA-Brazil registered participants examining relations of CVD-risk factors with calcification of different vascular beds illustrated excessive alcohol intake (For Males ≥ 210 g/week, For Females ≥ 140 g/week) significantly associated with CAC > 0 vs CAC = 0 (14.0% vs 7.8%) but not with TAC > 0 (a non-zero Thoracic Aorta Calcification) vs TAC = 0; whereas in fully-adjusted models current smoking status posed significant non-zero calcification for both CACs (OR:1.67) and TACs (OR:2.16) besides for only CACs past-smoker status associated with non-zero score (OR:1.41), moreover, TACs remained similar with gender and ethnicity differences yet CACs significantly associated with male gender (Female OR:0.30, Male Reference) and white ethnicity compared to black ethnicity (OR:0.63 of White).

Yun [152] involving 24,681 Korean subjects (20696malesand3985females) in cross-sectional analysis illustrated facial flushing immediately after intake of 1 drink, which significantly associates with high acetaldehyde level and it‘s main cause of ALDH-2 enzyme deficiency or variability prevalently seen among East Asian, compared to non-flushers significantly associated with lower log-transformed alcohol consumption Log_2_[(alcohol intake(g) + 1)/8] as 1.6 vs 2.5, progressively lower share in higher alcohol intake categories (on 0, <8, 8–30, ≥30 g/day), lower daily alcohol intake (5.0 vs 12.1 g/day), lower AST, ALT and GGT liver enzyme levels and lower rate of CACs > 0; furthermore, in Instrumental Variables (IV) analysis of Alcohol flushing log-transformed Log2[(Alcohol + 1)/8] for males significantly associated with higher SBP (β = 1.251), DBP (β = 1.373), HDL-c (β = 1.476), TC (β = 2.293), CACs ≥ 1 (OR:1.11), CACs 1–100 (OR:1.10), CAC ≥ 101 (OR:1.21) and CACs in ordinal outcome (OR:1.11) along with lower BMI (β = 0.169), WC (β = 0.560), Log-Transformed TG (β = 0.053), Log-Transformed FPG (β = 0.013), Log-Transformed Insulin (β = 0.018) and Log-Transformed HOMA-IR (β = 0.031), and for females higher DBP (β = 1.309) and HDL-c (β = 1.865) besides lower Log-Transformed FPG (β = 0.010) and A1c(%) (β = 0.008) yet binary outcome for CACs ≥ 1 or incident-CAC couldn‘t reach significance; as a summary these analysis suggest facial flushing after alcohol intake could be used for alcohol consumption and health outcome assessments, and alcohol consumption significantly associated with CAC.

### 9.5 Metabolic health

#### 9.5.1 Obesity phenotypes

Sponholtz et al. [80] used several definitions to investigate associations of obesity and metabolic health within their variabilities in relation to their metabolic outcomes, CKD and CVD over 30 years long cohort. They defined metabolically unhealthy with using NCEP ATP-III Criteria (National Cholesterol Education Program Adult Treatment Panel 3;) [85] as presence of ≥ 2 conditions among high triglyceride (≥150 mg/dL or currently taking lipid-lowering medication), low HDL (<40 mg/dL for men, <50 mg/dL for women), high BP (SBP ≥ 130 mmHg, DBP ≥ 80 mmHg), and high blood glucose (≥100 mg/dL), while metabolic syndrome is defined as presence of ≥ 3 of those conditions; and across examination cycles variability of each MetS components were measured with VIM (Variability of Independent of Mean) as population mean of the variable is multiplied by the division of individual SD of the variable by individual mean of the variable [Population Mean*Individual SD/Individual Mean]a at which a is estimated through Ln(SD) regression with a*Ln(k*individual mean) noting k is constant; whereupon variabilities were defined as having VIM in the top quintile among observations pooled across all examination cycles for both BMI and metabolic health components with exception of blood pressure variabilities (for SBP, DBP). They observed that, compared to Metabolically Healthy Non-obese (MHNO), Metabolically Unhealthy Non-obese (MUNO) patients had greater probability of incident obesity (HR:1.88), incident diabetes mellitus (HR:4.01), incident hypertension (HR:1.74), incident CVD (HR:1.86), and incident CKD (HR:1.72). MHO (metabolically healthy obese) patients had been observed becoming metabolically unhealthy (HR:1.79; Ref:MHNO), and in predisposition to develop incident DM (HR:3.59; Ref:MHNO) and incident Hypertension (HR:2.11; Ref:MHNO). As greatest risk category among 4 obesity subphenotypes, MUO (Metabolically Unhealthy Obese) patients had greatest odds for incidence of DM (HR:8.39; Ref:MHNO), hypertension (HR:3.00; Ref:MHNO), CVD (HR:2.40; Ref:MHNO), and CKD (HR:2.43; Ref:MHNO). Among obese patients variable BMI variability had associated with higher incidence of Obesity (HR:2.63, Ref:SNO), metabolically Unhealthy (HR:1.67; Ref:SNO), DM (HR:1.58; Ref:SNO), and hypertension (HR:1.74; Ref:SNO). Similarly, among metabolically unhealthy patients both stable (HR:1.74; Ref:SNO) and variable metabolic health (HR:2.24; Ref:SNO) associated with higher incident CVD compared to SNO (Stable Non-obese).

On relation of obesity phenotypes and subclinical CVD measures in elderly, Roberson et al. [81] including 208 participants with mean age of 84 in cross-sectional analysis reported that across 4 obesity phenotypes there was no significant difference for median CACS (agatston unit), CACS severity categories (0, 1–100, 100–400, ≥400), cIMT, and hs-CRP > 3 mg/dL; as well as no significant relation for age, current smoking, meeting up with WHO recommended physical activity level, SBP, and DBP. However, obesity phenotypes showed significant differences for Framingham risk score, uric acid level (mg/dL), HbA1c, fasting blood glucose, BMI, at risk of elevated blood pressure (if SBP ≥ 130 mmHg and DBP ≥ 85 mmHg or on antihypertensive medication), and gender; nonetheless, stuy population has small size.

Roberson et al. [82], in their review study published at 2014, included 14 studies about investigating risk reducing behaviors. One of the study they mentioned, had found increased physical activity offset the increased CVD mortality seen in the Metabolically Healthy Obese (MHO) patients. Another 7 studies involved in their review, showed that MHO had higher level of self-reported physical activity as compared to MUHO (metabolically unhealthy obese) patients. They also reviewed that patients with BMI < 30 rather than BMI > 30 that insulin sensitive individuals had significantly higher mean daily fiber consumption as compared to insulin resistant patients.

Kowall B. et al. [84], which is a population-based cohort study including 1585 participants along 5-year follow-up, showed, where metabolically healthy normal weight patients (MHNW) are reference group in all comparisons across the study analysis, other 3 obesity phenotype groups had higher probability of coronary artery calcification as metabolically unhealthy obesity (MUO; PR:2.02), metabolically healthy obesity (MHO; PR:1.67), and metabolically unhealthy normal weight (MUNW, PR:1.62). In a logistic regression model they demonstrated, after adjusted for age, sex, smoking, physical activity, and education, MUO (HR:2.41) and MUNW (HR:2.08) but not MHO associated with annual CAC change. In multinomial logistic regression model, only MUO group had significant rapid CAC progression (which is exceeding expected according to extrapolation of baseline CAC score) at odds ratio of 1.55 (95% CI: 1.09–2.19).

#### 9.5.2 Analysis by MetS-Components

J. S Nam [153] including 9581 Korean adults with average follow-up of 4.2 years demonstrated across increasing baseline Atherogenic Index of Plasma (AIP) Log10[TG/HDL-c] tertiles from T1 to T3 significantly associated with increased Δ√Ln[CAC], annualized Δ√Ln[CAC] and higher rate of CAC-progression (either first non-zero CACs or increase of ≥ 2.5 units of Δ√Ln[CAC] over baseline) during the follow-up.

A. C Razavi [154] containing 574 participants over median follow-up period of 4.8 years presented in adjusted models maintaining zero-CACs significantly associated with younger age (each 10 years younger age OR:1.50, 95% CI:1.22–1.85), having traditional ASCVD risk < 7.5% (OR:1.51, 95% CI:1.08–2.11), hs-CTnT < 3 ng/mL (OR:1.55, 95% CI:1.01–2.38), absent thoracic calcification (OR:2.42, 95% CI:1.24–4.72), absent carotid plaque (OR:1.81, 95% CI:1.25–2.61) and 1‘st Quartile (lowest) vs 4‘th Q for MetS severity score determined from incorporating 5 MetS components (WC, SBP, HDL-c, TGs, FBG) adjusted with their relative weights by differential contributions in the latent factor based on correlation and interaction of each item with another to standardized Z-scores (OR:2.71, 95% CI:1.27–5.76) especially pronouncedly significant among participants with T2 DM (OR:5.96, 95% CI:1.27–28.07) yet only nonsignificant trend among participants with only metabolic syndrome (OR:2.25, 95 %CI:0.87–5.87, p = 0.09).

Scicali [155] including 711 participants retrospectively in cross-sectional analysis, whom are asymptomatic for any ASCVD and have prediabetes or newly indicated T2 DM status in control besides excluding partipants with severe renal disease, reported High TG/HDL vs Low TG/HDL by cut off of 2.45 significantly associated with higher CACs (29.15 vs 0.0), higher prevalence of non-zero CACs > 0 (64.5% vs 45%) and higher percentages of 2 (29.5% vs 21.5%) or 3 (32.7% vs 20.9%) different vascular sites (spanning coronary, carotid and femoral arteries) with atherosclerotic changes.

An analysis by Debarmore et al. [156] investigating possible associations between presence of CACs and 24-h blood pressure variability, which was assessed at 2 sessions of 24-h ambulatory blood pressure monitoring on intervals of visit 1 (day 1) to visit 2 (day 2) [ABPM-1] and visit 3 to 4 (day 9 to 10, respectively) [ABPM-2] with using WSD (Weighted Standard Deviation) formula summing weighted night and day time SD with number of measurements in assigned period and ARV (Average Real Variability) averaging absolute values of differences in consecutive BP measurements along with following exit visit of cardiac CT examination occurring days 10 to 17, recruiting 322 participants wıth high education and low risk of CVE from 12 primary care clinincs in central North Carolina demonstrated ambulatory BP variability in SBP or DBP had no significant association with CACs in either dichotomized CACs > 0 vs CACs = 0 by student‘s T-test or continuous CACs by linear regression for ABPM-2 besides no significant correlation with ARV or WSD measured SBP and DBP by Pearson‘s correlation coefficients, nevertheless highest reproducibility in results between ABPM-1 and ABPM-2 was observed for DBP (ICC:0.52, 95% CI:0.44–0.60) and lowest for SBP (ICC:0.39, 95% CI:0.30–0.49).

A cross-sectional study by Kutkiene et al. [157] included 213 participants aged 18–60 years of whom are 110 patients with severe hypercholesterolemia vs 103 patients with normal lipid profile in control group discerned no significant difference between severe hypercholesterolemia vs control groups on CACs percentiles (25th, 50th, 75th and 90th percentiles) among neither males (p = 0.706) and females (p = 0.272), moreover, ultrasonographical detected Achilles tendon xanthomas had no significant association with CACs percentiles.

#### 9.5.3 IR, DM and glycemic control

A cross-sectional observational study by De Block et al. [158] involving 118 adult T1DM patients aged 18–75 years with ≥ 5 years of diabetes duration in generally good health without significant symptomatic CVD, pregnancy, decreased GFR ≤ 30 mL/min/1.73 m2, to investigate role of VAT and adipocytokines in subclinical changes in CACs and Echocardiographic findings, demonstrated VAT ≥ 100 vs < 100, CACs ≥ 10 vs CACs < 10 and Diastolic dysfunction presence vs absence all significantly associated with greater age, duration of T1DM (in years), WC in men and lower eGDR (estimated-Glucose Disposal Rate) yet all had no significant association with HbA1c (%), HbA1c (mmol/mol), DBP, TC, TG and IL-6 levels, smoking and presence of microalbuminuria and polyneuropathy; only both VAT ≥ 100 vs < 100 and CACs ≥ 10 vs CACs < 10 significantly associated with higher BMI, WC in men, SBP, lipid-lowering and antihypertensive drugs use and rate of having metabolic syndrome yet both nonsignificant for adipocytokines of IL-6, TNF-α, adiponectin and leptin levels; moreover, only both VAT ≥ 100 vs < 100 and Diastolic dysfunction presence vs absence significantly associated with higher insulin level, CACs and rate of having CACs ≥ 10; furthermore, only VAT ≥ 100 vs < 100 significantly associated with lower HDL-c, eIS (estimated-Insulin sensitivity) and adiponectin levels besides higher TNF-α and Leptin levels; only VAT ≥ 100 vs < 100 significantly associated with higher levels of LDL-c and VAT-area size (cm2) and greater rates of having VAT ≥ 100 cm2, carotid artery plaques, wall-motion abnormalities and retinopathy; and last of their analysis LASSO (Least Absolute Shrinkage and Selection Operator Logistic Regression) revealed diabetes duration in years had highest predictive value for diastolic dysfunction among variables reported within this study, which may signify importance of earlier glycemic control.

Dayan et al. [120], along the mean follow-up period of 36.6 ± 3.3 months including 128 asymptomatic Turkish patients with median 10 years of type 2 diabetes history plus presenting additional at least one cardiovascular risk factor, revealed stratification by albuminuria severity as normoalbuminuric, microalbuminuria and macroalbuminuria had significant positive association with baseline HbA1c%, HDL-c, creatinine, TNF-α, IL-6 and use of insulin, moreover, age (β = 0.042), Ln(albuminuria in mg/day; β = 0.323) and uric acid (mg/dL; β = 0.129) could predicted Ln(CAC-score) at baseline in multiple regression model, and in multivariate Cox proportional hazards model Ln(CAC-score), Ln(albuminuria in mg/day), uric acid (mg/dL) and creatinine clearance could predicted CVE.

A cross sectional study by Kawasaki et al. [114], including 927 MESA-study participants from 6176 community recruits to probe relations of diabetic retinopathy, which was assessed by fundus photography and subsequently severity of diabetic retinopathy was determined by Airlie House classification system, with CACs, reported presence of diabetic retinopathy (DR) significantly associated with higher serum glucose (166.1 vs 148.5), HbA1c% (7.8 vs 7.0), categories of diabetes duration (for 3–10 years and ≥ 10 years), median diabetes duration, SBP, and greater rates of oral-diabetic medication and insulin use, moreover, in multivariate logistic regression analysis of covariates significant in univariate analysis compared to absent-DR participants in adjusted model-2 (for age, gender, ethnicity and study center + BMI, mean-ABP, Tc, TG, HDL-c, cigarette smoking, and CRP) present-DR (OR:1.72), CSME (Clinically significant macular edema; OR:2.86) and VTDR (Vision-threatening diabetic retinopathy; OR:2.97) significantly associated with high CACs > 400 yet in further adjusted model-3 (for model-2 + HbA1c, duration of diabetes and presence of diabetic nephropathy) and in fully-adjusted model-4 (model-3 + antihypertensive medication use, cholesterol-lowering medication and use of insulin or oral medications) only VTDR, which was defined as ≥ 51/80 Airlie House classification or severe-NPDR (Non-proliferative diabetic retinopathy) or proliferative diabetic retinopathy or CSME (clinically significant macular edema), remained significant for high CACs > 400 (OR:2.33 and OR:2.34, respectively).

An analysis of HNRS (Heinz Nixdorf Recall Study) prospective cohort study by Kowall et al. [159] including 3453 participants aged mean of 58.9 years with a median follow-up interval of 5.1 years for re-evaluation of CACs by ECG-gated EBCT to investigate effects of diabetes if it is indicated by participants‘ HbA1c data or already known diabetes as well controlled vs poorly controlled (≥7.0% vs < 7.0%), revealed incident CACs > 0 during follow-up period among participants with previously known diabetes was almost twice the patients without previously known diabetes significantly only in crude model (RR:1.9) by Poisson regression models, in multinomial logistic regression model 3 (fully adjusted for age, gender, BMI, smoking status, daily alcohol consumption, education level, SBP, DBP, use of antihypertensives, HDL-c, LDL-c, TGs, use of statins) only patients having previously diagnosed diabetes and poor glucose control (HbA1c ≥ 7.0) had significantly higher rate of rapid CACs progression over annual change of > 30% of baseline score (OR:2.3, 95% CI:1.4–23.7) compared to reference group of patients having no previous DM history and HbA1c < 5.7% yet only a nonsignificant trend of increased risk for participants with well controlled known diabetes history (HbA1c < 7.0%), similarly according to fully-adjusted linear regression model % change in geometric mean of PF5 (Progression Factor at 5‘th Year Examination), which is extrapolation of exponential model derived on age and gender specific CACs percentile at baseline to subject‘s percentile by the time elapsed since baseline, significantly associated with only patients with known poorly controlled diabetes (69.1%, [33.9–113.6]) compared to reference group; moreover, by multinomial logistic regression during inter-scan period absolute annual increase of > 100AU vs < 10AU significantly associated with both poorly-controlled (OR:10.0) and well-controlled (OR:4.0) known diabetes and patients without known diabetes remained similar, and annual absolute CACs change 10–99 vs < 10 significantly associated with only participants having known poorly controlled diabetes but with a weaker association (OR:4.1).

A serial evaluation of 1637 asymptomatic adult patients with diabetes mellitus over median inter-scan period of 3 years by K.-B. Won et al. [99], revealed optimal glycemic control (OGC) group defined as HbA1C of < 7.0% compared to non-OGC group as HbA1c ≥ 7.0% significantly associated with lower annual increase in CAC score, lower prevalence of CAC progression as square-root of difference in CAC within inter-scan period becoming ≥ 2.5, and lower risk of progressing CAC in follow-up period exceeds CAC ≥ 200 and CAC ≥ 300 scores.

A longitudinal study by Saremi et al. [160] involving 197 participants, who had ever hypoglycemic symptoms or PG < 70 mg/dL and of 97 participants had at least 1 severe hypoglycemia since last visit with either 74 documented BG < 50 mg/dL or 23 severe episodes, within mean CACs-interscan follow-up period of 4.6 ± 0.6 years delving relation between hypoglycemia and square root transformed volumetric CACs progression (Δ√(Volumetric CACs)) and testing whether the relation is modified by the intensity of glycemic control, revealed participants with serious hypoglycemia vs without on baseline had higher rate of intensive treatment, prior hypoglycemia and basal insulin use, longer diabetes duration, higher HDL-c, Creatinine and CACs levels, lower HbA1c, C-peptide, SBP, DBP and TG levels, and lower sulfonylurea use; in stratification of participants into treatment arms of standard and intensive for both overall cohort and intensive treatment group no significant difference in CACs-progression between patients with serious hypoglycemia and without, moreover, in model adjusted for the variables selected from stepwise variable selection model (i.e Baseline CACs, LpPLA_2_, Albumin-to-creatinine ratio, age, duration of diabetes, ethnicity, prior hypoglycemia) among standard treatment group having serious hypoglycemia persistently significantly associated with progression of CACs yet among intensive treatment group having severe hypoglycemia had no significant difference in progression of CAC than without; furthermore, in sensitivity analyses number of serious hypoglycemic episodes as 0, 1–5 and > 5 had no significant difference in median- volumetric CACs progression among intensive treatment group yet a significant trend among standard treatment group and on categories of mean HbA1c > 7.5% and HbA1c ≤ 7.5% serious hypoglycemia had significant association with progression of CAC among HbA1c > 7.5% but not HbA1c ≤ 7.5%, so severe hyperglycemia or insufficiently/poorly controlled diabetes could account and be harbinger of adverse effects of serious hypoglycemia in progression of CAC.

A longitudinal study by Cho et al. [161] including 1145 participants aged mean 54.2 ± 7.6 years with mean inter-scan follow-up period of 3 years to compare HOMA-IR, TyG (Ln[TG*FPG/2]), TyG-BMI (TyG*BMI) and TyG-WC (TyG*WC) indexes for IR in estimation of CACs-progression (SQRT ≥ 2.5 units), which was defined as “square root-transformed difference” between baseline and final CACs assessment ≥ 2.5 units, reported CACs-progressor vs non-progressor significantly associated with higher age, WC, SBP, DBP, serum levels of FPG, HbA1c, uric acid, AST, ALT and GGT, index levels of TyG, TyG-BMI and TyG-WC, CACs at baseline and final assessment, prevalence of diabetes and hypertension, and higher rates of male gender, current smoking and moderate-drinking; moreover, proportions of CACs-progressors had significant linear trend with Quartile scores of IR-indexes (Q1,2,3,4) of HOMA-IR (p = 0.031), TyG (p = 0.007), TyG-BMI (p < 0.001) and TyG-WC (p < 0.001) with graded association across quartiles only for HOMA-IR and TyG-WC and annualized difference of SQRT-CACs also significantly associated with these 3 IR-index parameters excepts HOMA-IR besides graded associations at obesity related indices of TyG-BMI and TyG-WC; in addition, among IR-indexes in fully-adjusted model (for age, gender, SBP, LDL-c, HDL-c, smoking, drinking, exercise-habits, baseline CACs and follow-up interval) only TyG-WC could significantly associated with CAC-progression for both Q4vsQ1 (OR:1.66) and Q3vsQ1 (OR:1.64) and TyG-BMI could have significant association for only Q4vsQ1; furthermore, in ROC-curve analysis for the prediction performance of IR-indexes TyG-WC had highest AUC(0.600) and TyG-WC had significantly greater achievement compared to HOMA-IR (AUC = 0.543) and TyG (AUC = 0.557) but not with TyG-BMI (AUC = 0.583).

#### 9.5.4 Cardiac structure and function

A cross-sectional analysis of CV-health check-up for healthy adult population by Kim [162] including 565 Korean subjects aged mean 59.5 years to examine associations of exercise capacity, CACs, Cardiac structure and function along with components of metabolic syndrome defined by either ATP-3 or ATP-3BMI 25 criterion to categorise participants according to number of presenting components as zero-component (group 1), 1–2 out of 5 components (group 2) and ≥ 3 out of 5 components (group 3), demonstrated across MetS categories HRR (Heart rate recovery as the difference from peak HR during exercise to HR at 1 min after cessation of exercise, in BPM) by ETT (Exercise tolerance test) inversely but LADA-CACs, total CACs, Calcium volume score and echocardiography defined IVS (Interventricular septum thickness, mm) and PW (Posterior wall thickness, mm) directly associated by one-way ANOVA, group 2–3 vs group 1 ETT measured exercise duration (s) and METs decreased yet LCXA-CACs, RCA-CACs and echocardiography defined LVEDD (Left ventricular end-diastolic diameter, mm), A (Peak late-diastolic transmitral-flow, cm/sec) and E/A (ratio of peak early-diastolic to late-diastolic transmitral flow) increased by one-way ANOVA; according to multi-variable logistic regression group 3 vs 1 associated with lower HRR (bpm, aOR:0.90, 95% CI:0.913–0.969) and E/A (aOR:0.108, 95% CI:0.035–0.336), higher total and calcium volume CACs (aOR:3.003, 95% CI:1.636–5.513); according to pearson‘s correlation analysis exercise duration with RCA-CACs (r = -0.1), total and calcium volume CACs (r = -0.1), A(cm/s, r = -0.27) and E/A (r = 0.18), METs with E (cm/sec, r = -0.1) and A(cm/sec, r = -0.17), HRR with IVS(mm, r = -0.11), PW (mm, r = -0.16), E(cm/sec, r = 0.09) and E/A (r = 0.12), A with LMA-CACs (r = 0.08), LADA-CACs (r = 0.1) and LCXA-CACs (r = 0.08), and LADA-CACs with IVS (r = -0.12) and PW (r = 0.09) significantly correlated; nevertheless LVEF% (Left ventricular ejection fraction) also LVEDD and LVESD had no significant association with any of the parameters measured by noted 3 tests (Echocardiography, ETT and CACs) by Pearson correlation analysis this may suggest systolic functions unlike diastole could be partly preserved in MetS and SCVD.

#### 9.5.5 Biomarkers

Study by Tzoulaki [163] et al. examining serum metabolites via Standard 1D 1H-NMR spectroscopy with spectrums of water suppression (NOESY-Presat Sequence) and T2-Edited Spectrum (using CPMG sequence) included 3867 participants with MESA-registry for discovery spectroscopy analysis (at which significance of p value 1.3*10–14 to 1.0*10–6) and 3569 participants with LOLIPOP-registry (n = 1917) and Rotterdam-registry (n = 1652) in 6 batches (2 1H-NMR studies at each cohort); the study detected metabolites through SRV (Statistical Recoupling of Variables) analysis of 1H-NMR spectrum features identifying maximum of 3 clusters of consecutive resonance feature with a correlation of r ≥ 0.9 then which discerned cluster equivalent signal peaks were compared with available databases on human serum and metabolite components to the metabolite assignments with levels of signal overlap and it‘s confidence for annotating metabolites; following described study design authors observed 74 NMR spectral signals associated with transformed-CACs Ln[CAC + 1] in MESA-discovery cohort (p < 1.8*10–5) in which 41 spectral signals were replicated in Rotterdam and LOLIPOP replication cohorts (p < 0.05) of 19 signals became annotated metabolites; the study reported metabolites directly associated with Ln[CAC + 1] are Alanine (mitochondrial metabolism, TCA cycle, aerobic energy metabolism), Glycine (1-carbon metabolism, Amino acid), Methionine (Sulphur Metabolism, Amino acid), 4 Polyol and Carbohydrate Metabolism metabolites (D-Glucose, 1,5-Anhydrosorbitol[insulin resistance], Mannose[prediabetes, T2DM and pro-inflammatory associations, lipoprotein glycation], Myoinositol), Histidine (Muscle Metabolism), Acetyl Glycoproteins (secreted in pro-inflammatory state), Glycerol (Lipid and Fatty Acid Related Metabolism) and Acetaminophen-Glucoronide, and 9 metabolites inversely associated with Ln[CAC + 1] are 2 metabolites involve in regulating [Glutathione] concentration (Glutamate and 5-oxoproline[Urea Cycle Metabolism]), Glutamine (Mitochondrial Metabolism), N,N-Dimethylglycine (1–carbonmetabolism), Lysine (Mitochondrial Metabolism), Phenylalanine (aromatic Amino acid), 3-hydroxybutyrate (Mitochondrial Metabolism), Citrate (TCA cycle) and Albumin; moreover, across 3 cohorts based on permutation of spectral features in random allocation of CACs (Ln[CACs + 1]) outcomes to each study participants as MWAS (Metabolome Wide Association Studies) below Metabolome Wide Significance Level (MWASL) threshold (p = 2.4*10–9 to 1.1*10–5) with Ln[CACs + 1] mannose, alanine and acetaminophen-glucoronide strongly associated yet glutamate and histidine had strong inverse associations besides other significant metabolites also remained significant but by 50% weaker association; furthermore, lipoprotein subcategories of total plasma cholesterol, total plasma apolipoprotein B and apolipoprotein B within total plasma LDL significantly associated with Ln[CACs + 1].

A follow-up study by Diederichsen et al. [164] in population size of 1221 male participants aged mean 55.39 ± 5.01 with 5 years of follow-up to investigate role of 15 biomarkers including calcium-phosphate and lipid metabolism, inflammation, kidney function and myocardial necrosis in CACs incidence and progression, reported among participants with CACs = 0 baseline biomarkers of TG, LDL-c, TC, CRP, cTnI, Creatinine, Cystatin-C and urate levels significantly associated with incidence of CACs > 0 during follow-up period but not with CACs-progression (>15% of annual increase in absolute score exceeding ≥ 20 AU) and similarly CV-risk factors of male-gender, age, BMI, HTN and active smoking also posed significant risk for CACs > 0 incidence, moreover, other biomarkers of OPG (osteoprotegrin), Calcium, Phosphate, CPP (Calcium-Phosphate Product), Vitamin-D3, PTH, HDL, suPAR (Soluble urokinase-type Plasminogen Activator-Receptor), eGFR and eGFR had no significant association with either CACs > 0 incidence or CACs-progression during follow-up period, nonetheless, as a CV-risk factors only dyslipidemia among investigated conventional CVD-risk factors and biomarkers could have significant association with CACs-progression, furthermore, in multivariate general linear models among participants with zero-CACs baseline exceptionally Calcium, CPP, LDL-c and TC significantly associated with absolute CACs change while among participants with non-zero CACs only Phosphate and CPP significantly associated; and these results suggest lipid-lowering treatment of statin used in dyslipidemia may not be effective in decelerating CACs-progression since LDL-c and TC had no significant association with CACs change while plaque-stabilizing effect of statin causing densely calcified plaques.

A follow-up study by Saremi et al. [165] involving 411 patients aged mean 58 ± 8 years along mean follow-up period of 10 years with valid 398 US-cIMT, 353 CT-CAC and 345 CT-AAC examinations to investigate, if existent, roles of 7 biomarkers, which were measured by liquid chromatography-mass spectrometry, including 5 Dicarbonyl-derived AGE (Advanced Glycation End-products) molecules of Nε-carboxyethyllysine (CEL), glyoxal hydroimidazolone (G-H1), methylglyoxal hydroimidazolone (MG-H1) and 3-deoxyglucosone hydroimidazolone (3DG-H), and 2 OxP (Oxidative Stress Products) molecules of methionine sulfoxide (MetSO) and 2-aminoaipic acid (2-AAA) in changes of SCVD measurements within follow-up period, reported baseline quartile 4 vs lower quartiles of G-H1, 2-AAA, MG-H1, 3DG-H and CEL significantly associated with higher CACs, furthermore, in multi-variable linear regression model (adjusted for age, duration of diabetes, prior CVD, history of hypertension, pack-years of smoking, GFR, HbA1c, HDL-c and Triglycerides) dichotomous variables of G-H1 (Q4 vs Q1-3 combined; β = 5.53 ± 2.29) and 2-AAA (Q4 vs Q1-3 combined; β = 6.84 ± 2.21) and continuous variable of 2-AAA (β = 6.08 ± 2.74) significantly and robustly associated with CAC, moreover, continuous variable of CEL strongly and significantly associated with AAC (β = 13.77 ± 5.63) and G-H1 both continuous and dichotomous (Q4 vs Q1-3) forms significantly but weakly associated with c-IMT (β = 0.09 and β = 0.06, respectively); in addition, stratification of participants by combined scores of 2-AAA and G-H1 into 3 categories as from both scores below Q4, only one score within Q4 to both scores within Q4 respectively showed significant trend of higher CACs change along follow-up period.

#### 9.5.6 Plaque burden and stability

Y. H. Chung [166] including 2019 participants (M:1518 (75.2%), F:501 (24.8%)) in cross-sectional analysis on relation between CACs and Lp(a), which was noted with its composition of apoB-100 and apolipoprotein(a) bounded in disulfide bond, it‘s small-particle size allowing freely crossing endothelium, similarity of apo(a) with plasminogen interfering it‘s antithrombotic actions and a moiety in Lp(a) closely resembling LDL; revealed among males aged ≥ 45 years (1313 participants, 86.4% of Males) those with CACs > 0 vs CACs = 0 had higher Lp(a) level by chi-square test (16.74 vs 13.97) and Lp(a) level significantly associated with having CACs > 0 by both univariable (OR:1.008, 95% CI:1.003–1.014) and multivariable models (OR:1.010, 95% CI:1.004–1.016), however, among males aged < 45 years and females aged either < 55 years or ≥ 55 years Lp(a) levels couldn‘t reach significant trend with having CACs > 0 by univariable and multivariable analyses, nevertheless, those with Lp(a) > 50 mg/dL vs Lp(a) 15–30 had significantly higher CACs by Kruskal-Wallis test.

A cohort study by Garg et al. [167] analyzing 5456 participants of MESA study with median follow-up period of 10.2 years to examine relation of Lp-PLA_2_, which as a calcium-independent enzyme highly expressed at macrophages infiltrating plaque hydrolyses oxi-LDL and it‘s activity destabilize plaque into vulnerable and rupture-prone plaque, on baseline mass and activity determinations with incidence of CVE within cohort besides evaluating stratification of the relations by baseline subclinical atherosclerosis, reported both Lp-PLA_2_ mass (ng/mL) and activity (nmol/min/mL) indexes have significantly greater level among incident vs non-incident CVD, CHD and Hard-CHD, moreover, in adjusted model (for age, gender, ethnicity, BMI, DM, Smoking status, High-school education, SBP, use of anti-hypertensive medication use, TC, HDL-c, use of lipid lowering medication, CRP, +maximal-cIMT, maximal ic-IMT and CACs > 0) increase of per 1-SD increment Lp-PLA_2_ mass (42 ng/mL) and Lp-PLA_2_ activity (36 nmol/min/mL) significantly associated with CVD (OR:1.10, OR:1.11 [p = 0.06, borderline]), CHD (OR:1.14, OR:1.17) and Hard-CHD (OR:1.31, OR:1.22), respectively; furthermore, in adjusted model (excepts for SCVD-determinations) stratified by the SCVD-status only Lp-PLA_2_ mass index (per 1-SD increment) could persisted significant association with CVD-events (HR:1.13) among only presenting-SCVD yet interaction of Lp-PLA_2_ mass index with SCVD-status was nonsignificant; and as a summary these findings could suggest Lp-PLA_2_ and SCVD have independent mechanism for their associations for CVD-incidence.

A cohort study by O. Dzaye et al. [104] including analysis of 54,678 patients aged mean 54.2 years, reported combining CAC (A_X_) in traditional score groups 0 to 3 stratified at each CAC 100 from CAC = 0 reference point to CAC ≥ 300 and number of vessels with CAC (N_Y_) in range of 1 to 4 cumulatively counting extension in LM (Left Main), LAD (Left Anterior Descending), LCX (Left Circumflex) and RCA (Right Coronary Artery) to recategorize in A_X_/N_Y_ scoring compared to CAC score groups alone could significantly improved ROC-curve reclassification of mortality risk caused by CHD, CVD and all-cause from 0.785, 0.754 and 0.696 to 0.795, 0.762 and 0.700 respectively, as such compared to reference group of A0 with increasing severity of A_X_N_Y_ categories associated with graded increase in CHD, CVD and all-cause mortality, moreover, in adjusted model (of age, gender, race, hypertension, hyperlipidemia, current smoking, family history of CHD, and DM-comorbidity) A_3_N_4_ group compared to reference group 5.9, 4.0 and 2.5 times higher risk for CHD, CVD and all-cause mortality respectively.

#### 9.5.7 Lipid Metabolism

V. S. Nunes [168] including 344 males, whom are healthy, non-obese, non-diabetic and no asymptomatic for CAD, with ELSA-Brasil study registry in cross-sectional design (gathering plasma samples, CACs and CCA-IMTs measurements, vital parameters, anthropometric measurements, dietary patterns with food frequency questionnaire and patterns of alcohol and cigarette use) illustrated 3‘rd vs 1‘st tertile both cholesterol synthesis marker desmosterol (OR:3.241, 95% CI:[1,700–6.179]; rather than lathosterol) and cholesterol absorption marker campesterol (OR:1.858, 95% CI:[1.020–3.387]; rather than sitosterol) significantly associated with having a CACs > 0, moreover, total cholesterol was more relevant with having CACs > 0 vs 0 other than having CCA-IMT ≥ 75% vs < 75% percentile categories and both desmosterol and campesterol had no significant association with CCA-IMT category, however, SBP was prevalent and more specific to CCA-IMT categories.

Ceponiene [169] including 3305 participants to analysis with mean follow-up of 9.6 ± 0.6 years and testing lipoprotein in baseline plasma samples with gas phase electrophoresis technique (Ion Mobility Method) for quantifying size based lipoprotein subfractions, illustrated for absolute annual CACs change non-HDL-p, very small LDL-p (3b to 4c, 18.0–20.82 nm diameter), small LDL-p (3a, 20.82 to 21.41 nm) and total LDL-p positively correlated and large LDL-p (1 to 2a, 20.0 to 23.3 nm) besides large HDL-p subfractions (10.4 to 14.5 nm) inversely correlated, however, after adjustment for conventional risk factors than additional for conventional lipids of LDL-c, HDL-c and TG positive correlation of very small LDL (3b to 4c, β = 0.01 per nmol/L) and inverse correlation of large LDL (specifically 2a, β = -0.03 per nmol/L) and medium LDL (2b, β = -0.02 per nmol/L) remained significant.

A study by Cao et al. [170] including 4623 participants with MESA-registry along 8.8 years of median follow-up and reporting ApoB level discordance with LDL-c and non-HDL-c, which is ApoB levels more/less than expected or residuals from linear regression models in association with LDL-c or HDL-c defined as apo-B discordant-low (<25‘th percentile residual), concordant (25‘th-75‘th percentile residual) and discordant-high (>75‘th percentile residual), in relation to ASCVD marker of CAC, demonstrated baseline prevalence of CACs > 0 for minimally adjusted model (Age, gender, race/ethnicity, smoking status and Hypertension) discordant high Apo-B relative to LDL-c directly and discordant low Apo-B relative to non-HDL-c inversely associated yet for fully adjusted model (Minimally adjusted model + BMI, Diabetes status and Ln[TG]) only discordant low Apo-B to non-HDL-c significantly besides inversely associated, rate of incident CACs > 0 within follow-up among with CACs = 0 at baseline for minimally adjusted model discordant low Apo-B to LDL-c inversely and discordant high Apo-B to non-HDL-c directly significantly associated yet in fully-adjustment model any discordant Apo-B to either LDL-c or HDL-c remained similar, CACs progression of > 75‘th percentile (cut-point of change 16.44 AU/year) significantly associated with discordant high Apo-B to non-HDL-c in both minimally-adjusted and fully-adjusted models; at all 3 aforementioned outcomes of CACs across tertiles of individual measures of Apo-B, LDL-c and Non-HDL-c significantly associated with ASCVD.

A cohort study by Miname et al. [223] including 206 asymptomatic participants with heterozygotes Familial Hypercholesterolemia (FH) receiving lipid-lowering therapy aged mean 45 ± 14 years on median follow-up period of 3.7 years to examine role of CACs in ASCVD-event risk prediction among participants with molecular defects in FH-related genes, reported across CACs categories of 0, 1–100 and >100 HTN, FH of premature CHD, Corneal Arcus, baseline total-cholesterol, LDL-c, TG, cholesterol-years score and statin use along with lipid-lowering therapy duration gradually increased, moreover, in univariate COX-regression analysis male gender (HR:3.6), FH of premature CHD (HR:3.4), Corneal Arcus (4.6), HDL-c (HR:0.9) and Log(CACs + 1) (HR:3.8) significantly associated with MACE, however, in multivariate COX regression model development by significant findings of univariate analysis only Log(CACs + 1) remained significant (HR:3.33) and with further adjustment for the type of LDL-R mutation defect LDL-c and Log-transformed CACs persisted significant association with MACE, furthermore, NNT_5_ values for additional PCSK9-I (with assumed RRR of 20%) to standard lipid-lowering therapy were presented for CACs 1–100 and CACs > 100 as respectively 38 and 23.

A cross sectional study by Djekic et al. [171] including 70 patients, who had diagnostic coronary angiogram within 12 months prior of acceptance to study without significant coronary artery stenosis (>50%) and CVE-history, to examine roles of different lipid classes (4 main classes of Glycerolipids, Glycero-phospholipids, Sphingolipids and sterol lipid) in CACs incidence and severity (No-CACs:0 or NCC, Mild-CACs:1–250 or MCC, Severe-CACs:>250 or SCC), identified lipids in chromatographic separation subsequently analyzed in electro-spray ionization (ESI) of mass-spectrometer in positive (ESI + ) polarization and negative (ESI-) polarization; reported by binary-logistic regression models accepting FDR (False-Discovery Rate) with Benjamin-Hochberg correction of α = 0.10 SCC vs NCC categories significantly associated with increased PC(16:0/20:4, Phosphatidylcholine, Glycero-phospholipids) and decreased PC(18:2/18:2), PC(36:3), PE(20:0/18:2, Phosphatidylethanolamine, Glycero-phospholipids) and SM(d:34:1, Sphingomyelin, Sphingolipids) while TG (Triacylglycerol) subclass lipids (higher levels of lipids of Cluster-3 allocation among n = 6 centroids by K-means clustering) and lower levels of DG(36:2, Diacylglycerol), DG(36:3), SM(d:34:1) and Cer(d18:1/24:0) trends couldn‘t reach significance, SCC vs MCC significantly associated with higher levels of PC(16:0/20:4) and lower levels of PC(18:0/18:0), and MCC vs NCC had no significant association with any of the 4 main lipid classes, Glycerolipids (with TG and DG subclasses), LPC (Lysophosphatidylcholine among Glycerophospholipids), Cer (Ceramide among Sphingolipids) and CE (Cholesterol ester among sterol lipid) had no significant association with any of the binary comparisons; moreover, as noted by authors PC making up cellular membrane interacts with PLA2 secreted by inflammatory cells and yields LPC, arachidonic acids, prostaglandins and thromboxane besides modifies LDL-c so as to underlie association of inflammation and CACs. In addition, PE involves in autophagy (autophagosome structure as involving in lipidation of LC3/Atg8) degrades autophagosomal cargo materials such as cellular debris, pathogens, macromolecules, toxic molecules to impede cellular senescence and formation of ROS [206], and SM has homeostatic functions in maintenance of vascular integrity, immune cell trafficking and protective role in complex with HDL-c for endothelial barrier functions [207].

#### 9.5.8 Bone Metabolism

Cahalane et al. [172] recruiting 64 endarterectomy patients (including 36 carotid ,31 lower extremity and 3 multiple endarterectomy procedures) with preoperatively collected circulating biomarkers quantified by ELISA Test kit, presented local calcification inhibitor dephosphoryalted uncarboxylated isoform of Matrix γ-Carboxy Glutamate (dp-ucMGP) weakly inversely correlated with CAC-Density score (r = -0.338, p = 0.047, valid n = 38 analysis participants) and in carotid endarterectomy patients (n = 19) moderately inversely correlated (r = -0.592, p = 0.008), moreover, proportion of uncarboxylated osteocalcin as inactive form of bone formation marker of osteocalcin to total osteocalcin (%ucOC) weakly inversely correlated with CACs (r = -0.335, p = 0.040, n = 38) yet systemic calcification inhibitor Fetuin-A had no significant association with CACs, CAC-Volume and CAC-Density scores.

#### 9.5.9 Endocrine actions

A cross-sectional analysis of longitudinal ELSA-Brasil study protocol by De Miranda et al. [173] selecting 3836 participants (of which are 3551 euthyroid, 239 subclinical hypothyroidism [SCHypo] and 46 subclinical hyperthyroidism [SCHyper] patients) aged median 49 years excluding patients with overt thyroid disorders and patients receiving thyroid disorder treatments to observe relations between TSH levels with noting categories of subclinical thyroid disorders (SCHypo[median: 5.01 mIU/L] and SCHyper[median:0.26mIU/L]) and euthyroidism (median:1.54 mIU/L) and CACs, demonstrated across quintiles of TSH 1 to 5 proportion of women BMI, TG and HOMA-IR increased and GFR and rate of current smokers decreased; compared to reference 3‘rd Q. of TSH (1.39–1.85 mIU/L) 1‘st Q. (OR:1.57, 1.05–2.35) in fully-adjusted model of 3 (for Age, gender, race, HTN, DM, Dyslipidemia, smoking, BMI, HDL-c, TG, GFR, hs-CRP and HOMA-IR) but not other quantiles significantly associated with CACs > 100 in whole samples and this association was modified by gender to U-shaped association among both gender groups as for males 1‘st Q. (OR:1.72) and 4‘th Q (OR:1.73) and for females 1‘st Q. (OR:3.31) and 5‘th Q. (OR:3.29) posed significant risk, however, after limiting analysis to participants within euthyroid reference range of TSH (0.4 to 4 mIU/L) compared to reference 3‘rd Quartile in adjusted model-3 U-shaped associations were lost for overall euthyroid participants and gender-stratified subgroups and only 1‘st Q vs 3‘rd Q remained significant for CACs > 100 (Overall-OR:1.70, Male-OR:1.74 and Female-OR:2.83), nonetheless, in analysis combining 2‘nd to 4‘th Quartiles as reference group female patients on contrary to males had significant U-shaped association yet after limiting participants to euthyroid reference range only 1‘st Q subjects had significant association with CACs > 100, and these analysis unfold role of suppressed TSH in advanced CACs > 100 development.

A cross-sectional study by Lee et al. [174] including generally healthy 195 male subjects (as 98 Caucasians and 97 Japanese Americans) aged 40 to 49 years without CVD history from population-based records, analyzing relation of Vit-D Deficiency vs Sufficiency (25(OH)D < 20 ng/mL vs ≥ 20 ng/mL) with SCVD determined by CACs (as CACs ≥ 10 vs CACs < 10), reported Vitamin-D deficiency significantly associated with greater risk of having coronary artery calcification in fully-adjusted model (of age, BMI, pack-years of smoking, drinking, CRP, TG, HTN, DM and serum levels of marine n-3 fatty acid including EPA, DHA and DPA).

A cross-sectional study by Posadas-Sanchez et al. [175] including 1276 participants aged mean 54 ± 9 (SD) years without FH of CHD and personal history of CVD from 1500 control-group participants of GEA-study (Genetics of Atherosclerotic Disease) based on Mexican-mestizo population, examining relation of serum Magnesium, which was noted with calcium antagonism regulating BP, peripheral circulation and vasomotor tone and its role in ATP-transfer reactions, insulin release by pancreatic β-cells and second messenger for insulin action, with coronary calcification (CACs > 0), reported across serum Mg Quartile scores Q1-to-Q4 inversely associated with SBP, FPG, HOMA-IR and hs-CRP, lower prevalence of HTN, T2-DM, IR, MetS and CACs > 0, and directly associated with TC, LDL-c, Apolipoprotein-B and proportion of Menopausal women; moreover, in multivariate logistic regression analysis (adjusted for age, gender, education, smoking status, elevated abdominal-VAT, fasting-insulin, FPG, physical activity, alcohol consumption, menopausal status for women, FH of T2-DM, T2-DM and diuretic use) Q4 vs Q1 serum magnesium level significantly associated with lower odds of HTN (OR:0.52), T2-DM (OR:0.31) and CACs > 0 (OR:0.58), and again per 1-SD (0.17 mg/dL) increment of serum magnesium concentration significantly associated with coronary artery calcification (CACs > 0).

A cross-sectional study by Gronhoj et al. [176] involving 1088 study participants without previous CVD-event and defined DM randomly selected from Danish national civil registry equally at 50 and 60 years of age to examine relations of calcium-phosphate metabolic panel with presence and severity of coronary artery calcification, reported in univariate ordinal logistic regression analyses serum calcium (per 0.1 mmol/l increase) increase 16% odds of higher CACs-category (defined as 0, 1–99, 100–399 and ≥ 400) and 25(OH)-Vitamin D (per 1 mmol/l) decrease 1% odds of higher CACs-category yet serum Phosphate, CPP (Calcium Phosphate Product) and PTH had no significant association with CAC; moreover, in multivariable LR-analyses (adjusted for age, gender, smoking, hypercholesterolemia, hypertension and Family history of CVD) among overall study serum calcium, phosphate, PTH and 25(OH)-Vitamin D serum concentrations had no significant association with CAC-severity categories yet only among male-participants only serum Ca concentration (per 0.1 mmol/l) significantly positively associated with CACs-severity categories (OR:1.30, 95 %CI:1.04–1.64).

#### 9.5.10 Inflammatory actions

A study by Harada et al. [177] involving 3753 participants from Sao Paulo in cross-sectional analysis and examining acute phase glycoproteins by NMR with GlycA signal (glycan composed of N-acetyl moiety) demonstrated both GlycA 4‘th Quartile vs 1‘st quartile and GlycA (µmol/L) significantly associated with having a CACs > 0 among metabolic syndrome-free patients yet these associations attenuated after adjustment for all metabolic syndrome characteristics, similarly for hs-CRP, uric acid and composite inflammation score (derived from combination of GlycA, Ln[hs-CRP] and Uric Acids in either arithmetic mean as continuous varible or summing assigned category point as 1 score point for each biomarker if it is greater than it‘s median value) association with having a CACs > 0 attenuated in adjustment for metabolic syndrome components, moreover, author highlighted association of inflammation with CAC in low cardiometabolic risk participants relating to low-grade chronic inflammation mainly mediated by insulin-resistant intra-abdominal visceral adipose tissue, which secretes 1/3 of circulating IL-6, and in stratified analysis of CACs only GlycA had significant standardized β value for participants with lower waist circumference (Males < 94 cm and Females < 80 cm) and those without metabolic syndrome.

A study by Ong et al. [178] including 5788 participants over median follow-up of 14 years reported FGF-21, which was noted as a novel biomarker in high risk CVD with anti-inflammatory, anti-oxidative and anti-apoptotic properties, significantly associated with older age, current smoking with high pack-years, less-physical activity, obesity, lower family income, higher resting heart rate, insulin resistance, having diabetes or hypertension, lower eGFR, using lipid lowering therapy, higher TGs, CRP, Fibrinogen and IL-6 levels; moreover, FGF-21 level had graded significant response in CACs at baseline and consistently association of each 1 SD of FGF-21 level with CACs remained significant for adjustment of age, gender, race/ethnicity and family income in model 1, however, for further adjustments in model 2 including aforementioned factors associated with FGF-21 (excepts CRP, Fibrinogen and IL-6) and again in further fully-adjusted model 3 (model 2 + CRP, Fibrinogen and IL-6); furthermore, non-linearly for FGF-21 ≥ 80.0 mg/dL FGF-21 level to CVD endpoints significantly associated with hard CVD in model 1 (HR:1.34, 95% CI:1.12–1.60) and borderline significance in model 2 (HR:1.20, 95% CI:1.00–1.45) besides hard CHD in model 1 (HR:1.46, 95% CI:1.19–1.80) and model 2 (HR:1.26, p = 0.045) yet in model 3 no significant association was observed for both end-points.

A follow-up study along an average of 10.2 years by Goldwater et al. [179] including 930 adults with MESA-registry demonstrated IL-10, which was noted with anti-inflammatory and anti-atherosclerotic properties and hypothesized to be secreted in response to pro-inflammatory IL-6 and TNF-α as a biomarker of proinflammatory settings pertaining adverse cardiovascular events risk, weakly correlated with IL-6 (r = 0.086, p < 0.01) and TNF-α (r = 0.097, p < 0.01) yet without correlation with hs-CRP, moreover, IL-10 level posed no significant risk for major adverse CV-events by Cox-Proportional hazard models at each 1 SD increase in Ln[IL-10] level (HR:1.26, p = 0.09) in both unadjusted and adjusted models and Kaplan-Meier time-to-event analysis across it‘s quartiles yet pro-inflammatory cytokine IL-6 posed significant risk for adverse CVE (HR:1.70, p < 0.01); furthermore, IL-10 level had no significant association with CACs and prevalence of having CACs > 0 , but with having CACs > 0 significantly associated with pro-inflammatory cytokines of both IL-6 (Prevalence RR:1.2) and TNF-α (Prevalence RR:1.19).

A follow-up study along 6.5 years by Diederichsen et al. [180] including 1179 subjects of with almost equal distribution for gender (618 Female and 561 Male participants) and age groups of 50-years (578participants) and 60 years (601participants) at baseline into analysis examining role of inflammatory biomarkers of CRP and suPAR (Soluble urokinase Plasminogen Activator Receptor), which is on immune cells and cleaved in inflammatory response than uPAR is released, illustrated by Cox-regression analysis adjusted for traditional CVE-risk factors and CACs (categorically and continuously) with CVE both suPAR (HR:1.20, 95% CI:1.04–1.38 regardless of continuous CACs) and CRP (HR:1.03, 95% CI:1.003–1.05 continuous CACs adjustment) significantly associated, however, stratification for age and gender groups showed differential results on which association of suPAR with CVE by Cox-regression remained significant only for Females and those with 60-years of age likewise CRP remained significant for age groups only those of 60-years and for gender groups only in model adjusted with CACs categorically, moreover, neither suPAR nor CRP couldn‘t strengthen model including traditional CV risk factors and CACs by ROC-curve (AUC) analysis for CVE at p = 0.33 and p = 0.32 respectively.

A prospective cohort study by Mehta et al. [181] including 906 asymptomatic South Asian patients with MASALA-study protocol registry investigating possible associations between 4 IBA (Inflammatory Biomarker and Adipocytokines) markers, which are inflammatory markers of hsCRP (High-sensitivity C-reactive protein) and TNF-α (Tumor necrosis factor alpha) and adipocytokines of adiponectin and leptin, with CAC-presence and CAC-severity, observed in unadjusted crude model Leptin tertile 3 vs tertile 1–2 and hsCRP tertile 3 vs T1-2 inversely and TNF-α tertile 3 vs T1,2 directly associated with CAC presence (CACs > 0) yet in models adjusted for demographic features (model-1) and further for conventional risk factors (model-2) associations attenuated and in fully adjusted model 2 when stratified by genders low adiponectin T1 vs T2-3 among female inverse association with CACs > 0 presented and persisted for transformed Ln[adiponectin] as continuous variable in multi-variable adjustment models even further adjustment for BMI and VAT (Visceral Adipose Tissue); similarly in unadjusted models TNF-α T3 vs T1-2 directly and Leptin T3 vs T1-2 inversely associated with CACs > 100 yet these associations attenuates in adjusted models 1 and 2 and when stratified by genders no significant association remained, however, when 243 statin users were excluded inverse association among males became available, nonetheless adding any of the 4 IBA markers as log-transformed continuous variables to AHA/ACC PCE didn‘t improve model predicitivity.

A cohort study by Larsen et al. [182] involving 409 participants aged 44 to 84 years with 329 return for a follow-up examination on mean inter-scan period of 4.5 (0.5) years as examining relations between adipokines (of measured are IL-6, TNF-α, leptin and adiponectin) and CACs at baseline and follow-up progression, demonstrated at each 1 SD increase in IL-6 significantly associated with increasing CACs Rumberger categories even after adjustments for demographics (age and gender), lifestyle (alcohol use, current smoking and regular exercise), Body size (BMI and WHR), CVD risk factors (TG, HDL-c, FBG, SBP and DBP) and Fat Compartments [VFV (Visceral Fat Volume), SFV (Subcutaneous Fat Volume) and intramuscular fat volume] in model 5 (OR:1.49, 95% CI:1.09–2.02) also without modified significantly by gender yet other measured adipokines had no significant association with CACs categories, however, in model adjusted for demographic and lifestyle variables leptin level at each 1 SD increase significantly associated with baseline CACs categories; after inter-scan period square root transformed-CAC volume score difference or progression significantly inversely associated with only adiponectin among baseline adipokine measurements at each SD increase in adjusted model 5 (OR:0.68, 95 %CI:0.51–0.92) and association of adiponectin with CAC progression in model adjusted by age, lifestyle and body size variables modified by gender (p = 0.04) with remaining significant for males (OR:0.57, 95% CI:0.38–0.85) but not for females.

A cross-sectional study by Tibuakuu et al. [183] with size of 935 male participants, of whom are 589 HIV-infected and 346 HIV-uninfected patients, aged mean 53.7 ± 6.9 years examining relations of GlycA level, which was detected by NMR-spectroscopy as a composite marker of systemic inflammation mainly shows glycosylated modifications of 5 common acute-phase reactants of α1-acid glycoprotein, haptoglobin, α1-antitrypsin, α1-antichymotrypsin and transferrin, in coronary artery calcification and plaque characteristics along with markers of inflammatory milieu on which including conventional inflammatory biomarkers of hs-CRP, IL-6, D-Dimer and Fibrinogen, and levels of monocyte activation markers, which increase with Highly Active Antiretroviral Therapy and associates with atherosclerosis, of sCD14 (Soluble-CD14 + ), sCD163 (Soluble-CD163 + ) and CCL2 (Chemokine C-C motif Ligand-2) while participants stratified by HIV-serostatus, reported across quartiles of GlycA significant linear trend was observed with Non-Hispanic black, BMI, HDL-c (Inverse), pack-years of tobacco smoking, eGFR < 60 mL/min/1.73 m2, low-physical activity, high-physical activity (inverse), HCV-infection, BP-lowering medications, glucose-lowering medications, hsCRP, D-dimer, IL-6, Fibrinogen, sCD14, Total CAC-s, total-plaque score, mixed-plaque score and coronary stenosis ≥ 50%; moreover, according to spearman correlations for both HIV-infected and HIV-uninfected participants GlycA significantly correlated, as correlation coefficients in respective order, with HDL-c (-0.10, −0.12), pack-years of tobacco smoking (0.13, 0.20), glucose lowering medication use (0.11, 0.13), hs-CRP (0.36, 0.39), D-dimer (0.14, 0.14), IL-6 (0.27, 0.41), Fibrinogen (0.40, 0.41) and sCD14 (0.24, 0.26) yet GlycA significantly correlated with physical activity status (r = -0.16), HCV-infection (r = 0.15), BP-lowering medications (r = 0.15), CCL-2 (r = 0.17) and HIV-related factors of HIV-RNA ≥ 50 copies/mL (r = 0.09), Nadir CD4 + cell count (r = -0.12), history of AIDS (r = 0.08) and use of protease inhibitors (r = 0.14) only among HIV-infected participants besides BMI (r = 0.22) and Fasting glucose (r = 0.19) only among HIV-uninfected participants; furthermore, according to Poisson-regression analysis GlycA levels per 1-SD increase significantly associated with CACs till highly adjusted model-4 (for demographics[model-1] + traditional risk factors[model-2] + inflammatory markers[model-3] and + monocyte activation markers) but not model-5 (model-4 + HIV-related factors) among overall-cohort and both HIV-serogroups, coronary stenosis ≥ 50% in model-3 among overall-cohort and without significant interaction of HIV-serogroups in model-1 among HIV-infected participants and model-4 among HIV-uninfected participants, Calcified Plaques in model-4 among both overall-cohort and HIV-uninfected participants and mixed plaques only in model-1 among overall-cohort yet GlycA-level per 1-SD increment remained similar for any plaque types and noncalcified plaques; in addition, in sensitivity analysis by linear regression models comparing highest Q with lowest Q of GlycA showed each 1-SD increase of GlycA levels significantly associated with transformed Ln[CACs] till model-3 adjustment in overall-cohort (0.18), Ln[Total-Plaque score] till model-4 adjustment (0.16) with stronger association among HIV-uninfected vs HIV-infected serogroups (0.22 vs 0.10), NCP-s (Non-calcified Plaque score) only in model-1 adjustment for HIV-uninfected serogroup (0.13) with significant serogroup interaction and MP-s (Mixed Plaque Score) till model-4 adjustment in overall-cohort with stronger association for HIV-uninfected serogroup till model-2 adjustment (0.22) but remained similar for Ln[Calcified Plaque score].

#### 9.5.11 Pleiotropic actions

A prospective cohort study by Bell [184] including 6695 participants with MESA-registry into analysis observed HGF (Hepatocyte Growth Factor), which was noted with favorable actions of anti-inflammatory (through inducing secretion of IL-10 and suppressing IL-8 and MCP-1 levels), anti-fibrotic (neutralizing relevant effects of TGF-β1) and pro-angiogenic (role of new blood vessel developement in tissue repair yet may contribute to progression of atherosclerosis) and unfavorable action in pathological vascular calcification involving smooth muscle cells on c-Met receptor/AKT/Notch-3 signaling pathway of both actions potentially serving as biomarker for CVD, at each 1 SD higher baseline level associated with 5.3 AU annual progression rate (95% CI:4.0–6.6, p < 0.001) in adjusted model 1 (for age, ethnicity and gender) and after further adjustment in model 2 (for baseline BMI, smoking status, DM, SBP, Anti-HTN and lipid-lowering medication use, HDL, TC, level of education and physical activity) association weakened to 2.9 AU annual progression yet differential association strength was observed for ethnicity as 3.3 AU in Non-Hispanic White Americans, 5.5 AU in African Americans, nonsignificant in Chinese Americans (p = 0.4) and nonsignificant in Hispanic Americans (p = 0.5).

A longitudinal study of median 4.1 years by Jun et al. [185] including 9297 participants with 19059.3 person-years examining SUA (Serum Uric Acid), which was noted with end-product of purine metabolism, proinflammatory actions of monocyte apoptosis and induction to TNF-α secretion, induction of VSMC proliferation via PDGF, platelet agregation and lysis, and antioxidant feature, in association with development of moderate CACs > 100 as endpoint of study observed in fully-adjusted model (for conventional risk factors and transformed baseline Ln[CACs + 1]) SUA Quartile 4 vs Q1 (>6.5 mg/dL vs < 5 mg/dL; HR:5.93, 95% CI:2.88–12.19), SUA Q3 vs Q1 (5.8–6.5 mg/dL vs Q1; HR:4.80, 95% CI:2.30–9.99), SUA Q2 vs Q1 (5.0–5.7 mg/dL vs Q1; HR:2.56, 95% CI:1.17–5.57) and continuous score of SUA per 1 mg/dL (HR:1.52, 95% CI:1.33–1.73) significantly associated with incident moderately calcified CACs > 100 within longitudinal observation by parametric Cox proportional hazards model per basis of interval censoring, moreover, association of continuous SUA level with CACs > 100 had no significant interaction for covariates of age (<55 years vs ≥ 55 years), gender, DM, Current smoking, HTN, BMI (<25 vs ≥ 25 kg/m2), eGFR (60 ≤ eGFR < 90 vs ≥ 90 mL/min/1.73 m2), CRP (<median 0.07 vs ≥ median 0.07 mg/dL) and follow-up time (<5 years vs ≥ 5 years); furthermore, predictive value of SUA level for incident moderate CACs > 100 by ROC-curve had no significant difference with clusters of conventional risk factors (p = 0.096), nonetheless combining clusters of conventional risk factors with SUA-level significantly improved the predictive power for incident moderate CACs > 100 (from AUC:0.63 to AUC:0.71, p < 0.001) by ROC-curve and cut-off value of SUA-level at 6.2 mg/dL had 60.3% sensitivity and 62.8% specificity.

#### 9.5.12 Oxidative stress

A cross-sectional study by Beloqui et al. [186] including 159 asymptomatic male participants with regular medical work-up on 12 h overnight fasting aged mean 57.7 ± 0.7 years to examine and test relation between NADPH oxidase-mediated superoxide production within phagocytic cells, which was measured on isolated 4*105 peripheral blood mononuclear cells (from venous samples) with chemiluminescence method in ex-vivo environment as inducing ROS productions by PMA (Phorbol Myristate Acetate) with added chemiluminescence enhancer Lucigenin to reflect maximum capacity of the expressed enzyme level for ROS generation and is particularly involved in infiltrating monocytes and macrophages to subendothelial space for induction of procalcifying cellular signaling on osteoblastic differentiation of VSMC and oxidation of LDL-c particles, and CACs reported NADPH oxidase mediated superoxide production increased within CACs > 400 (Median:30.35 counts/s, IQR:13.42–49.15) compared to both CACs 0 (median:14.46 counts/s) and CACs 1–100 (median:17.80 counts/s) yet no significant difference with CACs 100–400 (median:24.85 counts/s, IQR:12.61–48.15), furthermore, in Pearson‘s correlation test adjusted for age NADPH oxidase mediated produced superoxide counts/s had significant bivariate correlations with HDL-c (Rc = -0.190), TG (Rc = 0.310) and CACs in AU (Rc = 0.353), moreover, in multiple linear regression model CACs significantly correlated with Superoxide production in counts/s (Partial-R2:13.7%) and Age in years (Partial-R2:6.9%) both adjusted for age, HDL-c, TG, BMI, smoking, HTN and diabetes.

#### 9.5.13 Body composition

A cross-sectional study by Khaing et al. [187] including 487 Chinese participants aged ≥ 50 years living in Singapore examining associations of regional fat depots, which are DXA-imaging (Dual Energy X-ray Absorptiometry) assessed arm, leg, trunk and total fat mass likewise total body fat percent besides CT-scans assessed EAT (Epicardial Adipose Tissue) at the level of LMCA (Left main coronary artery) along with VAT (Visceral adipose tissue beneath the abdominal muscular wall) and SAT (Subcutaneous adipose tissue superficial to abdominal muscular wall) at L2/L3 level, with having CACs ≥ 100, demonstrated EAT correlated strongly with VAT (cm^2^, r = 0.72) and trunk fat mass (kg, r = 0.66), moderately with total fat mass (kg, r = 0.57), arm fat mass (kg, r = 0.49) and SAT (cm^2^, r = 0.44), and weakly with leg fat mass (kg, r = 0.28) and total body fat% (r = 0.22); all of these listed adiposity measurements significantly correlated with central aortic diastolic blood pressure, FPG (Fasting plasma glucose-Log transformed), HbA1c%, HDL-c (log-transformed) and TG/Log transformed HDL of which TG/Ln[HDL] with VAT (cm^2^) had highest correlation magnitude (r = 0.43) and regarding HbA1c% with leg fat mass (kg) had lowest correlation magnitude (r = 0.10); nonetheless in model 1 (adjusted for age, gender, smoking and hypertension) only VAT (OR:1.43, 95% CI:1.05–1.94) and EAT (OR:1.30, 95% CI:1.03–1.65) at each 1 SD significantly associated with having CACs ≥ 100 yet in model 2 further adjusted for LDL-c and TG/Ln[HDL-c] and model 3 subsequently further adjusted for HbA1c% these 2 associations couldn‘t remain significant; after all of these analyses authors suggested effects of EAT and VAT could be mediated by conventional risk factors.

An observational study by Cosson et al. [188] comprising 410 patients aged mean 57.1 years old, who had ECG-gated cardiac CT without contrast imaging, presented EAT-volume negatively correlated with creatinine clearance rate and HDL-c level on contrary positively correlated with age (years), BMI, TG, pack-years of smoking history and CACs along with significantly positively associated with male gender, overweight/obesity, HTN, dyslipidemia, CACs ≥ 100, ethnicity of Caucasians subsequently Arabic vs other ethnicities and Type 2 Diabetes vs Type 1 or other types of diabetes besides diabetes-related complications of retinopathy (exceptionally lower EAT-volume), nephropathy (higher EAT-volume) and albuminuria (higher EAT-volume) yet other diabetes related complications of renal failure, neuropathy, peripheral arterial occlusive disease and CAD didn‘t have significant associations, moreover, according to multivariate logistic regression analysis EAT volume (per 10 cm3, OR:1.13), age (per year, OR:1.08), cumulative pack-years of tobacco use (OR:1.03), retinopathy (OR:1.89) and macrovascular disease (OR:3.94) and macrovascular disease (OR:3.94) significantly associated with CACs ≥ 100 ≥ to be presented with its symbol.

A cross-sectional study by Nam and Jun [189] involving 67 males with ABAT (Active-Brown Adipose Tissue) under unstimulated conditions and their 1:1 matched control subjects retrospectively selected from 4315 males with general health check-ups to examine relation between ABAT and CAC also in terms of arterial inflammation evaluated by FDG PET/CT imaging protocol, which was initiated with administering FDG at a dose of 0.1 mCi/kg in at least 6 h of fasting subsequently 60 min after FDG injection low-dose CT was used for attenuation correction and precise anatomical localization to discover emission scan in 3-dimensional mode and PET data was acquired with high-resolution whole-body scanner afterward reconstructed, based on principles of FDG-uptake by inflammatory state, reported subjects participants with ABAT vs matched controls significantly associated with lower prevalence of fatty-liver disease and CACs > 0, and significantly lower levels of SUVmax (Standardized Uptake Volume; 1.72 ± 0.23 vs 1.88 ± 0.23), identified on bilateral supraclavicular fossa and paravertebral thoracal vertebra, TBR (Target-to-Background Ratio; 1.18 ± 0.14 vs 1.29 ± 0.13), is division of arterial and venous SUVmax measurements, and bsSUVmax (Blood Subtracted SUVmax; 0.25 ± 0.18 vs 0.41 ± 0.16), is subtraction of arterial and venous SUVmax measurements; similarly, ABAT negatively correlated with TBR (r = -0.244, p = 0.046) and bsSUVmax (r = -0.269, p = 0.028); moreover, multivariable logistic regression analysis of clinical parameters, significant with CACs in univariate analysis (of are ABAT, γ-GT, FBG, HDL-c, BMI, Fatty-Liver and WC), demonstrated ABAT (OR:0.19, p = 0.024) and HDL-c (OR:0.95, p = 0.037) significantly associated with CACs > 0 on which yet FRS and traditional risk factors couldn‘t achieved significant association.

A cross-sectional study by Chung et al. [190] with population size of 1282 subjects aged mean 58.1 ± 9.3 years probing relations of obesity, sarcopenia (% of appendicular skeletal muscle determined by bioelectrical impedance analysis in body weight<2-SD below the gender specific mean healthy young adults’ measurements) and sarcopenic obesity (SO) to CACs beside on clinical and laboratory parameters, reported in comparisons of 4 groups (of which are obesity[n = 414], sarcopenia[n = 14], SO[n = 108] and control[n = 746]) SO group had significantly highest male-proportion, hypertension-prevalence and levels of AST, ALT, TG, hs-CRP, and prevalence of having high CACs ≥ 100 and lowest level of HDL-c while sarcopenia group had highest mean-age, highest prevalence of DM, highest level of FPG and low and lowest creatinine level; moreover, in row across Control-group, Obesity, Sarcopenia and SO groups significant increase in prevalence of high-CACs ≥ 100 was presented, furthermore, in multivariate analysis of 4 groups in relation to having high CACs ≥ 100 for model-1 (adjusted for age and gender) obesity (OR:1.54) and SO (OR:2.61) groups significantly associated and for further-adjusted model-2 (+HTN and DM) and model-3 (model-2 + Dyslipidemia and Creatinine) obesity-group attenuated in contrast SO-group remained significant (OR:1.92 for both model); in addition, in subgroup analysis for stratifications by gender and age only significant difference compared to control-group was observed among male patients on SO-group (OR:2.20) rather than sarcopenia and obesity groups and for multivariate logistic regression dichotomized obesity group by sarcopenia status compared to control-group both obesity without sarcopenia and with sarcopenia groups significantly associated with increased risk of having high-CACs ≥ 100 (OR:1.53 and OR:2.58, respectively) in model-1 yet in further adjusted model-2 and model-3 SO-group rather than obesity without sarcopenia remained significant (OR:1.91 in both adjusted models); and in summary of these findings neither sarcopenia nor obesity could overcome further adjustment for HTN and DM other than age and gender yet their concurrent presence additively or synergistically could increased risk of having CACs ≥ 100 even after additionally adjusted for dyslipidemia and creatinine.

A cross-sectional study by Ko et al. [191] including 6686 females and 24,422 males in total 31,108 participants aged mean 41.3 years analyzing relation of relative muscle mass and CACs, reported SMI (Skeletal Muscle Index), which is proportion of muscle mass in body weight and detected by BIA (Body Impedance Analyzer), inversely associated with CACs as such compared to reference highest Q4-SMI in age and gender adjusted model other quartiles of Q1 (OR:2.22), Q2 (OR:1.46) and Q3 (OR:1.25) significantly associated with increased CACs (Transformed Ln[CACs + 1]) on which per 1-SD decrease in SMI (3.81) posed greater Ln[CACs + 1] (OR:1.44), in multivariate further adjusted model-1 (for + center of examination, year of screening examination, smoking status, alcohol intake, physical activity, educational level, total calorie intake and FH of CVD) compared to reference Q4-SMI both Q1 (OR:2.27) and Q2 (OR:1.46) significantly associated with Ln[CACs + 1] besides significant linear association of per 1-SD decrease in SMI (OR:1.46), and in further adjusted multivariable model-2 (for + HOMA-IR and LDL-c) only Q1 vs Q4 posed significant association for Ln[CACs + 1] (OR:1.48) besides linear association of per 1-SD decrease in SMI (OR:1.21); according to multinomial logistic regression models stratifying participants by CACs categories of ≤ 100 and > 100 in CACs 1–100 category in both age and gender adjusted model and adjusted model-1 (respectively) Q1 (OR:1.32, OR:1.33) and Q2 (OR:1.11, OR:1.12) both vs Q4 significantly associated with higher prevalence of CACS 1–100 vs CACs = 0 also valid significant linear association between SMI (for each SD decrease) and CACs 1–100 (OR:1.14, OR:1.15) yet in model 2 no significant association was observed across Q-SMI scores, nonetheless in CACs > 100 category only Q1-SMI vs Q4-SMI comparison remained significant (OR:1.37) for CACs > 100 vs CACs = 0 after further adjustment in model-2 (for HOMA-IR and LDL-c); furthermore, gender, smoking-status, alcohol intake(<20 g/d vs ≥ 20 g/d), HEPA Health Enhancing Physical Activity, yes/no), HOMA-IR (<2.5 vs ≥ 2.5) and hs-CRP (<1.0 mg/L vs ≥ 1.0 mg/L) had no significant interaction with associations between Q-scores of SMI and Ln[CACs + 1] yet age stratification by ≥ 50 years vs < 50 years attenuated and only Q1-SMI vs Q4-SMI remained significant rather than Q2 or Q3 vs Q4-SMI scores; in addition, as noted by authors low physical activity causes IR, low muscle mass, which impairs insulin sensitivity and precipitates IR since muscle tissue is primary whole-body disposal site of glucose by insulin-sensitivity, loss of muscle-strength, deteriorating hemodynamic and HTN, and concurrently IR further aggravate muscle loss through several possible mechanisms exemplified by mitochondrial dysfunction (due to IR), suppression of anti-apoptotic mTOR-pathway (due to IR) and activation of ubiquitin–proteasome pathway mediated muscular protein degradation (due to inadequate IGF-1 stimulation in IR).

A cross-sectional study by Murakami et al. [192] involving 189 participants with known or suspected CVD or established DM aged mean 66.7 ± 10.2 years splits into 112 middle-aged patients 40 to ≤ 69 years of age and 77 patients ≥ 70 years of age, examining relations of right-RSFV to VATV (RSFV/VATV) ratio index, which reflects perivascular relative distribution of VAT in renal sinus area as ectopic fat deposits or extension of VAT in close-proximity to kidney featuring paracrine actions of dysfunctional adipose tissue deposition conducive to functional impairment in kidney, with CACs and CVD-risk factors, reported age, male gender (exceptionally by Chi-square test) and 4 focal adipose tissue accumulation indices of RSFV (right Renal Sinus Fat Volume, cm3), RSFV/VATV ratio (RSFV to Visceral Adipose Tissue Volume), Log_10_[RSFV/VATV] and V/S ratio (Ratio of Visceral Adipose Tissue area to subcutaneous adipose tissue area) positively and RSFV/BMI ratio also eGFR inversely associated with higher prevalence of CACs > 10 vs. CACs ≤ 10 among both overall-study population and middle-aged participants by Student‘s *t*-test but not for elderly participants, moreover, CACs significantly correlated with only RSFV/VATV ratio index rather than RSFV, VATV and V/S ratio among study population (r = 0.23) and middle aged participants (r = 0.42) but no significant association for elderly and relevantly in study population RSFV/VATV ratio significantly correlated with pack-years of smoking (r = 0.18, but not for middle-aged participants), TG (r = -0.20, excepts middle-aged participants), LDL-c (r = -0.22, excepts elderly) and BMI (r = -0.27, excepts elderly); furthermore, by multivariable logistic regression analysis for middle-aged participants Log_10_[RSFV/VATV] rather than RSFV and VATV significantly associated with greater prevalence of CACs > 10 vs CACs ≤ 10 in model adjusted for conventional risk factors (age, gender, pack-years of smoking, DM, HTN, BMI, kidney volume[sign of renal functional impairment], BMI) and also with absolute CACs RSFV/VATV ratio significantly associated regardless of conventional risk factors; and these findings may suggest RSFV/VATV ratio index intricately linked with metabolic syndrome besides impairing renal functions could alternatively sign coronary artery calcification.

#### 9.5.14 Hematologic and hemostatic indices

A cross-sectional study by Serrano et al. [193] examining possible associations of 2 inflammatory hematological indices of NLR (Neutrophil-to-Lymphocyte ratio) and PLR (Platelet-to-Lymphocyte ratio) variables, which can reflect lymphocyte depletion because of apoptotic elimination in response to persistent inflammatory setting of which besides can increase neutrophils in circulation and lead proliferation in megakaryocytic series with relative thrombocytosis as discussed by authors markers in acute episode of inflammation or acute physiological stress, to CACs > 0 retrospectively including 247 patients, who are asymptomatic for any CVD-disease, stable, at low-intermediate risk for CHD-events and having WBC within normal range, from database of a single-center to test dynamic activities of inflammatory processes other than vascular injury at site in atherosclerosis under chronic low level inflammatory settings, demonstrated according to multivariable logistic regression analysis adjusted for conventional CVD risk factors NLR (OR:1.85, 95% CI:1.10–3.09) but not PLR significantly associated with presence of CACs > 0, moreover, within stratification by CACs-categories (CACs 0, Mild: CACs 1–100, Moderate: CACs 101–400 and Severe: CACs > 400) hematological tests of WBC-counts, absolute and relative neutrophil or lymphocyte counts and Platelet-absolute count remained similar, nevertheless in unadjusted models patients with only severe CACs > 400 category rather than other categories PLR level noticeably increased by ANOVA and only severe CACs > 400 vs zero CACs = 0 mean age significantly increased.

An observational study by de Almeida-Pititto et al. [194] including 998 participants aged mean 45.8 years with low-to-moderate risk level investigating relations of E-selectin, which only transcribed on endothelium as adhesion molecule mediates leukocytes adhesion to endothelium and promotes migration of leukocytes to subendothelial region in early stage of atherogenesis so as to incite , with cardiometabolic risk profile and CACs, reported across tertile categories of E-selectin level 1 to 3 significantly associated in linear trend with BMI, WC, SBP, DBP, FPG, HbA1c%, TC, LDL-c, TG, fasting insulin, HOMA-IR, IL-6/IL-10 ratio and TNF-α/IL-10 ratio, and inversely with proportion of Women but had no significant association with Age (years), 2-h plasma glucose, HDL-c and CRP, moreover, participants with metabolic syndrome and metabolic syndrome components of BMI ≥ 25 kg/m^2^, hypertension, pre-diabetes and hypertriglyceridemia had significantly greater prevalence with increasing tertiles 1 to 3; additionally in multivariable linear regression model including IR-index of HOMA-IR or fasting insulin level, male gender and BMI significantly associated with E-selectin concentration but WC also age in contrast with BMI couldn‘t modify independent predictive value of IR-index for E-selectin concentration; furthermore, E-selectin Tertile-3 vs Tertile 1-to-2 significantly associated with higher prevalence of CACs > 0 and CACs > 10 but only nonsignificant positive trend with CACs > 100; and in summary of these findings elevated E-selectin concentration could show early signs of cardiometabolic abnormalities within normal or near-normal limits of traditional metabolic parameters and developing structural coronary lesions cumulatively determined by CACs.

A follow-up study by Bielinski et al. [195] in population size of 6025 participants including 2383 non-Hispanic white American, 700 Chinese American, 1631 African American and 1303 Hispanic American overall aged mean approximately 64 years with mean 10.1 years of follow-up period and investigating relations of P-selectin, which is found in primarily platelets as within alpha-granules then Weibel-Palade bodies of endothelial cells and as a cellular adhesion molecule acts as procoagulant and mediates Leukocyte-ligand (PSGL-1) tethering subsequently integrin action of firmly adhering leukocytes toward coagulation steps clumping/cross-linking leukocytes and platelets also involving actions of soluble form of P-selectin, with traditional CV-risk factors, CVD-events and subclinical CVD disease or atherosclerosis determined by CACs and cIMT, reported across baseline quintiles of P-selectin concentration Q1 to Q5 significantly associated in linear trend with higher mean SBP, DBP, TC, LDL-c, HbA1c and TG and higher prevalence of presence of HTN particularly uncontrolled hypertension, diabetes, current and former smoking yet inversely associated with % of female participants; furthermore, per 1-SD increase in baseline plasma P-selectin concentration significantly associated with progression of CACs (75 AU) by Tobit linear regression model and increased CHD-events (HR:1.63) by COX proportional hazard regression model both results in model adjustment for traditional risk factors (of Age, Gender, BMI, SBP, HTN-treatment, TC, LDL-c, HDL-c, current smoking and diabetes status), moreover, according to Kaplan-Meier curves displaying cumulative incidence of CHD-events by Quintiles of P-selectin from early times of follow-up till 4‘th years in cohort both Q5 and Q4 quintiles with similar rates had distinctly greater cumulative incidence than other quintiles and subsequent to 4‘th year Q5 overcome the minor difference with Q4 and toward year of 10 stratification of cumulative incidence rates by increasing P-selectin Quintile categories became visible.

#### 9.5.15 Transcriptional regulation

A cross-sectional analysis by Howlett et al. [196] including 24 participants, who had CT-calcium scoring and coronary angiography based on routine work-up for investigation of chest-pain, aged younger than 65 years on normal serum calcium concentration without reduced GFR ≤ 60 mL/min and history of predefined or suspected any CVD, systemic inflammatory disease, active infection and active malignancy to detect any plausible circulating mi-RNAs, which was isolated from peripheral leukocytes rather than microvesicles stores majority of miRNAs, reported Torray microarray analysis with bioinformatic analysis revealed CACs ≥ 100 vs CACs = 0 had significant downregulation of miRNAs 138-2p, 1181, 6816 and 8059 then qRT-PCR analysis supported only miRNA-8059 downregulation and additionally according to DIANA Tools miRPath modulated mRNA pathways by miRNA-8059 were estimated as metabolism of xenobiotics by cytochrome P450, biosynthesis of unsaturated fatty acids, fatty acid metabolism, chemical carcinogenesis, glycosaminoglycan biosynthesis-keratan sulfate, glycosaminoglycan degradation, nucleotide excision repair, glycosphingolipid biosynthesis and proteoglycans in cancer.

### 9.6 Family history of Premature-CHD

A cross-sectional study by Mamudu et al. [86] involving 2563 participants aged 46–74 years (13.7% Diabetes, 65.7% FH of premature-CHD) recruited from Central Appalachia region of United States having high risk of diabetes above US national average to investigate the relations between cardiovascular disease risk factors perusal or combined with nonzero CACs on which risk factors categorized as two modifiable risk factors (smoking, sedentary lifestyle), 3 medical conditions (Obesity, HTN, Hypercholesterolemia) and Family History of premature-CHD if present each item was assigned 1 to RFI (Risk Factor Index) ranging from 0 to 6 reflecting combined effect, reported presence of DM increased risk of having non-zero CAC ≥ 1 for risk factors of obesity (57.3% vs 33.7%), HTN (75.9% vs 53.8%), hypercholesterolemia (71.4% vs 59.1%) and sedentary lifestyle (50.2% vs 35.2%) excepts smoking and family history of premature-CAD, moreover, in multinomial logistic regression model of 6 risk factors (adjusted for age and gender) compared to reference category as absence of risk factor with CACs = 0 among subjects without diabetes obesity, HTN, hypercholesterolemia and smoking significantly associated with non-zero CACs ≥ 1 and among subjects with diabetes these associations gained strength and relation of sedentary lifestyle was also modified into significant yet relation of FH of CAD with CACs ≥ 1 vs 0 remained insignificant, furthermore, as the number of risk factors ≥ 2 increases risk of having non-zero CACs ≥ 1 increased incrementally and again patients with diabetes had greater risk development; and as a summary of these analysis could suggest DM-status couldn‘t modified relation of FH of premature-CHD with presence or absence of CACs on either bivariate or multinomial LR among population with high prevalence of FH for premature-CHD.

A cohort study by Paixao et al. [97] involving 2390 participants on mean follow-up time of 8.0 years demonstrated both non-zero CAC > 0 and having family history (FH) of premature-CHD defined as first-degree relative with history of MI perusal and additively associated with higher odds of CVD-events, moreover, FH regardless of adjustment for CAC, traditional risk factors, FRS and age remained significantly associated with increased risk of CVD-events. Consistently, Cohen et al [98] as longitudinal analysis of 3815 participants with CAC = 0 at baseline over the median 10 years of follow-up period observed family history of CHD associated with higher odds of both CVD (HR:1.73) and CHD (1.60) events on adjustment for age and gender.

A cohort study by Dudum et al. [66] following 14,169 participants with reported family history of premature CHD on follow-up period of average 11.6 years, reported the number needed to screen (NNS) to detect CAC > 100 as 9, and in multi-variable analysis of COX proportional hazard models CAC > 100 compared to CAC = 0 had higher risk for all-cause mortality in 2.2-fold, CVD-specific mortality in 4.3-fold, and CHD-specific mortality in 10.4-fold.

A multiple-reports of 2 different studies by Mulders et al. [218] involving a cross-sectional study of 363 Caucasian participants with FH of premature CHD and their age and gender matched 341 participants without FH recruited from 3 different centers in Netherlands and Canada aged mean 52.5 ± 6.2 years to examine relation of CAC and FH of premature CHD, and a cohort study of 318 CACs = 0 and 516 CACs > 80‘th percentile participants in case-control design with mean follow-up period of 3.5 years at which 405 participants with FH of CHD were compared with their age and gender matched 429 participants without FH of CHD, reported in cross-sectional study participants with FH vs without FH had significantly greater rates of CACs > 0 (61.4% vs 38.6%), CACs > 80‘th percentile (33.9% vs 17.0%) and > 95‘th percentile (11.3% vs 5.3%), and in case-control study for both participants with FH and without FH at CACs = 0 CVD-event rate remained low but at CACs > 80th percentile by Cox proportional-hazard model (adjusted for age, gender, smoking, HTN, DM and cholesterol) participants with FH of premature CHD vs without significantly associated with increased risk of ASCVD-event (HR:2.08, 95% CI:1.09–3.87); and authors suggested combined use of FH of premature CAD and CACs testing in detection of high risk individuals among asymptomatic individuals.

A cohort study by Pandey et al. [226] including 5099 participants, whom are 2466 no family history of CHD, 1631 late onset family history of CHD and 1002 premature family history of CHD, recruited from MESA cohort on mean follow-up time of 3.1 years to investigate relations of family history and it‘s source with CACs incidence and progression, reported participants with premature FH of CVD as 7.24 (per 100 person-years), 6.56 (per 100 person-years) and 5.87 (per 100 person-years), and regarding source of FH participants with parental history of premature CHD associated with CACs > 0 incidence after model adjustment (of age, gender, ethnicity, MESA site, follow-up duration, education, conventional risk factors and Framingham risk score) yet premature CHD in siblings couldn‘t have significant association after adjustment beyond demographics for conventional risk factors and FRS, moreover, for baseline CACs > 0 in unadjusted models compared to having no family history of CHD late onset FH and premature FH had incrementally increased significant trend and according stratification by source of premature FH sequentially No-FH, Parental-FH, FH in Sibling and combined Parental and Sibling FH had increased annualized CACs change in significant trend, furthermore, in median regression analysis in adjusted model-2 (of demographic and conventional risk factors) compared to participants with no family history premature-FH had greater CAC progression than late FH (in CAC-volume score 14.3 vs 8.1) yet further adjustment for logarithm of baseline CAC score attenuated this association, and regarding source of FH sibling FH of premature CHD had significant median CAC progression (17.0 in CAC-volume score) but either parental FH or combined sibling and parental FH didn‘t have significant association.

A cohort study by Khera et al. [217] including 3 population-based cohort studies of DHS (Dallas Heart Study), Prospective Army Coronary Artery Calcium Project and MESA cumulatively 5335 participants aged mean 51.0 (7.5) years to develop risk assessment model for composite ASCVD outcome by integrating PCE, CACs, Family History of premature-CAD and hs-CRP with bootstrapping technique into calculator termed as Astro-CHARM (Astronaut Cardiovascular Health and Risk Modification) and testing its validity along with another cohort study of FHS (Framingham Heart Study) cumulatively 7382 participants with median follow-up period of 10.9 years, reported in c-statistics full Astro-CHARM model (AUC = 0.817) had higher prediction compared to PCE (AUC:0.784), CAC-model (AUC:0.720) and PCE + CAC (AUC:0.813) even Astro-CHARM model without hs-CRP had better prediction (AUC:0.826) and Astro-CHARM had significant NRI of 0.121 contributed by 12% true up-classification without significant down-classification, moreover, in sensitivity analysis among intermediate risk group (10-year 5–15% ASCVD-risk) Astro-CHARM had NRI of 0.141 contributed by 15% true up-classification.

A *meta*-analysis of GWAS by Iperen et al. [219] including 69 studies of European populations on 60 non-established risk factors or traits determined in literature searched through PubMed database subsequently searched and displayed on NHGRI (National Human Genome Research Institute) GWAS Catalogue with SNP, effect size, standard error of effect size, risk allele and it‘s frequency under assumption of significance level of p < 5E-08 to examine relations between identified SNPs linked with traits and risk of CAD events through summary statistic method weighting each SNP effects on the risk factor presented with effect size, standard error of effect size and risk allele subsequently analyzed with validation dataset retrieved from publicly available CARDIOGRAM GWAS database involving 22,233 CAD-events and 64,762 control subjects under assumption of p < 8.33E-04, reported 15 out 60 traits significantly associated with CAD-events at per 1-SD increase in continuous scale listed in decreasing strength of association as LDL-c (OR:1.542), CAC (OR:1.906), TG (OR:1.399), Lp(a) (OR:1.249), DBP (OR:1.486), SBP (OR:1.492), Lp-PLA_2_ (OR:1.377), HDL-c (OR:0.789), T2DM (OR:1.221), Plaque (OR:1.348), Height (OR:0.866), BMI (OR:1.082), Factor-8 (OR:2.249), vWF (OR:0.786) and Mean Arterial Pressure (OR:1.342), however, after removal of pleiotropic SNPs within either upstream risk factors or downstream factors along causal pathways if linkage disequilibrium with other SNPs by r^2^ > 0.5 exists only 6 out of 15 traits remained significant for CAD-events at per 1-SD increase in continuous scale listed in decreasing strength of association as CACs (OR:1.906), Lp(a) (OR:1.293), LDL-c (OR:1.293), TG (OR:1.448), Plaque (OR:1.348) and Height (OR:0.867) yet in row of weakening associations BMI, DBP, T2DM, HDL-c, SBP, Lp-PLA_2_, mean arterial pressure, Factor-8 and vWF trimmed down.

A case-control GWAS study Choi et al. [220] involving discovery set of 400 participants with 300 control and 100 severe CAC participants and validation set of 1288 participants with 1061 control and 227 severe CAC participants on which for both set control and severe CAC groups were defined respectively as CACs < 50th percentile CAC > 90th percentile to reveal single nucleotide polymorphisms (SNPs) associated with severe CAC per set through sequential steps of genomic DNA amplification, genotyping and quality control of detected SNPs, Taqman PCR-assay of replication set for SNPs with valid Bonferroni correction for genome-wide significance (p = 9.08E-04) at discovery set and endpoint fluorescent readings, reported GWAS of discovery set in additive genetic model of logistic regression model (adjusted for age, gender, HTN and DM) identified only one SNP (rs10757272) on chromosome 9p21.3 within intronic region of CDKN2B-AS1 (Cyclin-dependent kinase inhibitor 2B anti-sense RNA gene) passing Bonferroni correction at p = 7.55–08 (OR:3.24, 95 %CI: 2.11–4.97) and this SNP remained significant in replication set by PCR-assay (p = 0.036).

### 9.7 Aging and CAC across lifespan

Tota-Maharaj et al. [109] with baseline of recruited 6809 MESA-participants followed along median 8.5 years, who stratified study population into age groups age groups (defined by 45-54, 55-64, 65-74 and 75-84) and CAC groups (0, 1–100 and > 100), observed across increasing age groups as 75–84 years old age group compared to 45–54 years old age group, proportion of non-zero CAC score, CAC > 100 and CHD-event risk increased, but at each age-group increase in CAC from 0 to > 100 resulted in similar increase in CHD-event rate and CVD-event free survival in Kaplan-Meier curves as such age-related increase in CHD-event risk attenuates significantly after adjustment for CAC-score with similar survival rates, moreover, CAC = 0 in the 75–84 years old age group had lower incidence of CHD-event than any non-zero CAC groups of 1–100 and > 100 in age group of 45–54 years old.

Miedema et al. [110] including 22,346 CAC Consortium participants into cohort study with mean follow-up period of 12.7 years, where the baseline was stratified by age into age groups of 30-to-39 and 40-to-49 years, observed across increasing age strata associated with higher probability of having any non-zero CAC 1–100 (29.3% vs. 19.1%) and CAC > 100 (8.4% vs. 2.7%) and higher risk of CHD, CVD and all-cause mortality but CVD,CHD and all-cause mortality rates at CAC > 100 among age group of 30–39 years remained similar with age group of 40–49 years, moreover, CAC > 100 compared to CAC = 0 significantly associated with higher prevalence of each of the 5 traditional risk factors as hypertension, hyperlipidemia, current smoking family history of premature-CHD and diabetes, higher cumulative number of presenting risk factors and higher risk of CVD, CHD and all-cause mortality.

A cohort study by Hartiala et al. [112] including 589 participants recruited from prospective study of Cardiovascular Risk in Young Finns Study into analysis, whom 27 years of change in risk factor levels and CAC-scores from adolescence aged 12–18 years to adulthood aged 39-to-45 years were measured, reported a non-zero CAC score significantly associated with higher SBP, total cholesterol and non-HDL-c for both adolescence and adulthood measurements, higher LDL-c and higher total cholesterol/HDL-c ratio for adolescence measurements, and higher DBP and pack-years of smoking in adulthood measurements, moreover, in multivariable logistic regression model per 1-SD change in adolescence LDL-c (OR:1.34) and SBP (OR:1.38) associated with increased odds of non-zero CAC > 0 in adulthood measurements; and by regional CAC assessment in adulthood measurements LDL-c levels measured in both adolescence and adulthood significantly correlated with both LAD-score and total-CAC score.

### 9.8 Neighborhood Setting, social interactions and psychosocial factors

On influence of neighborhood environments on developing type 2 diabetes mellitus (T2DM) a MESA cohort by Christine et al. [87] involving 5124 participants on median follow-up of 8.9 years, reported that an interquartile increase in the summary healthy food environment measures and physical activity measures associated with lower probability of developing T2DM as respectively 12% (CI: 0.79–0.95) and 21% (CI: 0.69–0.90) in adjusted model (of baseline age, sex, income, educational level, race/ethnicity, alcohol use, and smoking), however, summary measures for social environment had largely unassociated.

On relation of SES (Socioeconomic Status) and alcohol consumption several cohort studies showed some incoherent results. Brenner et al. [79] revealed that one standard deviation increases in neighborhood SES associated with a reduction in the probability of current alcohol use for both genders while Brenner et al. [78] discerned that, living in a neighborhood in the highest disadvantaged tertile associated with a lower probability of current alcohol use. However, both studies [78], [79] consistently reported that there is no association between neighborhood SES and overall weekly alcohol use, increasing neighborhood SES associate with decreasing weekly beer consumption, alcohol outlet density in neighborhood doesn’t change current drinking regardless of gender; for men higher income associate with heavier daily use, whereas higher education associate with lower amount weekly consumption; however for women higher income and education level associate with heavier daily and more weekly alcohol consumption.

A cohort study by Wing et al. [221] including 5950 participants recruited from MESA study on mean 2.7 (0.7) time point of scan with mean inter-scan period of 3.5 (3.1) years cumulatively 12-years of follow-up period to investigate influence of social and physical characteristics of neighborhood environment in CACs development through comparing geographic information systems (ArcGIS) determined density of healthy food stores and recreational facilities within 1 mile of participants‘ home (units per square mile) and survey-based neighborhood scales per participants of rating (1to5) for availability healthy food, walking environment, safety and social cohesion within 1 mile of participants‘ home with CACs imaging, reported proportion of participants with CACs > 0 at baseline significantly associated with density of healthy food stores quartiles inversely and social cohesion and safety quartiles gradually yet had no significant associations with quartiles of density of recreational facilities, availability of healthy foods and walking environments, moreover, mean absolute CACs at baseline significantly and inversely associated with quartiles of density of healthy food stores and availability of healthy foods but not with density of recreational facilities, walking environment and social environment, however, mean annual change in CACs had no significant association with any of the presented 5 neighborhood quartile scores, furthermore, in econometric fixed effects model allowing testing simultaneous mean changes within person in exposures and outcomes per 1-SD increase only density of healthy food stores among presented 5 neighborhood characteristics significantly associated with within-person changes in CACs in models adjusted progressively more proximal to outcome more like a causal pathway at −19.99 in model-1 (age, marital status, income, working status and CT-scanner type), −18.99 in model-2 (model-1 + moderate/vigorous physical activity and cigarette smoking), −19.41 in model-3 (model-2 + Center for Epidemiological Studies Depression Scale score) and −17.60 in model-4 (model-3 + TC/HDL-c, BMI, HTN, DM and lipid-lowering medication use).

A cohort study by Abdulla et al. [222] including 6814 MESA participants with median follow-up period of 9.4 years to investigate potential influence of questionnaire evaluated 4 personality traits of anger (Spielberger Trait Anger scale with 10 items), hostility (Cook-Medley Hostility scale with 8 true/false questions), anxiety (Spielberger Trait Scale with 40-item questionnaire) and depression (CES-D with 20 items) on incidence of CACs > 0 and progression of CACs, reported CACs > 0 at baseline had no significant association with any of the trait likewise incident CACs > 0 had also no significant association with any of the trait per unit increase, moreover, difference in CACs between initial examination and final outcome or ΔCACs had no significant association with any of the personality trait in any adjusted model, nonetheless, in univariate linear regression only anger had significant association with ΔCACs.

Dragano et al. [100] with 11,263 participants revealed male patients living in an area close to a major road ≤ 100 m as a measurement of traffic-exposure and high neighborhood unemployment rate had 2.12 times higher risk of CAC ≥ 75′th percentile compared to reference group of men living in area with low-unemployment and distant to a major road > 100 m, men with ≤ 13 years educational attainment and living in high traffic exposure (≤100 m) versus men with ≥ 14 years education and living in low traffic exposure (≥100 m) 1.85 odds of CAC ≥ 75′th%; moreover, among women with low traffic-exposure high neighborhood-unemployment rate versus low rate with 1.31 odds of having CAC ≥ 75′th% and low-individual income versus high individual income with 1.36 odds of CAC ≥ 75′th% were presented.

A cross-sectional study by Jones et al. [101] including nonsmoking 5032 participants aged mean 62.5 (10.3) years recruited from MESA cohort through excluding participants with self-reported current smoking and urinary cotinine concentrations ≥ 200 ng/mL to investigate effects of SHS (Second Hand Smoking) exposure on inflammatory markers, subclinical atherosclerosis and PAD, reported across SHS severity (1 h/w, 2–3 h/w, 4–11 h/w and ≥ 12 h/w) hs-CRP level, hsCRP ≥ 2 mg/dl incidence, IL-6 level, fibrinogen level, common c-IMT in mm, CAC > 0 incidence and CAC ≥ 75′th percentile incrementally increased, however after model adjustments CAC > 0 incidence and CAC ≥ 75′th percentile lost its statistical significance, nonetheless, in adjusted model-1 (of age, gender, ethnicity, study-site, education level and income) GM ratio of internal cIMT and GM ratio of common cIMT, in adjusted model-2 (of model-1 + HTN-medication, SBP, DM, LDL-c, treatment for dyslipidemia, physical activity and smoking status) GM ratio of hsCRP level and IL-6, and in adjusted model-3 (of model-2 + BMI) OR of hsCRP ≥ 2 mg/L remained significant, moreover, in adjusted model-3 urinary cotinine level significantly associated with GM ratio of Fibrinogen (mg/dL), odds ratio of ABI ≤ 0.9 (OR:2.21), combined ABI ≤ 0.9 and ABI ≥ 1.4 (OR:2.10)

A cross-sectional study by Tsao et al. [102] including 221 asymptomatic participants without documented DM or CVD as 114 subjects living in urban area in Taipei city of Taiwan with mean temperature and humidity of respectively 23 Celsius and 73% themed urban staff member (USM) and 107 subjects living in forest environment in Nantou County of Taiwan with mean temperature and humidity of respectively 17 Celsius and 89% at 1150 m elevation themed forest staff member (FSM) to investigate comparable effects of having ≥ 1 year long-term exposure of forest environment or urban environment on cardiovascular health and health related quality of life through environmental monitoring, examinations, questionnaires, OGTT, cardio-ankle vascular index (CAVI) and carotid artery IMT evaluations, reported USM compared to FSM significantly associated with lower serum cholesterol level, lower risk of cholesterol ≥ 200 mg/dl, lower serum LDL-c, lower probability of LDL-c ≥ 130 mg/dl, lower fasting plasma glucose level, lower prevalence of impaired glucose tolerance, lower probability of pre-diabetes mellitus status, lower exposure to air pollutants of SO_2_, NO, NO_2_, NO_x_, CO, PM10, and O_3_ but similar to indoor-urban environment, self-reported better physical health domain in WHO-Health quality assessment questionnaire, higher working hours per week, and lower amount of coffee consumption against higher consumption of tea and alcohol, moreover, the study measured in forest group compared to urban group significantly lower ABI, lower mean c-IMT in ICA, and lower maximum and mean of IMT.

A cross sectional study by Wang et al. [103] including 8168 participants aged mean 56.9 (10.4) years recruited from prospective cohort of CREATION at Fuwai Hospital in Beijing in China to test the hypothesis suggesting exposure to air-pollution or traffic associates with CVD and CAC progression through 10-year temporal trend acquired by satellite images, geographic features, ground level observations and meteorological data for long-term cumulative exposure estimations, reported long term exposure to air pollutants and traffic at each increasing level for PM_2.5_ per 30 µg/m^3^, NO_2_ per 20 µg/m^3^, O_3_ per 15 µg/m^3^ and distance to major road per 50% decrease significantly associated with both higher CAC score as 29.6%, 33.2%, 52.4% and 10.8% respectively, and higher rates of presence of any non-zero CAC with OR:1.28, OR:1.27, OR:1.12 and OR:1.04 respectively; moreover, they demonstrated male gender, older age > 60 years, and diabetes history pronounced the associations between CAC score and exposures to PM_2.5_ and NO_2_.

### 9.9 Non-Cardiovascular diseases and CAC

A cohort study by Handy et al. [106] including consecutive 6814 MESA study participants on median follow-up period of 10.2 years reported non-cardiovascular diseases of cancer, CKD, pneumonia, COPD, DVT/PE, dementia and hip fracture significantly developed up-stratification to upper CAC stratums across CAC = 0, CAC = 1–400 and CAC > 400, and in adjusted model-3 (of age, gender, race, health insurance status, socioeconomic status, educational attainment, income level, BMI, physical activity, diet, smoking status, smoking history and traditional cardiovascular risk factors) doubling of Log-transformed CACs (log_2_^(CACs+1)^) significantly associated with higher HRs for cancer (1.04), CKD (1.07), pneumonia (1.07), COPD (1.10), dementia (1.06), hip-fracture (1.10) and any non-CVD (1.06) in adjusted model-5 (of model-3 + number of medication use + SBP, DBP, TC, HDL-c, DM, antihypertensive use, lipid-lowering therapy and aspirin use) but in age sensitive interval analysis doubling of CAC only for ≥ 65 years of age group remained significant with increased risk of diagnosing cancer (1.04), CKD (1.07), pneumonia (1.08), COPD (1.09) and any non-CVD (1.06), moreover, CAC > 400 vs CAC = 0 posed significant risk of diagnosing cancer (1.53), CKD (1.70), pneumonia (1.97), COPD (2.71), hip-fracture (4.29) and any non-CVD (1.80) in described model adjustment, and a CAC = 0 compared to any CAC > 0 significantly lowered risk of diagnosis for cancer (0.76), CKD (0.77), COPD (0.61), hip-fracture (0.31) and any non-CVD (0.75) after the model-5 adjustment.

A cohort study by Kuller et al. [113] including 532 participants of longitudinal CHS-Cognition Study (CHS-CS) aged ≥ 80 years on follow-up period of ≥ 10 years to investigate relation of CACs severity and dementia, reported no significant trend among white men, African-American men and women due to limitation by small numbers of participants, but exceptionally among white women increasing CAC-strata significantly associated with increased risk of dementia culminated at CAC > 400 vs CACs = 0 (102vs31per1000person-years).

A cohort study by Vinter et al. [107] recruiting 28,549 participants, whom are 13,069 males with median 2.8 years of follow-up period and 15,480 females with median 2.9 years of follow-up period, to investigate association of CAC score and cancer risk, reported both genders had no significant stratification by CAC-score categories across score points of 1, 100, 400 and 1000 compared to reference CACs = 0 for the occurences of tobacco-smoking related cancer, prostate cancer, lung cancer, colorectal cancer and total cancer, moreover, relations of doubling of CAC score with cancer were modified by gender and in unadjusted model for males only with tobacco smoking related cancer risk (HR:1.05) and for females tobacco smoking related cancer risk (HR:1.04), lung cancer risk (HR:1.15) and colorectal cancer risk (HR:1.09) significantly associated, however, in adjusted model (of age as a timescale, BMI, DM, Smoking, Lipid-lowering treatment, Hypertensive treatment, Creatinine and Heart Failure) only lung cancer risk for females (HR:1.10) remained significant with doubling of CAC-score.

A cohort study by S.P. Whelton et al. [108] including 66,636 patients aged mean 54 years on median follow-up period of 12.4 years demonstrated at CAC-score < 300 cancer is the leading cause of death such as at CAC = 0 half of the mortality outcome caused by cancer, while with developing CAC-score and concurrent increases in CHD and CVD mortality rates exceed increase in cancer mortality rate and for a CAC-score ≥ 300 progression of CAC-score inversely associated with the proportion of mortality caused by cancer, moreover, compared to reference CAC = 0 at each subsequent CAC-score categories defined by score points of 1, 100 and 300, associated significantly with higher CVD, CHD and non-CVD mortality risks in unadjusted and adjusted models (for age, gender, HTN, Hyperlipidemia, smoking, diabetes and FH of CHD) except cancer mortality rate remained similar for a CAC-score < 300 in adjusted models, nonetheless a CAC ≥ 300 had significantly higher cancer mortality risk than the zero-CAC score.

## 10 Therapeutic management of SCVD

According to 2018 ACC/AHA Guideline on the management of Blood Cholesterol, statin therapy is recommended for any CAC ≥ 100 or ≥ 75 percentile [26]. Society of Cardiac Computed Tomography (SCCT) 2017 Consensus Report suggested initiation of statin allocation at non-zero CAC score and they stratified intensity of treatments with severity of score intervals among 5%-to-20% 10-year ASCVD risk group as moderate-intensity statin for CAC 1-to-99 and < 75th%, moderate-to-high intensity statin for CAC 1-to-99 and CAC ≥ 75th%, moderate-to-high intensity statin + ASA 81 mg for CAC of 100-to-299, and high intensity statin + ASA 81 mg for CAC ≥ 300 [92]. Similarly, Cardiac Society of Australia and New Zealand (CSANZ) Position Statement released on 2017 stratified preventive management, however for a CAC = 0 or CAC 1-to-100 this position statement didn’t suggest any preventive pharmacotherapy other than maintenance of healthy diet and lifestyle excepts presenting other clinical factors such as family history of premature ASCVD and comorbidities, and they suggested recommended aspirin + considerable use of statins for a score of CAC 101-to-400 where > 75th% up-titrated, and a score of CAC > 400 qualified recommended use of Aspirin and Statins with target LDL < 2.0 mmol/L [63]. SCCT consensus report and similarly CSANZ position statement both recommended repeating CAC scanning at every 3-to-5 years for a non-zero CACs > 0 yet rescanning participants with CACs = 0 at each 5-years only recommended by SCCT consensus rather than CSANZ statement [92]. Re-testing CAC can be reasoned as need to re-classify ASCVD risk on intermediate risk for titrating the treatment, evaluating efficacy of the management, guiding for intensification or modifying the preventive management, and assisting compliant patient concerning atherosclerosis and its progression [63], [67], [92].

### 10.1 Statin treatment

Nasir et al. [88] reported CAC suggesting limited role in decision of statin therapy, however, in their cohort with median follow-up period of 10.3 years including 4758 participants aged 45 to 75 evaluated with ACC/AHA guidelines reported among patients statins not recommended (with low risk ASCVD 10-year risk < 5%) event risk exceeded the statin allocation threshold of ASCVD 10-year risk > 7.5% at CACs > 100 as ASCVD event rate of 9.6 per 1000 person-year and CHD event rate of 8.9 per 1000 person-year besides for assumed 30% risk reduction associated with statin use in ASCVD-event and CHD-event 10-years NNT in statin not recommended group was estimated for both as 35, moreover, for participants considered for statin (10-year ASCVD risk 5–7.5%) any CACs > 0 had ASCVD-event rate of 7.4 per 1000 person-years at near-margin of statin recommendation and those with CACs = 0 had very low risk of ASCVD-event rate of 1.5 (per 1000 person-years), nonetheless, among participants of recommended statin (10-year ASCVD risk 7.5–20%) those with CACs = 0 had ASCVD-event rate of 5.2 per 1000 person-years still remaining within statin considered risk interval yet those with CACs 1–100 had ASCVD-event rate of 8.8 (per 1000 person-years) or among statin considered CACs 1–100 had ASCVD-event rate of 7.8 (per 1000 person-years); and in summary these findings suggest limited role to CACs for deciding statin treatment mostly achieved through up-stratification as in statin not recommended and considered groups and only down-stratification could be achieved among intermediate risk statin considered participants for those with CACs = 0 with small margin of calcification against any CACs > 0.

Similarly, Bittencourt et al. [89] in their cohort study with median follow-up of 12.2 years involving 5602 participants evaluated with ESC guidelines, presented patients with CAC > 100 among group of lipid-lowering therapy not recommend had 10-year cardiovascular mortality rate of 1.79% and 10-year CHD-event rate of 12.56% suggesting re-classification to lipid-lowering therapy recommended group, among the same treatment group at threshold of CAC > 100 10-year NNT to prevent 30% of CHD-event risk in 10-years was calculated as 27, and regardless of recommended treatment group score of CAC > 100 associated with at least intermediate risk (1–5%) level of 10-year cardiovascular mortality rate and high risk of 10-year CHD-event rate (SCORE ≥ 10%), however, they concluded re-classification due to CAC > 100 is limited and routine-use of CAC score among treatment not-recommend patients (only 6% of this subgroup) could be viable for only selected cases i.e. patients with family history of premature atherosclerosis or patients with metabolic syndrome, moreover, similar to Nasir et al. only down-stratification option was detected among considered lipid-lowering therapy by CACs = 0 yet with small-margin of calcification against CACs 1–100.

Blaha et al. [74] within their cohort study including 2083 participants aged median 67 years of age over median follow-up of 5.8 years, observed CAC > 100 at prevalence of 25% and 31% for women and men, respectively, and 75% of all CHD-events happened with a score of CAC > 100, moreover, for overall-cohort in fully-adjusted model (of age, ethnicity, HTN, smoking, BMI, HDL-c, use of antihypertensive, FH of CHD, socioeconomic status and Study site) at 5.8 years CACs > 100 vs CAC = 0 had significantly associated with CVD-event at rate of 26.4 per 1000 person-years and prevalence of 13.4% yet CACs 1–100 vs CACs = 0 had nonsignificant association with CVD-events at rate of 8.4 (per 1000 person-years) and prevalence of 4.5%, furthermore, 13.65% CVD event rate at 5.8 years occurred through Kaplan-Meier statistics with estimated NNT-5 years (with Rosuvastatin treatment, HR:0.56) for CVD as 19 or extrapolated NNT-5 years (30% CHD-event risk reduction by moderate statin) as 28 or extrapolated NNT-10 years (30% CHD-event risk reduction by moderate statin) as 17.

Mahabadi et al. [90] in their cohort study involving 3575 participants aged mean 59 ± 9 years of age with mean follow-up time of 10.4 years to observe differences in statin allocation by ESC guidelines vs AHA/ACC guidelines and rates of CVE stratified by CAC scores and statin-status seldomly and in interaction, reported statin indication according to ACC/AHA guidelines had higher rate compared to ESC guidelines (56% vs 34%) yet among participants without statin indication rate of CVD-events at 10.8 years was higher for ESC-guideline (4.0% vs 2.1%), moreover, CVD-event rates were stratified by increasing CACs-categories (of 0, 1–99, 100–399 and ≥ 400 AU) regardless of statin-indication status according to either ESC or AHA/ACC guidelines, furthermore, among participants without statin indication according to ESC and AHA/ACC guidelines CACs ≥ 100 had CVD-event rate of 8.7 (per 1000 patients-years) with NNT-10 (30% risk reduction by moderate statin use) of 38 and 6.5 with NNT-10 of 51, respectively, but participants with CACs 1–99 had low event-rate of CVD as 4.3% and 2.8%,respectively; and similar to previous studies participants with statin indication and CACs = 0 for both ESC and ACC/AHA guidelines had CVD event risk at 10-years exceeding 5% (as 5.7% and 5.4%, respectively) with small-margin of calcification against CACs > 0 or CACs 1–99 exceeding 7.5% risk level as 7.8% and 7.5%, respectively.

#### 10.1.1 Systemic review and Meta-Analysis on Statin-Initiation in Low-Risk patients and Statin-Withhold in High-Risk patients

To determine role of CACs in statin initiation to prevent events otherwise untreated and statin cessation to avoid unnecessary treatment otherwise less-benefit from therapy with inappropriate resource allocation, a systemic review and *meta*-analysis was conducted in accordance with recommendations by PRISMA-guideline [203].

##### 10.1.1.1 Search strategy

Identification of literature studies were conducted in PubMed database among studies published between 2000 and 2021. Keywords of “CAC”, “Statin”, “Statin Initiation” and “Statin Allocation” were used in different combinations with search command of “and”.

##### 10.1.1.2 Eligibility criteria

Participants were asymptomatic to any CVD, without statin or lipid-lowering medication use at baseline, and generally healthy middle-aged. Statin eligibility defined by either guideline recommendations (of ACC/AHA or ESC) or trial-based criteria or prescribed during cohorts were recognized as interventions. Stratification by CACs severity as 0, 1–100 and > 100 was used for controlling interventions. Composite outcomes of combined any CVD related events and mortality were counted as outcomes. Randomized or non-randomized cohort studies were included.

##### 10.1.1.3 Study selection and data extraction

Single author of this study (C.D. Saydam) conducted literature search, screening and subsequently data-extraction for eligible studies. Literature studies were screened with abstracts and full texts if available. Composite outcomes were standardized on 10-years of follow-up either calculated through presented event rates of per 1000 person-years or directly extracted from studies if reported.

##### 10.1.1.4 Quality assessment

Quality of included studies were assessed with using Newcastle-Ottawa Scale. Included studies were population-based cohort studies with sufficient follow-up including analysis of 4 MESA-study, 1 Framingham Heart Study, 1 Heinz-Nixdorf Recall Study and 1 Jackson Heart Study.

##### 10.1.1.5 Statistical analysis

Composite outcomes across statin eligibility groups were analyzed with using both random and fixed effects model. Der Simonian and Laird random effects model with Mantel-Haenszel method was used to calculate OR and 95% confidence interval. Heterogeneity was assessed by Cochran‘s Q statistic and I^2^-statistics on which statistically significant interstudy heterogeneity was defined as p < 0.1 by Chi-squared test (Q-statistic) and I^2^ > 50%, respectively [204]. Publication bias was assessed by Egger‘s regression test and visual examination of funnel plots. All quantitative *meta*-analyses were conducted with Revman v5.4 [205].

##### 10.1.1.6 Results

###### 10.1.1.6.1 Study population

**Fig. 1:**
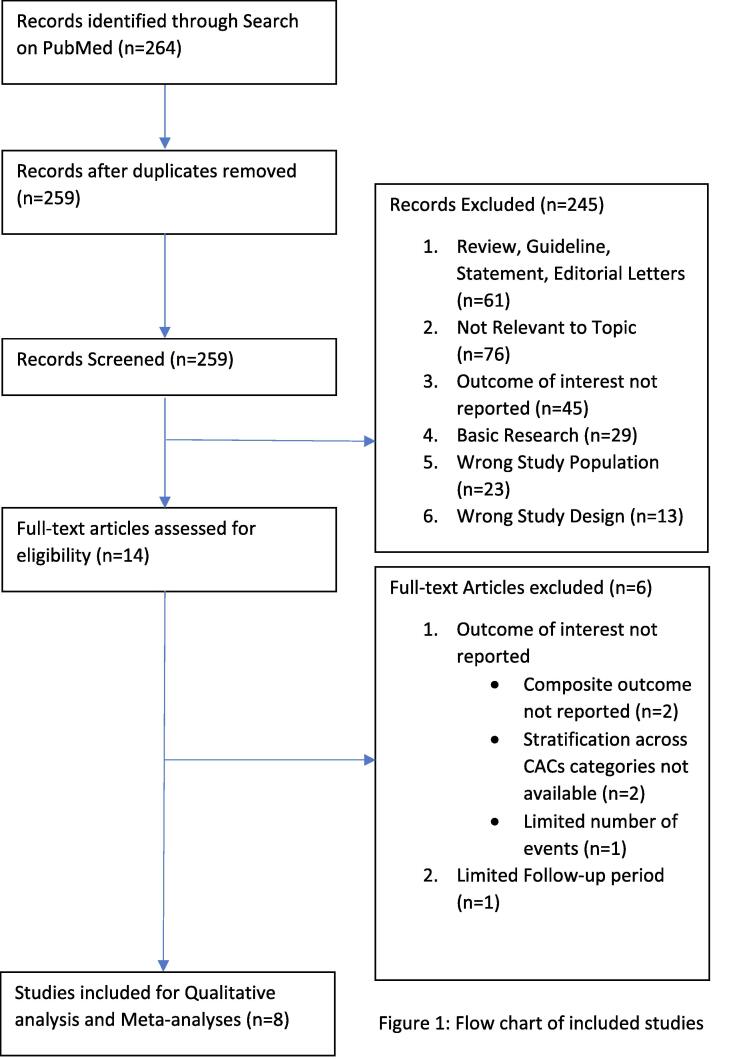
Flow chart of included studies.

As represented in Fig. 1, 264 studies were identified in literature search. After removal of 5 duplicate studies 245 records were excluded from analysis through examining title, abstract and and if available full-text for a second look. This resulted 14 potentially eligible studies and full text are retrieved. After detailed examination 8 of 14 were considered to be eligible. Included studies were all population-based analysis part of long-years of consecutive prospective cohort studies with multi-centered recruitments, secure records and variable structured interview data. Included studies were evaluated in terms of quality by Newcastle Ottawa Scale and all-included studies were scored at least 5 out of 9. Patient characteristics and study designs of included studies were presented on Table 1.

**Table 1:** Patient’s characteristics and study designs of included studies.

**Study Name**	**Study Type**	**Number**	**Mean Follow-up**	**Imaging Method**	**Age**	**Male (%)**	**Total Cholesterol**	**LDL-c**	**HTN (%)**	**DM (%)**	**Current Smokers (%)**	**BMI**
Blaha et al. [74]	Non-randomized	2083	5.8 years (Median)	EBCT	67 years (median)	60%	NA	2.64	47%	NA	12%	NA
Nasir et al. [88]	Non-randomized	4758	10.3 years (Median)	EBCT, MDCT	59 ± 9 years	47%	NA	NA	NA	NA	NA	NA
Mahabadi et al. [197]	Non-randomized	3575	10.4 ± 2.0 years	EBCT	59 ± 8 years	47%	232 ± 39	148 ± 36	NA	11.4%	23%	NA
Mitchell et al. [198]	Randomized	13,644	9.4 years (Median)	EBCT	49.6 ± 8.4 years	71%	NA	NA	NA	NA	NA	NA
Al Rifai et al. [199]	Non-randomized	6811	13.9 years (Median)	EBCT	62 years (SD = 10Y)	47%	NA	NA	NA	NA	NA	NA
Mortensen et al. [200]	Non-randomized	5600	10-years	EBCT, MDCT	61 years (Median)	47%	5.0	3.1	44%	11%	13%	NA
Pursnani et al. [201]	Non-randomized	2435	9.4 years (Median)	MDCT	51.3 years	44%	199	121	25.3%	4.1%	13.1%	NA
Shah et al. [202]	Non-randomized	2812	10-years (Median)	MDCT	55.4 years (SD:9.4)	34.7%	202.4 ± 39.5	129.6 ± 39.5	52.8%	15.4%	12.9%	31.6 (7.0)

###### 10.1.1.6.2 Across statin eligibility status stratification of ASCVD-Outcomes by CACs categories

As shown in Table 2, pooled effect of CACs > 100 vs CACs ≤ 100 among statin-eligible participants had significantly greater risk of ASCVD-outcomes with OR:5.95 and 12% difference in absolute risk yielding 15.1% calculated 10-year ASCVD outcome risk, which may up-stratify participants with CACs > 100 into statin-eligible by exceeding statin recommended thresholds of either ESC or ACC/AHA guideline. Significant interstudy heterogeneities were observed for both plots.

**Table 2:** Forest plots of pooled 4 studies examining CACs > 100 vs CACs < 100 among statin ineligible participants showing Odds Ratios **(A)** and Absolute Risk Difference **(B)** for composite outcome of ASCVD related events and mortality.

As presented in Table 3, pooled effects of CACs = 0 vs CACs > 0 among statin eligible participants could significantly decreased CVD composite outcome with OR:0.33 and 7% of absolute risk reduction resulting calculated 3.34% 10-year CVD outcome risk, which may down-stratify participants with CACs = 0 into statin ineligible even below intermediate statin considered risk level. Significant heterogeneities were observed for both plots.

**Table 3:** Forest plots of pooled 7 studies examining CACs = 0 vs CACs > 0 among statin eligible participants showing Odds Ratios **(A)** and Absolute Risk Difference **(B)** for composite outcome of ASCVD related events and mortality.

##### 10.1.1.7 Publication bias

For presented plots funnel plot examination showed symmetrical distribution, which suggest no serious publication bias.

### 10.2 Aspirin treatment

Miedema et al. [91] demonstrated for a score of CAC ≥ 100 both genders regardless of their conventionally qualified risk level by threshold 10% risk could have a favorable risk/benefit profile with aspirin treatment, in gender-specific analysis a non-zero score of CAC ≥ 1 could have a favorable profile in males regardless of risk level and females qualified low risk despite females qualified high risk with any CAC score could have favorable aspirin use profile, and in age-stratification analysis patient group aged 45-to-59 any non-zero score of CAC ≥ 1 had favorable aspirin use profile nonetheless with aging for a CAC ≥ 100 score aspirin use profile becomes less favorable as aging increases contribution of aspirin use to bleeding risk in 5-year from 0.13% to 0.46% and 0.56% in patient groups aged 60-to-69 and 70-to-84, respectively.

A cohort study by Cainzos-Achirica et al. [212] with median follow-up period of 14-years including 3540 aspirin naïve aged < 70 years and mean 56.5 (7.2) years of age selected from MESA-study population without high bleeding risk to examine guiding role of CACs in aspirin allocation through assessing rates of CVD-events and bleeding events, estimation of NNT_5_ (assumed 12% 5-years relative risk reduction-RRR or RRR of 9%) and NNH_5_ (assumed 42% 5-years relative risk increase-RRI or RRI of 37%), reported across CACs ≥ 100, CACs 1–99 and CACs = 0 CVD event rates (per 1000 person-years) stratified sequentially as 12.35 (8.41–18.14), 4.58 (2.92–7.18) and 1.39 (0.84–2.30) yet CACs categories and bleeding events had no significant association, moreover, as the baseline CACs categories increase NNT_5_ decrease and NNH_5_ increase, coherently NNT_5_ (RRR of 12%) exceeded NNH_5_ (RRI of 42%) at CACs ≥ 100 category (140vs518) and especially CACs ≥ 400 had probably highest benefit from aspirin with NNT_5_:100 yet NNH_5_ exceeded NNT_5_ for CACs 1–99 (167vs365) and CACs = 0 (567vs1190), furthermore, in sensitivity analysis assuming RRR of 9% and RRI of 37% didn‘t modify relations of NNT_5_ and NNH_5_ at CACs ≥ 100 and CACs ≥ 400, in addition, also in post hoc analysis replicating benefit/harm calculations among MESA participants with baseline aspirin use aged < 70 years without high bleeding risk NNT/NNH analyses by CACs stratification remained consistent.

A cohort study by Ajufo et al. [213] with mean follow-up period of 12.2 (1.9) years involving 2191 participants aged mean 44.4 (9.1) without already existing ASCVD and baseline aspirin use recruited from the Dallas Heart Study to investigate role of CACs in guiding aspirin allocation through ASCVD (RRI of 10%) and bleeding events (RRR of 39%), reported as CACs categories of 0, 1–99 and ≥ 100 increased rates of both ASCVD-event and bleeding event increased but after multivariable adjustment association between CACs category and bleeding event attenuated, however, in sensitivity analysis when participants were further stratified by bleeding risk (as higher and lower) and PCE risk categories (as < 5%, 5–20% and > 20%) among participants with lower bleeding risk for intermediate risk PCE 5–20% at CACs ≥ 100 ASCVD risk reduction exceeded bleeding risk but not for low risk PCE < 5% (regardless of CACs categories) and for high risk PCE > 20% aspirin allocation had net benefit of ASCVD prevention over bleeding regardless of CACs categories, however, participants with higher bleeding risk regardless of PCE levels and CACs categories had net harm of bleeding over ASCVD prevention.

### 10.3 Hypertension treatment

A cohort study by McEvoy et al. [214] with median follow-up period of 10.2 years including 3733 MESA participants with SBP 120–180 mm Hg to examine role of CACs in detecting prevention candidate (with assumption of per 10 mm Hg SBP reduction prevents 22% of CHD, 41% of stroke and 24% of HF) and intensification of antihypertensive therapy targeting SBP goal 130 up to 120 mm Hg through comparing ASCVD event rates and NNT_10_ (with target SBP of 120), reported participants with SBP 120–139 mm Hg and without BP-treatment at CACs > 100 had similar rate of ASCVD-event with participants on SBP 160–179 mm Hg with BP-treatment at CACs = 0 (as 24.3 and 20.2 per 1000 person-years, respectively) and for participants on SBP 160–179 mm Hg ASCVD-event rates remained similar with CACs categories (0, 1–100 and > 100) regardless of receiving BP-treatment but for those on SBP < 140 mm Hg and SBP 140–159 mm Hg compared to CACs = 0 ASCVD-event rates significantly incrementally increased with CACs categories, similarly also in stratification of SBP levels (<140, 140–159 and 160–79) by dichotomy by ASCVD risk threshold PCE ≥ 15% participants on SBP < 140 mm Hg and SBP 140–159 mm Hg but not SBP 160–179 mm Hg had significant association between CACs-tertiles and ASCVD events rates, moreover, these increased relative risks with upper CACs strata were more distinct for either BP-untreated vs treated or low PCE-risk < 15% vs ≥ 15%, furthermore, in NNT_10_ analysis participants with CACs > 100 had lower NNT rates compared to lower tertiles especially for ASCVD < 15% at which participants on SBP < 140 with ASCVD risk ≥ 15% and CACs > 100 had comparable NNT_10_ with SBP 140–159*ASCVD ≥ 15%*CACs = 0 and SBP 160–179*ASCVD < 15%*CACs 1–100 (NNT of 15, 15 and 18 respectively) likewise participants on SBP < 140 with ASCVD risk < 15% and CACs > 100 had considerable NNT_10_ with SBP 160–179*ASCVD < 15%*CACs = 0 (24and20,respectively), and consistently both SBP 140–159*ASCVD < 15%*CACs > 100 and SBP 160–179*ASCVD ≥ 15%*CACs = 0 had NNT_10_:5, coherently at all SBP levels NNT10 had widest estimates across CACs tertiles for those with ASCVD < 15% especially most distinctly on SBP < 140 as 99, 52 and 24, respectively.

### 10.4 CAC-Scanning and outcomes of treatment behaviors for SCVD

Greenland et al. [118] reviewed a randomized study comparing outcomes of 4-years follow-up of participants with CAC scanning versus participants without CAC scanning, and in CAC screening more favorable improvement in blood pressure, LDL and waist circumference, and lower FRS-score had been reported, moreover, Greenland et al. also presented a *meta*-analysis result showing a CAC > 0 compared to CAC = 0 had associated with higher rates of aspirin, lipid-lowering and antihypertensive drug initiations, higher continuation or patient compliance with lipid-lowering drug, increased exercise and dietary modifications.

An observational retrospective follow-up study by Kalia et al. [215] including 2608 individuals aged mean 58 ± 8 years with mean 4.1 ± 3.2 years of interscan period to examine effects of CAC imaging and patient knowledge of CACs burden on self-reported patient adherence to lipid-lowering treatment and weight loss, presented statin compliance increased gradually with higher CACs categories (0, 1–100, 100–400 and > 400) compared to CACs = 0 in logistic regression model for both univariate and adjusted model (of age, gender and ethnicity), which revealed OR:1.6, OR:2.7 and OR:3.1 at each subsequent quartile, however, in subgroup analysis for weight loss involving 1078 participants aged mean 60 ± 8 years univariate model but not adjusted model showed significant association at only CACs 100–400 (OR:1.8) and CACs > 400 (OR:2.1).

An open-label, multi-center randomized controlled trial by Venkataraman et al. [216] including 214 CAUGHT-CAD trial participants with family history of premature CAD and non-zero CACs 1–400 per arm of trial investigating influence of CAC imaging on patient and physician behavior at which participants of experiment group or themed “CAC-guided arm” received information of their CACs and CT image of their coronary artery calcification with encouragement of displaying on daily and participants of control group or themed “control arm” besides their physicians were blinded from their CAC results along 12 months of follow-up assessment but all participants were revealed with presence of a non-zero CACs 1–400, reported 1% (95% CI: 0.13–1.81) absolute risk reduction in CAC-guided arm and 0.43% (95% CI: 0.11–0.75) absolute risk increase in control-arm cumulatively 1.47% absolute risk difference equivalent to RRR of 18% without significant gender difference and psychosocial or sociodemographic factors, moreover, on intention-to-treat analysis CAC-guided arm had significant reductions at 12-month outcomes in TC of 1.52 mmol/L or 28% and LDL-c of 1.39 mmol/L or 26% from baseline examination with noted limitation of 11% imputation in data yet control-arm group had no significant change besides HDL-c remained similar across groups along with SBP but conducing 14% of the absolute PCE risk reduction of 1.47%, however, self-reported lifestyle behaviors of low salt diet, achieving > 5% weight loss, doing regular physical activity and prevalence of current smoking and alcohol use remained similar across groups.

## 11 Cost effectiveness of CAC imaging

A Markov modelling by E. T. Roberts et al. [208] for outcomes of CHD and composite CVD-events involving 2000 hypothetical participants in prospective cohort study of MESA regarding data on intermediate risk population by ATP-3 FRS (6–20%) across 5- or 10-year time horizon of model running until absorbing state of first CHD or CHV event causing loss of utility, to investigate clinical outcomes and cost-effectiveness (according to 2007 Medicare Advantage data inflated to 2011 dollars with %3 annual discount in model-run at all costs and outcomes) of CAC study valued through incremental effectiveness of averting first CVD-events and utility decrement of QALY also with counting QALY of statin complications, patient preferences for statin use, opportunity cost of 1 h test-duration on US median hourly wage, side-effects of CAC screening, in risk stratification and treatment allocation (of statin under assumptions of risk reduction as 25–45% for moderate intensity and 35–55% for high intensity and treatment adherence of 55% and 65% for ATP-3 and CAC strategies, respectively), and testing 3 strategies of statin initiation among intermediate risk population as treat-all, treatment according to ATP-3 and CACs for a CACs ≥ 1 or CACs ≥ 100 in pairwise comparison of incremental costs and incremental effects termed ICER per strategies as choosing CAC strategy or most favorable strategy with noted ICER value if ICER<$50000 otherwise treated with ATP-3, reported in terms of preventing events as outcomes treating patients with CACs ≥ 0 was more effective than treat-all (as reduction of CVD events 9.8 per 1000 patients vs 8.6 events) and screening all intermediate risk participants besides treating those with CACs ≥ 1 was more cost effective compared to treat all and ATP-3 strategies until CAC screening remaining <$235/test or base-case analysis assuming costs of $100 for CAC testing and $180 for annual statin use, however, in terms of gaining QALY as outcomes at non-zero willingness-to-pay threshold>$0/QALY treating patients with CACs ≥ 100 induced greater improvement in QALY compared to strategies of treat-all, ATP-3 and CACS ≥ 1 besides reducing negative side effects of statin through withholding statin for participants with non-zero CACs < 100 in base-case simulations and these results didn‘t change after involving indirect costs and costs of re-examinations due to incidentalomas even after adjusting event rates into 2 times of MESA case set.

A microsimulation model by J. C. Hong et al. [209] estimating clinical and economic outcomes of ASCVD for participants at intermediate 10-year risk by ACC/AHA (5–7.5%) or classified as statin considered recruited from MESA study to evaluate cost-effectiveness of CAC screening (according to Medicare insurance claim data and all costs were reported in 2016 U.S. dollars) through incrementally comparing 2 strategies of statin allocation as either treat-all with moderate intensity statin (with assumed 55% treatment adherence) or CAC-strategy titrating statin with CACs categories (with assumed 65% treatment adherence) as no statin, moderate intensity statin (assumed mean 35% risk reduction) and high intensity statin (assumed mean 45% risk reduction) across CACs = 0, CACs 1–100 and CACs ≥ 100, reported in base-case assumptions (of cost for CAC testing $200 but in addition to direct cost of testing includes extra physician visit, further examinations of incidental findings and hourly wage cost during imaging, and Annual Statin use $85) among participants with statin considered CAC strategy had greater cost-effectiveness than treat-all strategy and at any non-zero willingness-to-pay thresholds CAC strategy was most cost-effective strategy, moreover, in sensitivity analysis of varying assumptions if annual cost of statin increase with becoming ≥$150/year CAC strategy dominated and if CAC testing cost increase with >$250 treat-all strategy became more favorable, furthermore, for increased disutility with statin therapy trading either 4 or 8 weeks of life for 10 years of not taking a statin rather than no disutility (treat-all: $7333/QALY), statin follow-up at 6 months, unrelated health care costs and payer perspective CAC strategy dominated yet for 10-year time horizon (treat-all: $9000/QALY) and same moderate intensity for both strategies ($5222/QALY) treat-all had more favorable cost-effectiveness.

A microsimulation model by Spahillari et al. [210] evaluating cost-effectiveness and clinical outcomes of CAC strategy (non-zero CACs > 0) in statin allocation according to costs derived from health care sector perspective modified to 2017 US Dollars among 472 African American individuals of prospective community based Jackson Heart Study (JHS) at intermediate risk eligible for statin therapy through incrementally comparing ACC/AHA 2013 guideline without strong recommendation for CAC testing vs ACC/AHA 2018 guideline with recommendation of CACs assessment by ICER, reported statin indication by 2013 guideline had greater rate of statin indication (67% vs 39%), lower ASCVD-event rate (26.86% vs 27.54%) but higher rate of statin related adverse effects (3.14% vs 1.84%), on base-case analysis (assuming $183 for cost of CAC and $84 for cost of annual statin use) of QOL by probabilistic sensitivity analysis using Monte Carlo simulations showing probability of cost-effectiveness on curves of willingness-to-pay values 2018 guideline had greater cost-effectiveness than 2013 guideline and despite 2013 guideline showing greater gain in per person QALY (ΔQALY = 0.0027) 2018 guideline dominated 2013 without CAC assessment in ICER ($11544/QALY vs. $158325/QALY) with 83% of difference in base-case QALY (0.0027 vs 0.016), increased adherence to statin treatment due to CAC testing and difference of QOL penalty (0.996 of 1 ideal vs. none), and for high non-zero CACs prevalence varying 40–80% from 58.6% of base-case value modified 2018 guideline dominating 2013 with ICER of $53993/QALY.

A microsimulation model by Venkataraman et al. [211] including asymptomatic 1083 participants of CAUGHT-CAD trial (Coronary Artery calcium score use to guide management of Hereditary CAD) with FHCAD (Family history of premature coronary artery disease) at intermediate risk for evaluating cost-effectiveness of CAC-guided strategy in statin initiation in terms of improvement in QALY with direct medical costs (CAC testing, examinations of incidental findings, increased life-time risk of radiation-induced cancer) through incrementally comparing with standard care model indication by PCE 10-year risk (≥7.5%) on ICER index at which all model costs were in 2020 USD consumer price besides including 1-time utility decrement of statin disutility and statin related complications into base-case model, reported in base-case analysis CAC strategy (CACs > 0) vs PCE ≥ 7.5% had significantly higher cost-effectiveness of ICER ($15014), average cost ($6059 vs $5914, Δ:$145) and person-years of statin therapy (794482vs342592) yet lower than treat-all strategy, greater number of major statin complications (5405vs1945), lesser aversion of person-years with symptomatic CVD, prevented 1314 more CVD-events and 476 deaths, and had NNS (to prevent 1 CVD event) of 152, moreover, according to sensitivity analysis at WTP=$50000 CAC strategy was cost-effective for baseline PCE 5.0–7.5% and cost-saving for baseline PCE ≥ 7.5% but not cost-effective for low risk PCE < 5%, furthermore, addition of aspirin to statin treatment for those with CACs ≥ 100 increased cost $63 (to $6005) and QALY 0.0069 (to 9.3932) but prevented 462 deaths and improved ICER to $8998/QALY and $27271/Life saved, moreover, if statin initiation in CAC strategies were increased to CACs ≥ 100 and 10 year MESA risk ≥ 5% ICER improved to $7000/QALY.

A retrospective study by Patel et al. [227] including 82,214 patients recruited from SHAPE (The society for heart attack prevention and eradication) cohort study conducted in 7 different countries (US, Malta, Australia, Portugal, Turkey, France and UAE) and 33 different medical centers reported among entire cohort mean radiation dose 1.03 mSv (0.76–1.31 mSv) and median dose 0.94 mSv both are far below average US national background radiation exposure of 3.0–3.6 mSv.

## 12 Limitations and conclusions

In this review study discussion of comparing novel risk markers for measuring SCVD was limited by ESC and AHA guidelines suggested markers of CAC-score, CIMT, ABI, hs-CRP, FMD and aPWV. In literature CAC-score has been established with its superior re-classification performance among novel risk markers, so studies evaluating SCVD with CACs were mainly selected. In this review, interpretation and reporting standards of CAC-score based on Agatston score and it‘s demographic distribution were well clarified.

CAC development and its associated progressing factors were deliberately reviewed. However, as a limitation of this study because of sparsity of randomized controlled trials causal relations in CACs progression were mainly visited with cohort, follow-up and case-control studies. Quality assessment per included studies was also conducted. However, intrinsic confounders of reviewed could be still residual.

As a strength of this study roles of CAC imaging and its progressing factors were reviewed comprehensively with detailed broad literature search, covering high number of original studies, consecutive reports, relevant reports and studies having robust design, objective assessments, well characterized methodology, large study populations, multicentric studies, multi-ethnic/racial studies, well-characterized study population. As another strength of this review study, to determine the role of CAC imaging in statin allocation summary estimates of published studies as a systemic review and *meta*-analysis were conducted in accordance with recommendations by PRISMA-guideline.

As a conclusion, CAC score can be utilized at any age of adulthood as an useful tool for periodically assessing SCVD with low hazard of radiation and cost-effective at any intermediate risk patients, detecting an unfavorable changes from zero-baseline or across CAC score strata and deteriorating atherosclerosis subclinically, evaluating effectiveness of life-style modifications and preventive efforts within cumulative periods, guiding informed decision making of patients, adjusting treatment allocation, recalibrating risk estimations, motivating patients’ efforts to adhere treatment plans and healthy behavior changes, promoting self-discipline of persisting healthy behaviors and awareness of healthy life-styles, and gauging patients’ behaviors in cardiovascular health dimensions.

As final remarks, a CACs > 100 may suggest statin indication otherwise ineligible before test and a CACs = 0 may suggest statin withhold otherwise statin eligible before test. Physical activity, dietary factors, cigarette use, alcohol consumption, metabolic health, family history of CHD, aging, exposures of neighborhood environment and noncardiovascular comorbidities can determine CACs changes.

## Declaration of Competing Interest

The authors declare that they have no known competing financial interests or personal relationships that could have appeared to influence the work reported in this paper.

## References

[1] Goff D.C. (2013). ACC/AHA Guideline on the Assessment of Cardiovascular Risk A report of the ACA/AHA Task Force on Practice Guideline. The Journal of the American College of Cardiology.

[2] Piepoli M.F. (2016). European Guidelines on Cardiovascular disease prevention in clinical practice. Eur. Heart J..

[3] Flice R. (2007). Subclinical Disease Detection Advanced Imaging Applications. Top. Magn. Reson. Imaging.

[4] Mitu O. (2017). Subclinical cardiovascular disease assessment and its relationship with cardiovascular risk in SCORE in a healthy adult population: A cross sectional communit based study. Clinical Investigation of Arteriosclerosis.

[5] Peters S.A.E. (2011). Improvements in risk stratification for the occurence of cardiovascular disease by imaging subclinical atherosclerosis: a systemic review. Heart.

[6] van Werkhoven J.M. (2009). Multislice computed tomography coronary angiography for risk stratification in patients with an intermediate pretest likelihood. Heart.

[7] Arthur P. (2009). DeMarzo, Using impedance cardiography to detect subclinical cardiovascular disease in women with multiple risk factors: A pilot study. Preventive Cardiology.

[8] Polonsky T.S. (2017). Association of Cardiovascular Health with Subclinical Disease and Incident Events: The Multi Ethnic Study of Atherosclerosis. Journal of American Heart Association.

[9] Chillaron J.J. (2014). Subclinical cardiovascular disease in type 2 diabetes mellitus: To screen or not to screen. World Journal of Clinical Cases.

[10] Bos D. (2018). Leveraging the coronary calcium scan beyond the coronary calcium score. Eur. Radiol..

[11] Ekblom-Bak E., Ekblom Ö. (2017). Fitness attenuates the prevalence of increased coronary artery calcium in individuals with metabolic syndrome. European Journal of Preventive Cardiology.

[12] Shpilsky D. (2018). Association between ideal cardiovascular health and markers of subclinical cardiovascular disease. Clin. Cardiol..

[13] Patel J. (2015). Coronary Artery Calcium Improves Risk Assessment in Adults with a Family History of Premature Coronary Heart Disease: Results from MESA, Circulation. Cardiovascular Imaging.

[14] Sadasivam K. (2015). Carotid Artery Intima-Media Thickness in Young Adults with Family History of Coronary Artery Disease. Journal of Clinical and Diagnostic Research.

[15] Bild D.E. (2002). Multi-Ethnic Study of Atherosclerosis: Objectives and Design. Am. J. Epidemiol..

[16] Newman A.B. (2001). Associations of Subclinical Cardiovascular Disease with Frailty. Journal of Gerontology.

[17] Newman A.B. (2003). “Successful Aging”: Effect of Subclinical Cardiovascular Disease. Arch. Intern. Med..

[18] Donald M. Lloyd-Jones et al., Defining and Setting National Goals for Cardiovascular Health Promotion and Disease Reduction: The American Heart Association’s Strategic Impact Goal Through 2020 and Beyond, Circulation, 2010, 10.1161/CIRCULATIONAHA.109.192703. 10.1161/CIRCULATIONAHA.109.192703 20089546

[19] Mark A. Hlatky et al., Criteria for Evaluation of Novel Markers of Cardiovascular Risk: A Scientific Statement From the American Heart Association, Circulation, 2009, 10.1161/CIRCULATIONAHA.109.192278. 10.1161/CIRCULATIONAHA.109.192278 PMC2956982 19364974

[20] John Bosomworth N. (2011). Practical use of the Framingham risk score in primary prevention: Canadian Perspective. Can. Fam. Physician.

[21] Zaid Maryam (2017). Coronary Artery Calcium and Carotid Artery Intima Media Thickness and Plaque: Clinical Use in Need of Clarification. Journal of Atherosclerosis and Thrombosis.

[22] Paul Wadwa R. (2007). Noninvasive measures of cardiovascular changes in diabetes mellitus; Current Opinion in Endocrinology. Diabetes and Obesity.

[23] Warraich H.J., Nasir K. (2013). Subclinical Cardiovascular Disease Assessment in Persons with Diabetes. Current Cardiology Report.

[24] Emelia J. Benjamin et al., Heart Disease and Stroke Statistics 2019 Update: A Report From the American Heart Association, Circulation, 2019, 10.1161/CIR.0000000000000659. 10.1161/CIR.0000000000000659 30700139

[25] Xanthakis (2014). Ideal Cardiovascular Health: Associations with Biomarkers and Subclinical Disease, and Impact on Incidence of Cardiovascular Disease in the Framingham Offspring Study. Circulation.

[26] Grundy Scott M. (2019). 2018 AHA/ACC/AACVPR/AAPA/ABC/ACPM/ADA/AGS/APhA/ASPC/NLA/PCNA Guideline on the Management of Blood Cholesterol: A Report of the American College of Cardiology/American Heart Association Task Force on Clinical Practice Guidelines. Circulation.

[27] Mark D. Huffman et al., Cardiovascular Health Behavior and Health Factor Changes (1988-2008) and projections to 2020 : Results From the National Health and Nutrition Examination Surveys, Circulation, 2012, 10.1161/CIRCULATIONAHA.111.070722/_/DC1. 10.1161/CIRCULATIONAHA.111.070722 PMC3914399 22547667

[28] Gilman Matthew W. (2015). Primordial Prevention of Cardiovascular Disease. Circulation.

[29] Otsuka Fumiyuki (2014). Has Our Understanding of Calcification in Human Coronary Atherosclerosis Progressed, Atherosclerosis. Thrombosis and Vascular Biology.

[30] Agatston Arthur S. (1990). Quantification of Coronary Artery Calcium Using Ultrafast Computed Tomography. Journal of American College of Cardiology.

[31] Jeffrey Carr J. (2005). Calcified Coronary Artery Plaque Measurement with Cardiac CT in a Population based Studies: Standardized Protocol of Multi-Ethnic Study of Atherosclerosis (MESA) and Coronary Artery Risk Development in Young Adults (CARDIA) Study. Radiology.

[32] Vedanthan Rajesh (2014). Bio-Imaging and Subclinical Cardiovascular Disease in Low and Middle-Income Countries. Journal of Cardiovascular Translational Research.

[33] Stein James H. (2008). Use of Carotid Ultrasound to Identify SubclinicalVascular Disease and Evaluate CardiovascularDisease Risk: A Consensus Statement from theAmerican Society of EchocardiographyCarotid Intima-Media Thickness Task ForceEndorsed by the Society for Vascular Medicine. J. Am. Soc. Echocardiogr..

[34] Tae Ho Park (2016). Evaluation of Carotid Plaque Using Ultrasound Imaging. Journal of Cardiovascular Ultrasound.

[35] Vlachopoulos Charalambos (2009). Prediction of Cardiovascular Events and All-Cause Mortality With Arterial Stiffness: A Systematic Review and Meta-Analysis.

[36] Loria Catherine M. (2007). Early Adult Risk Factor Levels and Subsequent Coronary Artery Calcification. J. Am. Coll. Cardiol..

[37] Hwang Shih-Jen (2018). Maintenance of Ideal Cardiovascular Health and Coronary Artery Calcification Progression in Low Risk Men and Women in the Framingham Heart Study, Circulation. Cardiovascular Imaging.

[38] Neves P.O., Andrade J., Monçao H. (2017). Coronary Artery Calcium Score: Current Status; Radiol Bras..

[39] Nasir Khurram, Clouse Melvin (2012). Role of Nonenhanced Multidetector CT Coronary Artery Calcium Testing in Asymptomatic and Symptomatic Individuals. Radiology.

[40] McCollough Cynthia H. (2007). Coronary Artery Calcium: A Multi-Institutional Standard for Quantification at Cardiac CT. Radiology.

[41] Blaha Michael J. (2017; 10.1016/j.jcmg.2017.05.007.). Coronary Artery Calcium Scoring: Is it a Time for a Change in Methodology; Journal of American College of Cardiology. Cardiovascular Imaging.

[42] Callister (1998). Coronary artery disease: Improved reproducibility of calcium scoring with an electron beam CT volumetric method. Radiology.

[43] Alluri (2015). Scoring of coronary artery calcium scans: History, assumptions, current limitations, and future directions. Atherosclerosis.

[44] Lo-Kioeng-Shioe (2019). Coronary Calcium Characteristics as Predictors of Major Adverse Cardiac Events in Symptomatic Patients: Insights From the CORE320 Multinational Study. Journal of the American Heart Association.

[45] Hoffmann Udo (2006). Evidence for lower variability of coronary artery calcium mineral mass measurements by multi-detector computed tomography in a community-based cohort- Consequences for progression studies. Eur. J. Radiol..

[46] Budoff Matthew J. (2018). Ten-year association of coronary artery calcium with atherosclerotic cardiovascular disease (ASCVD) events: the multi-ethnic study of atherosclerosis (MESA). Eur. Heart J..

[47] Criqui Michael H. (2017). Coronary Artery Calcium Volume and Density, Potential Interactions and Overall Predictive Value: The Multi-Ethnic Study of Atherosclerosis. JACC: Cardiovascular Imaging.

[48] Thomas Isac C. (2017). The evolving view of coronary artery calcium and cardiovascular disease risk. Clin. Cardiol..

[49] Thomas Isaac C. (2018). Association of Cardiovascular Disease Risk Factors with Coronary Artery Calcium Volume versus Density. Heart.

[50] Leening (2014). Net Reclassification Improvement: Computation, Interpretation, and Controversies. Ann. Intern. Med..

[51] Bertoni Alain G (2016). Diabetes: Insights from the Multi-Ethnic Study of Atherosclerosis, Glob. Heart.

[52] McClelland Robyn L. (2006). Distribution of Coronary Artery Calcium by Race, Gender, and Age: Results from the Multi-Ethnic Study of Atherosclerosis (MESA). Circulation.

[53] Detrano Robert (2008). Coronary Calcium as a Predictor of Coronary Eevents in Four Racial or Ethnic Groups. The New England Journal of Medicine.

[54] Laddu Deepika R. (2017). 25-year Physical Activity Trajectories and Development of Subclinical Coronary Artery Disease as Measured by Coronary Artery Calcium: The CARDIA Study. Mayo Clin Proc..

[55] Yeboah Joseph (2012). Comparison of Novel Risk Markers for Improvement in Cardiovascular Risk Assessment in Intermediate Risk Individuals, The Multi Ethnic Study of Atherosclerosis. JAMA.

[56] Yeboah Joseph (2014). Mediation of Cardiovascular Risk Factor Effects Through Subclinical Vascular Disease: The Multi-Ethnic Study of Atherosclerosis. Arterioscler Thromb Vasc Biol.

[57] Stein C. (2017). Structural Equation Modeling. Statistical Human Genetics.

[58] Polonsky (2010). Coronary Artery Calcium Score and Risk Classification for Coronary Heart Disease Prediction: The Multi Ethnic Study of Atherosclerosis. JAMA.

[59] Valenti Valentina (2015). A 15-year warranty period for asymptomatic individuals without coronary artery calcium: a prospective follow-up of 9715 individuals. JACC Cardiovasc Imaging.

[60] Shah Ravi V. (2016). Association of Fitness in Young Adulthood With Survival and Cardiovaascular Risk The Coronary Artery Risk Development in Young Adults (CARDIA) Study. JAMA Intern Med.

[61] Peter S.A. (2012). Improvements in risk stratification for the occurence of cardiovascular disease by imaging subclinical atherosclerosis: a systemic review. Heart.

[62] Budoff Matthew J (2009). Coronary calcium predicts events better with absolute calcium scores than age-gender-race percentiles- The Multi-Ethnic Study of Atherosclerosis (MESA). J Am Coll Cardiol.

[63] Liew Gary (2017). Cardiac Society of Australia and New Zealand Position Statement: Coronary Artery Calcium Scoring, Heart. Lung and Circulation.

[64] Hoffmann (2008). Defining Normal Distribution of Coronary Artery Calcium in Women and Men from the Framingham Heart Study. Am J Cardiol..

[65] Hecht Harvey S. (2016). 2016 SCCT/STR Guidelines for coronary artery calcium scoring of noncontrast noncardiac chest CT scans: A report of the Society of Cardiovascular Computed Tomography and Society of Thoracic Radiology. J. Cardiovasc. Comput. Tomogr..

[66] Dudum Ramzi (2019). Coronary artery calcium scoring in low risk patients with family history of coronary heart disease: Validation of the SCCT guideline approach in the coronary artery calcium consortium. J. Cardiovasc. Comput. Tomogr..

[67] Rifai Mahmoud Al (2018). Coronary Artery Calcium: Recommendations for Risk Assessment in Cardiovascular Prevention Guidelines. Curr Treat Options Cardio Med.

[68] (2017). JACC: Cardiovascular Imaging.

[69] Kronmal Richard A. (2007). Risk factors for the Progression of Coronary Artery Calcification in Asymptomatic Subjects, Results from the MESA. Circulation.

[70] Tian Hu. (2019). Low-carbohydrate diets and prevalence, incidence and progression of coronary artery calcium in the Multi-Ethnic Study of Atherosclerosis (MESA). Br. J. Nutr..

[71] Sung Ki-Chul (2014). Composition of Dietary Macronutrient Intake is Not Associated with Prevalence of Coronary Artery Calcification in Healthy Korean Adults. Ann Nutr Metab.

[72] Miranda Andreia M. (2018). Coffe Consumption and Coronary Artery Calcium Score: Cross-Sectional Results of ELSA-Brasil (Brazilian Longitudinal Study of Adult Health). J Am Heart Assoc..

[73] Kianoush Sina (2017). Association of Cigarette Smoking with Subclinical Inflammation and Atherosclerosis: ELSA-Brasil (The Brazilian Longitudinal Study of Adult Health). Journal of the American Heart Association.

[74] Blaha Michael J. (2011). Association between hsCRP≥2, Coronary Artery Calcium, and Cardiovascular Events- Implications for the JUPITER Population: Multi-Ethnic Study of Atherosclerosis (MESA). Lancet.

[75] McEvoy John W (2015). The Relationship of Cigarette Smoking with Inflammation and Subclinical Vascular Disease: The Multi-Ethnic Study of Atherosclerosis. Arterioscler Thromb Vasc Biol..

[76] McClelland Robyn L. (2008). Alcohol and coronary artery calcium prevalence, incidence and progression: results from the Multi-Ethnic Study of Atherosclerosis (MESA). Am J Clin Nutr..

[77] Ogunmoroti Oluseye (2019). Alcohol and ideal cardiovascular health: The Multi-Ethnic Study of Atherosclerosis. Clin Cardiol.

[78] Brenner Allison B. (2015). Associations of Alcohol Availability and Neighborhood Socioeconomic Characteristics with Drinking: Cross-Sectional Results From the Multi-Ethnic Study of Atherosclerosis (MESA). Subst Use Misuse.

[79] Brenner Allison B. (2015). Longitudinal associations of neighborhood socioeconomic characteristics and alcohol availability on drinking: Results from the Multi-ethnic of Study of Atherosclerosis (MESA). Soc Sci Med..

[80] Sponholtz Todd R. (2019). Association of Variability in Body Mass Index and Metabolic Health With Cardiometabolic Disease Risk. Journal of the Amerian Heart Association.

[81] Roberson Lara (2014). The prevalence of the metabolically healthy obese phenotypes in an aging population and it’s association with subclinical cardiovascular disease: The Brazilian study on healthy aging. Diabetology&Metabolic Syndrome.

[82] Roberson Lara L, Beyond B.M.I. (2014). The “Metabolically healthy obese phenotype & its association with clinical/subclinical cardiovascular disease and all-cause mortality – a systemic review. BMC public Health.

[83] Susan A. (2018). Subclinical Cardiovascular Disease and Changes in Self Reported Mobility: Multi-Ethnic Study of Atherosclerosis. Journals of Gerontology: Medical Sciences.

[84] Kowall B. (2018). Associations of metabolically healthy obesity with prevalence and progression of coronary artery calcification: Results from the Heinz Nixdorf Recall Cohort Study. Nutrition, Metabolism & Cardiovascular Diseases.

[85] Alberti K.G.M.M. (2009). Harmonizing the Metabolic Syndrome: A Joint Interim Statement of the International Diabetes Federation Task Force on Epidemiology and Prevention; National Heart, Lung, and Blood Institute; American Heart Association; World Heart Federation; International Atherosclerosis Society; and International Association for the Study of Obesity. Circulation.

[86] Mamudu Hadii M (2018). Diabetes, subclinical atherosclerosis and multiple cardiovascular risk factors in hard-to-reach asymptomatic patients. Diabetes & Vascular Disease Research.

[87] Christine Paul J. (2015). Longitudinal Associations Between Neighborhood Physical and Social Environments and Incident Type 2 Diabetes Mellitus: The Multi-Ethnic Study of Atherosclerosis (MESA). JAMA Intern Med..

[88] Nasir (2015). Implications of Coronary Artery Calcium Testing Among Statin Candidates. J. Am. Coll. Cardiol..

[89] Bittencourt et al., Implications of coronary artery calcium testing on risk stratification for lipid-lowering therapy according to the 2016 European Society of Cardiology recommendations: The MESA study, European Journal of Preventive Cardiology, 2018, 10.1177/2047487318788930. 10.1177/2047487318788930 30043629

[90] Mahabadi Amir (2016). CAC Score Improves Coronary and CV Risk Assessment Above Statin Indication by ESC and AHA/ACC Primary Prevention Guidelines. JACC: CARDIOVASCULAR IMAGING.

[91] Miedema Michael D. (2014). Use of Coronary Artery Calcium Testing to Guide Aspirin Utilization for Primary Prevention: Estimates From the Multi-Ethnic Study of Atherosclerosis. Circ Cardiovascular Qual Outcomes.

[92] Hecht (2017). Clinical indications for coronary artery calcium scoring in asymptomatic patients: Expert consensus statement from the Society of Cardiovascular Computed Tomography. Harvey Journal of Cardiovascular Computed Tomography.

[93] Chun Sohyun (2015). Sugar-sweetened Carbonated Beverage Consumption and Coronary Artery Calcification in Asymptomatic Men and Women. Am. Heart J..

[94] Sekikawa Akira (2019). Association of blood levels of marine omega-3 fatty acids with coronary calcification and calcium density in Japanese men. Eur. J. Clin. Nutr..

[95] Gripeteg Lena (2018). Concomitant Associations of Healthy Food Intake and Cardiorespiratory Fitness with Coronary Artery Calcium. The American Journal of Cardiology.

[96] Talaei Mohammad (2019). DASH Dietary Pattern, Mediation by Mineral Intakes, and the Risk of Coronary Artery Disease and Stroke Mortality. Journal of the American Heart Association.

[97] Andre R. M. Paixao et al., Coronary Artery Calcification and Family History of Myocardial Infarction in the Dallas Heart Study, JACC: Cardiovascular Imaging, 2014, 10.1016/j.jcmg.2014.04.004. 10.1016/j.jcmg.2014.04.004 24954461

[98] Cohen Randy (2014). Significance of a Positive Family History for Coronary Heart Disease in Patients with a Zero Coronary Artery Calcium Score. Am J Cardiol.

[99] Won Ki-Bum (2018). Impact of optimal glycemic control on the progression of coronary artery calcification in asymptomatic patients with diabetes. Int. J. Cardiol..

[100] Dragano N. (2009). Traffic Exposure and subclinical cardiovascular disease: is the association modified by socioeconomic characteristics of individuals and neighbourhoods? Results from a multilevel study in an urban region. Occup Environ Med..

[101] Jones Miranda R. (2016). Secondhand Smoke Exposure and Subclinical Cardiovascular Disease. Journal of the American Heart Association.

[102] Tsao Tsung-Ming (2014). The Health Effects of a Forest Environment on Subclinical Cardiovascular Disease and Health-Related Quality of Life. PLoS ONE.

[103] Wang Meng (2019). Association of Estimated Long-Term Exposure to Air Pollution and Traffic Proximity With a Marker for Coronary Atherosclerosis in a Nationwide Study in China. JAMA Network Open.

[104] Dzaye Omar (2019). Validation of the Coronary Artery Calcium Data and Reporting System (CAC-DRS): Dual importance of CAC score and CAC distribution from the Coronary Artery Calcium (CAC) consortium. J. Cardiovasc. Comput. Tomogr..

[105] Ohmoto-Sekine Yuki (2016). Prevalence and distribution of coronary calcium in asymptomatic japanese subjects in lung cancer screening computed tomography. J. Cardiol..

[106] Handy Catherine E. (2016). The association of coronary artery calcium with non-cardiovascular disease from the Multi-Ethnic Study of Atherosclerosis. JACC Cardiovasc Imaging.

[107] Vinter Nicklas (2017). Relation of Coronary Artery Calcium Score and Risk of Cancer (from a Danish Population-Based Follow-up Study in Patients Who Underwent Cardiac Computed Tomography). The American Journal of Cardiology.

[108] Whelton Seamus P. (2018). Coronary artery calcium and the competing long-term risk of cardiovascular vs. cancer mortality: the CAC Consortium. Eur. Heart J..

[109] Tota-Maharaj Rajesh (2014). The Relationship of Coronary Artery Calcium To Coronary Heart Disease Events is Similar in Young and Elderly Participants in The Multi-Ethnic Study of Atherosclerosis: A secondary Analysis of a Prospective Population-based Cohort. Mayo Clin Proc..

[110] Miedema Michael D. (2019). Association of Coronary Artery Calcium With Long-term, Cause-Specific Mortality Among Young Adults. JAMA Network Open.

[111] Spring Bonnie (2014). Healthy Lifestyle Change and Subclinical Atherosclerosis in Young Adults: Coronary Artery Risk Development in Young Adults (CARDIA) Study. Circulation.

[112] Hartiala Olli (2012). Adolescence Risk Factors Are Predictive of Coronary Artery Calcification at Middle Age: The Cardiovascular Risk in Young Finns Study. J. Am. Coll. Cardiol..

[113] Kuller Lewis H. (2016). Subclinical Cardiovascular Disease and Death, Dementia, and Coronary Heart Disease in Individuals Age 80+. Journal of American College of Cardiology.

[114] Kawasaki Ryo (2011). Is Diabetic Retinopathy Related to Subclinical Cardiovascular Disease?. Ophthalmology.

[115] Choi Yuni (2015). Egg consumption and coronary artery calcification in asymptomatic men and women. Atherosclerosis.

[116] Qin Chenxi (2018). Associations of egg consumption with cardiovascular disease in a cohort study of 0.5 million Chinese adults. Heart.

[117] Hisamatsu Takashi (2016). Smoking, Smoking Cessation, and Measures of Subclinical Atherosclerosis in Multiple Vascular Beds in Japanese Men. Journal of the American Heart Association.

[118] Greenland Philip (2018). Coronary Calcium Score and Cardiovascular Risk. J. Am. Coll. Cardiol..

[119] Durhan Gamze (2014). Does coronary calcium scoring with a SCORE better predict significant coronary artery stenosis than without? Correlation with computed tomography coronary angiography. Eur Radiol.

[120] Dayan Akın (2012). Coronary calcium score, albuminuria and inflammatory markers in type 2 diabetic patients: Associations and prognostic implications. Diabetes Res. Clin. Pract..

[121] Malik (2020). Exercise Capacity, Coronary Artery Fatty Plaque, Coronary Calcium Score, and Cardiovascular Events in Subjects With Stable Coronary Artery Disease. Journal of the American Heart Association.

[122] Kleiven (2020). Endurance exercise training volume is not associated with progression of coronary artery calcification. Scandinavian Journal of Medicine & Science in Sport.

[123] Kermott (2019). Cardiorespiratory Fitness and Coronary Artery Calcification in a Primary Prevention Population, Mayo Clinic Proceedings: Innovations. Quality & Outcomes.

[124] Aengevaeren (2017). Relationship Between Lifelong Exercise Volume and Coronary Atherosclerosis in Athletes. Circulation.

[125] Hamer (2010). Walking speed and subclinical atherosclerosis in healthy older adults: the Whitehall II study. Heart.

[126] Jae (2016). Relation of Cardiorespiratory Fitness to Risk of Subclinical Atherosclerosis in Men With Cardiometabolic Syndrome. The American Journal of Cardiology.

[127] LF-DeFina et al., Association of All-Cause and Cardiovascular Mortality With High Levels of Physical Activity and Concurrent Coronary Artery Calcification, JAMA Cardiology, 2019, 10.1001/jamacardio.2018.4628. 10.1001/jamacardio.2018.4628 PMC6439619 30698608

[128] Rozanski et al., Associations Among Self-reported Physical Activity, Coronary Artery Calcium Scores, and Mortality Risk in Older Adults, 2020, 10.1016/j.mayocpiqo.2020.02.005. 10.1016/j.mayocpiqo.2020.02.005 PMC7283959 32542214

[129] Kermott (2013). Self-rated stress is noncontributory to coronary artery disease in higher socioeconomic strata. Population Health Management.

[130] Radford (2018). Cardiorespiratory Fitness, Coronary Artery Calcium, and Cardiovascular Disease Events in a Cohort of Generally Healthy Middle-Age Men: Results From the Cooper Center Longitudinal Study. Circulation.

[131] Gao J.W. (2021). Low-Carbohydrate Diet Score and Coronary Artery Calcium Progression: Results From the CARDIA Study. Arterioscler. Thromb. Vasc. Biol..

[132] Rozanski (2021). Relation of Intake of Saturated Fat to Atherosclerotic Risk Factors, Health Behaviors, Coronary Atherosclerosis, and All-Cause Mortality Among Patients Who Underwent Coronary Artery Calcium Scanning. The American Journal of Cardiology.

[133] Frolich S. (2017). Association of dietary patterns with five-year degree and progression of coronary artery calcification in the Heinz Nixdorf Recall study, Nutrition. Metabolism and Cardiovascular Diseases.

[134] Anderson (2016). Calcium Intake From Diet and Supplements and the Risk of Coronary Artery Calcification and its Progression Among Older Adults: 10-Year Follow-up of the Multi-Ethnic Study of Atherosclerosis (MESA). Journal of the American Heart Association.

[135] Ghosh (2021). Whole milk consumption is associated with lower risk of coronary artery calcification progression: evidences from the Multi-Ethnic Study of Atherosclerosis. Eur. J. Nutr..

[136] Won Ki-Bum (2020). Triglyceride glucose index is an independent predictor for the progression of coronary artery calcification in the absence of heavy coronary artery calcification at baseline. Cardiovascular Diabetology.

[137] Park (2019). Elevated TyG Index Predicts Progression of Coronary Artery Calcification. Diabetes Care.

[138] Generoso (2019). High-density Lipoprotein-cholesterol Subfractions and Coronary Artery Calcium: The ELSA-Brasil Study. Arch. Med. Res..

[139] Bittencourt (2017). Relation of Fasting Triglyceride-Rich Lipoprotein Cholesterol to Coronary Artery Calcium Score (from the ELSA-Brasil Study). The American Journal of Cardiology.

[140] Eun Y.M., Kang S.G., Song S.W. (2016). Fasting plasma glucose levels and coronary artery calcification in subjects with impaired fasting glucose. Annals of Saudi Medicine.

[141] Leigh (2019). Coronary Artery Calcium Scores and Atherosclerotic Cardiovascular Disease Risk Stratification in Smokers. JACC Cardiovascular Imaging.

[142] Rifai Al (2018). The prevalence and correlates of subclinical atherosclerosis among adults with low-density lipoprotein cholesterol <70 mg/dL: The Multi-Ethnic Study of Atherosclerosis (MESA) and Brazilian Longitudinal Study of Adult Health (ELSA-Brasil). Atherosclerosis.

[143] Carroll (2017). Interaction between smoking and depressive symptoms with subclinical heart disease in the Coronary Artery Risk Development in Young Adults (CARDIA) study. Health Psychol..

[144] Carroll (2017). Association of the Interaction Between Smoking and Depressive Symptom Clusters With Coronary Artery Calcification: The CARDIA Study. Journal of Dual Diagnosis.

[145] Chevli (2020). Association of Alcohol Consumption and Ideal Cardiovascular Health Among South Asians: The Mediators of Atherosclerosis in South Asians Living in America (MASALA) Study. Alcohol. Clin. Exp. Res..

[146] Mahajan (2018). Data on alcohol consumption and coronary artery calcification among asymptomatic middle-aged men for the ERA-JUMP study. Data in Brief.

[147] Chevli (2020). Alcohol consumption and subclinical atherosclerosis among South Asians: Findings from the Mediators of Atherosclerosis in South Asians Living in America (MASALA) study, Nutrition. Metabolism and Cardiovascular Diseases.

[148] Lee (2017). Effect of Coronary Artery Calcification Score by Lifestyle and Correlation With Coronary Artery Stenosis by Multidetector Computed Tomography. J. Comput Assist Tomogr.

[149] Baek (2021). Concurrent smoking and alcohol consumers had higher triglyceride glucose indices than either only smokers or alcohol consumers: a cross-sectional study in Korea. Lipids Health Dis..

[150] Kimani (2019). Differences Between Coronary Artery Calcification and Aortic Artery Calcification in Relation to Cardiovascular Disease Risk Factors in Japanese Men. Journal of Atherosclerosis and Thrombosis.

[151] Pedrosa at al., Relation of Thoracic Aortic and Coronary Artery Calcium to Cardiovascular Risk Factors (from The Brazilian Longitudinal Study of Adult Health [ELSA-Brazil]), The American Journal of Cardiology, 2019, 10.1016/j.amjcard.2019.08.029. 10.1016/j.amjcard.2019.08.029 31590910

[152] Yun (2017). Alcohol and coronary artery calcification: an investigation using alcohol flushing as an instrumental variable. Int. J. Epidemiol..

[153] Nam J.S. (2020). Association between atherogenic index of plasma and coronary artery calcification progression in Korean adults. Lipids Health Dis..

[154] Razavi A.C. (2021). Predicting Long-Term Absence of Coronary Artery Calcium in Metabolic Syndrome and Diabetes: The MESA Study. JACC Cardiovascular Imaging.

[155] Scicali R. (2021). High TG to HDL ratio plays a significant role on atherosclerosis extension in prediabetes and newly diagnosed type 2 diabetes subjects. Diabetes/Metabolism Research and Reviews.

[156] DeBarmore B. (2018). Association of ambulatory blood pressure variability with coronary artery calcium. J. Clin Hypertens..

[157] Kutkiene S. (2019). Is the coronary artery calcium score the first-line tool for investigating patients with severe hypercholesterolemia. Lipids Health Dis..

[158] De Block C.E.M. (2018). Coronary artery calcifications and diastolic dysfunction versus visceral fat area in type 1 diabetes: VISCERA study. Journal of Diabetes and It‘s Complications.

[159] Kowall B. (2017). Progression of coronary artery calcification is stronger in poorly than in well controlled diabetes: Results from the Heinz Nixdorf Recall Study. Journal of Diabetes and It‘s Complications.

[160] Saremi A., Bahn G.D., Reaven P.D. (2016). A Link Between Hypoglycemia and Progression of Atherosclerosis in the Veterans Affairs Diabetes Trial (VADT). Diabetes Care.

[161] Cho Y.K. (2020). Triglyceride Glucose-Waist Circumference Better Predicts Coronary Calcium Progression Compared with Other Indices of Insulin Resistance: A Longitudinal Observational Study. Journal of Clinical Medicine.

[162] Kim H.J., Kim J.H., Joo M.C. (2018). Association of Exercise Capacity, Cardiac Function, and Coronary Artery Calcification with Components for Metabolic Syndrome. Biomed Res. Int..

[163] Tzoulaki I. (2019). Serum metabolic signatures of coronary and carotid atherosclerosis and subsequent cardiovascular disease. Eur. Heart J..

[164] Diederichsen S.Z. (2017). CT-Detected Growth of Coronary Artery Calcification in Asymptomatic Middle-Aged Subjects and Association With 15 Biomarkers. JACC. Cardiovascular Imaging.

[165] Saremi A. (2017). Advanced Glycation End Products, Oxidation Products, and the Extent of Atherosclerosis During the VA Diabetes Trial and Follow-up Study. Diabetes Care.

[166] Chung Y.H. (2021). Coronary calcification is associated with elevated serum lipoprotein (a) levels in asymptomatic men over the age of 45 years: A cross-sectional study of the Korean national health checkup data. Medicine (Baltimore).

[167] Garg P.K. (2015). Lipoprotein-associated phospholipase A2 and risk of incident cardiovascular disease in a multi-ethnic cohort: The multi ethnic study of atherosclerosis. Atherosclerosis.

[168] V. S. Nunes, The coronary artery calcium score is linked to plasma cholesterol synthesis and absorption markers: Brazilian Longitudinal Study of Adult Health, Bioscience Reports, 2020, 0.1042/BSR20201094. 10.1042/BSR20201094 PMC7332684 32579186

[169] Ceponiene I. (2021). Association of Coronary Calcium, Carotid Wall Thickness, and Carotid Plaque Progression with Low-Density Lipoprotein and High-Density Lipoprotein Particle Concentration Measured by Ion Mobility (From Multiethnic Study of Atherosclerosis [MESA]). The American Journal of Cardiology.

[170] Cao J. (2020). Apolipoprotein B discordance with low-density lipoprotein cholesterol and non-high-density lipoprotein cholesterol in relation to coronary artery calcification in the Multi-Ethnic Study of Atherosclerosis (MESA). Journal of clinical lipidology.

[171] Djekic D. (2019). Serum untargeted lipidomic profiling reveals dysfunction of phospholipid metabolism in subclinical coronary artery disease. Vascular Health and Risk Management.

[172] Cahalane R.M. (2021). On the association between circulating biomarkers and atherosclerotic calcification in a cohort of arterial disease participants, Nutrition. Metabolism & Cardiovascular Diseases.

[173] de Miranda E.J.F.P. (2017). Thyrotrophin levels and coronary artery calcification: Cross-sectional results of the Brazilian Longitudinal Study of Adult Health (ELSA-Brasil). Clin. Endocrinol..

[174] Lee S. (2016). A significant positive association of vitamin D deficiency with coronary artery calcification among middle-aged men: for the ERA JUMP study. J. Am. Coll. Nutr..

[175] Sanchez R.P. (2016). Serum magnesium is inversely associated with coronary artery calcification in the Genetics of Atherosclerotic Disease (GEA) study. Nutr. J..

[176] Gronhoj M.H. (2016). Associations between calcium-phosphate metabolism and coronary artery calcification; a cross sectional study of a middle-aged general population. Atherosclerosis.

[177] Harada P.H. (2019). Composite acute phase glycoproteins with coronary artery calcification depends on metabolic syndrome presence – The Brazilian Longitudinal Study of Adult Health (ELSA-Brasil). J. Cardiol..

[178] Ong K.L. (2019). Relationship of fibroblast growth factor 21 with subclinical atherosclerosis and cardiovascular events: Multi-Ethnic Study of Atherosclerosis. Atherosclerosis.

[179] Goldwater D. (2019). Interleukin-10 as a predictor of major adverse cardiovascular events in a racially and ethnically diverse population: Multi-Ethnic Study of Atherosclerosis. Ann. Epidemiol..

[180] Diederichsen M.Z. (2018). Prognostic value of suPAR and hs-CRP on cardiovascular disease. Atherosclerosis.

[181] Mehta A. (2018). Inflammation and coronary artery calcification in South Asians: The Mediators of Atherosclerosis in South Asians Living in America (MASALA) study. Atherosclerosis.

[182] Larsen B.A. (2017). Adipokines and severity and progression of coronary artery calcium: Findings from the Rancho Bernardo Study. Atherosclerosis.

[183] Tibuakuu M. (2019). GlycA, a novel inflammatory marker, is associated with subclinical coronary disease. AIDS.

[184] Bell E.J. (2018). Hepatocyte growth factor is associated with progression of atherosclerosis: The Multi-Ethnic Study of Atherosclerosis (MESA). Atherosclerosis.

[185] Jun J.E. (2018). Elevated serum uric acid predicts the development of moderate coronary artery calcification independent of conventional cardiovascular risk factors. Atherosclerosis.

[186] Beloqui O. (2017). Increased phagocytic NADPH oxidase activity associates with coronary artery calcification in asymptomatic men. Free Radical Res..

[187] N. Ei Ei Khaing et al., Epicardial and visceral adipose tissue in relation to subclinical atherosclerosis in a Chinese population, PLOS ONE, 2018, 10.1371/journal.pone.0196328. 10.1371/journal.pone.0196328 PMC5919010 29694442

[188] Cosson E. (2021). Epicardial adipose tissue volume and coronary calcification among people living with diabetes: a cross-sectional study. Cardiovascular Diabetology.

[189] Nam H.Y., Jun S. (2017). Association between active brown adipose tissue and coronary artery calcification in healthy men. Schattauer.

[190] Chung G.E. (2021). Sarcopenic Obesity Is Significantly Associated With Coronary Artery Calcification. Frontiers in Medicine.

[191] Ko Byung-Joon (2016). Relationship Between Low Relative Muscle Mass and Coronary Artery Calcification in Healthy Adults. Arterioscler. Thromb. Vasc. Biol..

[192] Murakami Y. (2016). Renal sinus fat volume on computed tomography in middle-aged patients at risk for cardiovascular disease and its association with coronary artery calcification. Atherosclerosis.

[193] Serrano C.V. (2018). Association between Neutrophil-Lymphocyte and Platelet-Lymphocyte Ratios and Coronary Artery Calcification Score among Asymptomatic Patients: Data from a Cross-Sectional Study. Mediators Inflamm..

[194] de Almeida-Pititto B. (2016). Usefulness of circulating E-selectin to early detection of the atherosclerotic process in the Brazilian Longitudinal Study of Adult Health (ELSA-Brasil). Diabetology & Metabolic Syndrome.

[195] Bielinski S.J. (2015). P-selectin and subclinical and clinical atherosclerosis: the Multi-Ethnic Study of Atherosclerosis (MESA). Atherosclerosis.

[196] Howlett P. (2015). MicroRNA 8059 as a marker for the presence and extent of coronary artery calcification. Open Heart.

[197] Mahabadi (2016). CAC Score Improves Coronary and CV Risk Assessment by ESC and AHA/ACC Primary Prevention Guidelines. JACC: Cardiovascular Imaging.

[198] Mitchell (2018). Impact of Statins on Cardiovascular Outcomes Following Coronary Artery Calcium Scoring. J. Am. Coll. Cardiol..

[199] Al Rifai M. (2020). Coronary Artery Calcification, Statin Use and Long-Term Risk of Atherosclerotic Cardiovascular Disease Events (from the Multi-Ethnic Study of Atherosclerosis). The American Journal of Cardiology.

[200] Mortensen M.B. (2018). Statin Trials, Cardiovascular Events, and Coronary Artery Calcification Implications for a Trial-Based Approach to Statin Therapy in MESA. JACC: Cardiovascular Imaging.

[201] Pursnani A. (2015). Guideline-Based Statin Eligibility, Coronary Artery Calcification, and Cardiovascular Events. JAMA.

[202] Shah R.V. (2017). Subclinical Atherosclerosis, Statin Eligibility, and Outcomes in African American Individuals, The Jackson Heart Study. JAMA Cardiology.

[203] Liberati (2009). The PRISMA statement for reporting systematic reviews and meta-analyses of studies that evaluate healthcare interventions: explanation and elaboration. BMJ.

[204] Higgins et al., Cochrane Handbook for Systematic Reviews of Interventions, Second Edition, John Wiley & Sons, 2019, 10.1002/9781119536604.

[205] (2020). Review Manager (RevMan) [Computer Program]. Version 5.4. The Cochrane Collaboration.

[206] Rockenfeller (2015). Phosphatidylethanolamine positively regulates autophagy and longevity. Cell Death Differ..

[207] Maceyka M., Spiegel S. (2014). Sphingolipid metabolites in inflammatory disease. Nature.

[208] Roberts E.T. (2015). Cost-effectiveness of Coronary Artery Calcium Testing for Coronary Heart and Cardiovascular Disease Risk Prediction to Guide Statin Allocation: The Multi-Ethnic Study of Atherosclerosis (MESA). PLoS ONE.

[209] Hong J.C. (2017, 10.106/j.jcmg.2017.04.014.). Implications of Coronary Artery Calcium Testing for Treatment Decisions Among Statin Candidates According to the ACC/AHA Cholesterol Management Guidelines: A Cost-Effectiveness Analysis. JACC: Cardiovascular Imaging.

[210] Spahillari A. (2020). Cost-effectiveness of Contemporary Statin Use Guidelines with or without Coronary Artery Calcium Assessment in African American Individuals. JAMA Cardiology.

[211] Venkataraman P. (2020). Cost-Effectiveness of Coronary Artery Calcium Scoring in People with a Family History of Coronary Disease. JACC: Cardiovascular Imaging.

[212] M. Cainzos-Achirica et al., Coronary Artery Calcium for Personalized Allocation of Aspirin in Primary Prevention of Cardiovascular Disease in 2019: The MESA Study, Circulation, 2020, 10.1161/CIRCULATIONAHA.119.045010. 10.1161/CIRCULATIONAHA.119.045010 PMC7217722 32233663

[213] Ajufo E. (2020). Value of Coronary Artery Calcium Scanning in Association with the Net Benefit of Aspirin in Primary Prevention of Atherosclerotic Cardiovacular Disease. JAMA Cardiology.

[214] McEvoy J.W. (2017). Coronary Artery Calcium to Guide a Personalized Risk-based Approach to Initiation and Intensification of Antihypertensive Therapy. Circulation.

[215] N. k. Kalia et al., Motivational effects of coronary artery calcium scores on statin adherence and weight loss, Coronary Artery Disease, 2015, 101097/MCA.0000000000000207. 10.1097/MCA.0000000000000207 25514570

[216] Venkataraman P. (2021, 10.106/j.atherosclerosis.2021.08.002.). Impact of a coronary artery calcium guided statin treatment protocol on cardiovascular risk at 12 months: Results from a pragmatic, randomized controlled trial. Atherosclerosis.

[217] Khera A. (2018). Astronaut Cardiovascular Health and Risk Modification (Astro-CHARM) Coronary Calcium Atherosclerotic Cardiovascular Disease Risk Calculator. Circulation.

[218] Mulders T.A. (2016). Coronary artery calcification score as tool for risk assessment among families with premature coronary artery disease. Atherosclerosis.

[219] van Iperen E.P.A. (2016). Genetic analysis of emerging risk factors in coronary artery disease. Atherosclerosis.

[220] Choi Se-Yeon (2019). Genome-wide association study of coronary artery calcification in asymptomatic Korean populations. PLoS ONE.

[221] Wing J.J. (2016). Change in Neighborhood Characteristics and Change in Coronary Artery Calcium: A Longitudinal Investigation in the MESA Cohort. Circulation.

[222] Abdulla A.G. (2019). Association of psychosocial traits with coronary artery calcium development and progression: The Multi-Ethnic Study of Atherosclerosis. J. Cardiovasc. Comput. Tomogr..

[223] Miname M.H. (2018). Coronary Artery Calcium and Cardiovascular Events in Patients with Familial Hypercholesterolemia Receiving Standard Lipid Lowering Therapy. JACC: CARDIOVASCULAR IMAGING.

[224] Feuchtner G. (2021). The effect of omega-3 fatty acids on coronary atherosclerosis quantified by coronary computed tomography angiography. Clinical Nutrition.

[225] Zeb I. (2017). Randomized trial evaluating the effect of aged garlic extract with supplements versus placebo on adipose tissue surrogates for coronary atherosclerosis progression. Coron. Artery Dis..

[226] Pandey A.K. (2014). Family history of coronary heart disease and the incidence and progression of coronary artery calcification: Multi-Ethnic Study of Atherosclerosis (MESA). Atherosclerosis.

[227] Patel A.A. (2018). Radiation exposure and coronary artery calcium scans in the society for heart attack prevention and eradication cohort. Int. J. Cardiovasc. Imaging.

