# Addendum and Corrigendum to “Subclinical cardiovascular disease and utility of coronary artery calcium score” [IJC Heart Vasculat. 37 (2021) 100909]

**DOI:** 10.1016/j.ijcha.2023.101208

**Published:** 2023-04-21

**Authors:** Cihan Durmuş Saydam

**Affiliations:** Muhsin Yazıcıoğlu Cad., No:10 Üst Kaynarca Pendik, Istanbul 34899, Turkey

The author regrets for any changes required to be corrected by the corrigendum, and to be indicated by the addendum. The author would like to apologize for any inconvenience caused.  

**Addendum and Corrigendum**

**Addendum Section 7:**

• On the page-3 at the third paragraph, NRI (Net Reclassification Improvement) was defined in practical view of CACs use as in reclassification of the intermediate risk (according to conventional risk models) into high risk and low risk, however a broader definition of NRI was thought contributory as expanding discussions including reclassifications of high and low risk categories or when more than 3 risk categories exist, and then in accordance the paragraph was edited as follows:
“Overall Net Reclassification Improvement (NRI) is defined as the summation of two subtraction operations belonging to net reclassifications into expected event (related to sensitivity) and non-event (related to specificity) in theoretical range of −2 to +2, where the subtractions between the probability-percentage of being correctly reclassified event-risk or category and the probability-percentage of being incorrectly reclassified event-risk or category for higher risk or up-classification (up-stratification) and lower risk or down-classification (down-stratification) are summed, and as in the case of re-stratification by CACs at intermediate risk by conventional risk models to obtain overall-NRI the net reclassifications for high risk and low risk are summed [50].”  
• On the page-3 at the paragraph-4 reclassifications into event or non-event after combining CACs to FRS were noticed requiring re-definitions to clarify whether recategorization percentages in 2 components were net changes or correct/incorrect reclassifications. In reference study overall-NRI and NRI were interchangeably used. The relavant paragraph section was edited with an additional explanation for why novel markers of carotid IMT and brachial FMD were excluded from revealing NRI as follows:
“(…) showed that by combining CACs to FRS, 25.5% of events group had been net re-classified to high risk and 40.4% of the non-event group had been net re-classified to low risk, and therefore NRI had been calculated as 0.659 or 65.9%. In this follow-up study 5 more novel risk markers, which are Carotid IMT, ABI, Brachial FMD, hs-CRP and Family History, were added to baseline model of FRS but in multivariable adjusted model (of age, gender, ethnicity, SBP, TC, HDL-c, Smoking Status, BMI, Antihypertensive and Statin use) for both incident CHD and CVD events c-IMT and brachial-FMD had no significant associations. (…)”  
• On the page-3 at the paragraph-5 the percentage of the association between risk factors and incident CVD mediated by Ln[CACs + 25] was reported only for age and male gender because the shared risk remained below 50% for other risk factors, but further delineating the proportions of the CVD risk due to other risk factors in mediation by Ln[CACs + 25] may be contributory to further discussions. The relavant clause was edited with extending the interactions of Ln[CACs + 25] on the associations between conventional risk factors and incident CVD-event as follows:
“Ln(CAC + 25) could have share of risk (for incident CVD) associate with age and male sex as 80.2% and 52.2%, whereas weaker mediation to the incident CVD for other conventional risk factors of BMI (40.9%), TC (22.0%), DM (19.4%), current smoker (10.6%), SBP (10.0%) and FRS (42.5), moreover, Ln(CAC + 25) had significant interactions for the associations between incident CVD and conventional risk factors of age (HR:0.86), SBP (HR:0.95) and FRS (HR:0.85).”  
• On the page-4 at the 2nd paragraph the associations of CAC volume and density scores with CVD risk factors by multivariable regression models (fully-adjusted) were presented without showing β values. However, revealing strength of associations re-deemed contributory for further discussions across the study. Thus the relavant clauses were edited as follows:
“(…) by fully-adjusted multivariable regression models ln CAC volume score was discerned in positive association with (per 1-SD change in continuous variables) CAC density score (each 0.695 unit, β:0.94), age (per 9.53 years, β:0.31), male gender (β:0.53), college or greater level of education (β:0.17), total cholesterol (β:0.06), diabetes (β:0.44), antihypertensive medication use (β:0.21), BMI (β:0.09), family history of MI (β:0.20) and the amount of alcohol consumption (each 9.1 drinks/week, β:0.05), while inversely associate with high annual income (β:−0.22), CRP (each 6.29 mg/dl, β:−0.07), and walking at stride (β:−0.45), brisk (β:−0.29) and average (β:−0.27) paces. CAC density was shown in positive association with Ln CAC volume score (each 1.62 units, β:0.44), high-income (β:0.12), HDL-c (β:0.03) and brisk walking pace (β:0.12), while male gender (β:−0.07), diabetes (β:−0.07), and BMI (−0.04) were in inverse association.”  

**Corrigendum Section 7:**

• On the page-4 at the 3rd paragraph, it was realized that the event-rate of hard CHD among participants with a FH of premature CHD and CACs ≥ 400 (per 1000 person-years) was unwittingly noted slightly lower than it’s reported value (12.1 vs 14.5). Author apologizes for any inconvenience and the relavant paragraph section was edited with added explanations on analyses as follows:
“A MESA study [13], enrolled 6814 adults without ASCVD along median follow-up time of 10.2 years, in multivariable adjusted models (of conventional risk factors) revealed prevalance of hard ASCVD and CHD events are higher among those with a FH of premature CHD compared to those without at an adjusted HR of 1.35 (1.07–1.71) and 1.41 (1.05–1.88), respectively. Highest hard CHD events was observed in those with a FH of premature CHD and CAC ≥ 400 at 14.5 per 1000 person years. (…)”  

**Erratum Section-7:**

• On the page-3 at the paragraph-11 doi number for the cited reference study (https://doi.org/10.1016/j.jcmg.2017.04.016) was realized slightly different than it should be (only at the last digit). So, the doi number for the reference study 47 was edited into “https://doi.org/10.1016/j.jcmg.2017.04.018”; nevertheless, unwittingly cited article was also added to reference for citing the previous paragraph-10 as with reference study 46 as follows: "(...) however CAC score  ≥ 100 indicates at least a 7.5% 10 year risk of ASCVD [46, 242[15]].". Newly added reference study-242 is as follows: “P. H. Joshi, et al., The 10-Year Prognostic Value of Zero and Minimal CAC, JACC: Cardiovascular Imaging (2017), https://doi.org/10.1016/j.jcmg.2017.04.016”.  

**Addendum Section 9:**

• On the page-4 at the paragraph-7 for gender specific patterns of age related calcifications distinct age or age intervals were not revealed. However, it was re-deemed possibly contributory for further discussions along the study, and the relavant paragraph section was edited as follows:
“A cross-sectional study by Y. Ohmoto-Sekine et al. [105] as analysis of 1834 Japanese participants volunteered for simultaneous CAC scoring and lung cancer screening at one CT-imaging, demonstrated males in contrast with females CAC score increase by aging begins 10 years earlier (40 vs 50) with more gradual annual rate (during 60 to 70 years of age) nonetheless cumulatively male gender significantly associated with higher CAC score per se and sharper increase in CACs after 70 years of age, moreover, (…)  

**Erratum Section 9.1:**

• On the page-5 at the paragraph-3 the link for the reference study 127 through it’s doi number appeared not working due to an extra dot at the end of string. The doi number for cited reference study 127 as “https://doi.org/10.1001/jamacardio.2018.4628.” was edited into “https://doi.org/10.1001/jamacardio.2018.4628”.
• On the page-5 at the paragraph- 4 and 5 the link for cited referece study 128 through it’s doi number appeared not working due to an extra dot at the end of string. The doi number for cited reference study 127 as “https://doi.org/10.1016/j.mayocpiqo.2020.02.005.” was edited into “https://doi.org/10.1016/j.mayocpiqo.2020.02.005”.  

**Corrigendum Section 9.1:**

• On the page-5 at the paragraph-3 for the cited reference study-127 an unwitting typing error noting “weakly physical activity duration” was edited into “weekly physical activity duration”, and additionally the binomial association of High vs Low PA for CACs > 0 prevalence was realized requiring a differentiation from linear trends across PA categories. The relevant clause was edited to as follows:
“(…) within mean follow-up period of 10.4 years (models adjusted for age, BMI, blood glucose and cholesterol levels, SBP and smoking status) yet similar for CACs ≥ 100; furthermore, in response to increased PA-level only high vs low PA significantly associated with increased risk of having CAC ≥ 100 (RR-adjusted = 1.11), and across PA levels (low, moderate and high) significant linear trend for weekly physical activity duration and absolute MET-min/week, cardiorespiratory fitness, VO_2_max, proportion of VO_2_max ≥ 50 mL/kg/min and HR-recovery at 1 min also inverse linear trend for maximum-HR were discerned; (…)”  

**Addendum Section 9.1:**

• On the page-5 at the paragraph-1 for the cited reference study-125 model definitions for adjusted covariates and the binomial comparison weren’t unfold. The relevant paragraph section was edited to delineate these analyses as follows:
“Hamer [125] observed participants with CAC = 0 compared to CAC ≥ 400 completed 8-ft walking course 0.16 s faster (adjusted for model-1: age and gender), and objectively assessed faster walking speed (Brisk/Fast vs. slowest, walking time thresholds females: 2.10 s and males: 1.94 s) associated with lower risk of having CAC ≥ 100 (OR = 0.62, adjusted for model-1 + model-2: employment grade and smoking + model-3: SBP, LDL-c, HDL-c, CRP, Fasting glucose, BMI, Short form of 36 scores and lipid-lowering medication use).”
• On the page-5 at the paragraph-2 for the cited reference study-126 odds-ratios were shown without revealing adjusted covariates. The relevant paragraph section was edited as follows:
“(…) higher risk of CAC > 0 (OR = 1.41, adjusted for model-1: Age, CRP, smoking, HTN and DM + peak oxygen consumption) in which number of CMS components correlated with presence of CAC > 0; nonetheless higher fitness levels attenuate these associations at which for each 1 MET (Metabolic Equivalents of Task, 3.5 mL/kg/min) increase in fitness associated with 10% lower odds of having CAC > 0 (adjusted for model-1 + cardiometabolic syndrome) and for fit individuals regardless of having CMS prevalence of CAC > 0 remained similar (in any model).”
• On the page-5 at the 4th paragraph for the cited reference study-129 predictability of a high CACs ≥ 100 vs CACs < 100 by combined model of FRS and self-reported exercise intensity level was noted. However, to widen the discussions with the predictability of other CACs thresholds by these variables the relevant paragraph section was edited as follows:
“(…) Kermott et al. [129] discerned dichotomies at thresholds of CAC Score ≥ 200 (vs. CACS < 200), CAC Score ≥ 100 (vs. CACS < 100) and CAC Score ≥ 5 (vs. CACS < 5) but not CACs > 0 (vs. CACs = 0) can be significantly predicted by combined explanatory variables of Framingham score and self-reported exercise intensity level in univariate models. (…)”
• On the page-5 at the paragraph-4 for the cited reference study-130 unshown covariates in adjusted models of all analyses were added in the relevant paragraph section as follows:
“Radford et al. [130] reported total CVD-incidence rates in CAC ≥ 400 vs CAC = 0 stratify with CRF (Cardiorespiratory Fitness) of 5 METs and 15 METs as 5-fold and 2-fold increase (all-analyses adjusted for Age, Smoking, BMI, SBP, HTN, Glucose, DM, TC and statin therapy).”  

**Addendum Section 9.2.1:**

• On the page-6 at the paragraph-1 adjusted covariates in analyses weren’t revealed. The relevant paragraph section was edited as follows:
“Gao et al. [131] observed an inverse non-linear association of carbohydrate intake with CAC-s progression which is most pronounced for male-vs-female genders and white-vs-black ethnicities in Cox regression analysis (adjusted for multivariable model covariates excepts baseline-CACs), and in multivariable-adjusted models (of age, ethnicity, education level, alcohol, smoking, baseline-CACs, BMI, SBP, DBP, dietary intakes of calcium, fiber, magnesium and Vit D, FPG, TG, HDLc, LDLc, HTN, DM, PA and serum-creatinine) across 3 categories of carbohydrate intake as (…).”.  

**Erratum Section 9.2.1:**

• On the page-5 at the paragraph-6 the link for the reference study 70 appear not functioning through its doi number due a single digit error in the string. Reported doi number as “https://doi.org/10.1017/S0007114518002513” was edited into “https://doi.org/10.1017/S0007114518003513”.  

**Corrigendum Section 9.2.2:**

• On the page-6 at the end of paragraph-4 term labeling p values as for the interactions were noticed lacking, and the relevant clause was edited with additional analyses and model adjustments as below. Author apologizes for any inconvenience.
“(…) significantly associated with lower risk of CVD (HR:0.70), CAD (HR:0.69) and stroke (HR:0.64) mortalities in multivariable adjusted models-2 (of model-1: age, gender, dialect, year of interview, educational level + Model-2: BMI, PA, smoking, alcohol, HTN, total energy intake, dietary supplement use) and in additional adjustment with either only Na intake or intakes of Ca, Mg, Na and K these associations remained significant, nevertheless, in multivariable-adjusted models-2 across quintiles of intake with CVD mortality Ca, Mg and K inversely and Na positively associated yet in additional adjustment (Model-2 + PUFA/SFA-ratio, dietary intakes of cholesterol, Long-chain Fatty Omega-3 FA, other Omega-3 FA and fibers) only intakes of Na and K remained significant and dietary intake of fiber mostly involved in this attenuation, moreover, in multivariable adjusted models-2 6 out of 8 components of DASH score (Vegetables, Fruits, whole grains, nuts and legumes, low red meat and low sodium) across quintiles inversely associated with CVD mortality, furthermore, in sensitivity analysis in interaction of current smoking with the adherence to the DASH-diet, which significantly correlated with higher intakes of calcium, potassium, magnesium, fiber, folate, vitamin D and polyunsaturated versus saturated fatty acids, and lower intakes of cholesterol and sodium, those significant associations weakened but remained significant as p-interaction = 0.08 for CVD and stroke mortalities, and nonsignificant interaction as p-interaction = 0.7 for CAD mortality.”  

**Addendum Section 9.2.2:**

• On the page-6 at the paragraph-5 for cited reference study-133 adjusted covariates for relative-risks weren’t defined. The relevant paragraph section was edited as follows:
“(…) in multivariable-adjusted models (of model-3: unadjusted + model-2: age and education + model-3: HTN, DM, LDL-c, HDL-c and BMI) was most significantly observed for those with Mediterranean-like dietary pattern for both genders (Male RR = 0.61 and Female RR = 0.59 in model-2, Male RR = 0.62 and Female RR = 0.59 in model-3), moreover, among females Health-conscious (RR = 0.63 in model-2, RR = 0.62 in model-3) and Traditional German/less alcohol (RR = 0.69 in model-2, RR = 0.67 in model-3) dietary patterns also associated with reduced risk for rapid progression of CAC percentile score yet Western dietary pattern remained similar (in any model).”.  

**Addendum Section 9.2.3:**

• On the page-6 at the paragraph-7 covariates of model adjustments weren’t revealed and the relevant paragraph section was edited with displayed covariates of fully adjusted multivariable models as follows:
“ (…) and compared to non-drinkers attendants with ≥5 sugar-added carbonated beverages per week had significant 1.70 CAC score ratio (of Ln transformed Ln[CACs + 1] by Tobit regression models in model-2, and model-1: 1.86) and 1.27 odds ratio of having non-zero CAC score in multivariable-adjusted models (model-1: age, gender, center, year of examination + model-2: education-level, PA, smoking, alcohol, FH of CVD, HTN, Hypercholesterolemia, and intake of total energy, fruits, vegetables and red/processed meat; in model-2, and model-1: 1.31).”  

**Corrigendum Section 9.2.3:**

• On the page-6 at the paragraph-8 attenuated association of serum EPA with CDS after further adjustment for a second batch of listed covariates was noticed unclarified with an incomplete phrase. Author apologizes for any inconvenience. The relevant clause was edited as follows:
“(…) however, serum EPA and total OM3 significantly inversely associated with CDS in model adjustment for age, used CT-device type, CAC score, hypertension, diabetes, LDL, HDL, smoking pack-year, and BMI, but attenuated after further adjustment along with for CRP, triglycerides, lipid-lowering medication, and histories of CVD and CKD.”
• On the page-7 at the paragraph-1 in associations with egg consumption 2 CACs ratios were noticed unwittingly reported among binomial results with odds ratios instead of score ratios as in rate ratios. Author apologizes for any inconvenience, and this paragraph was edited with additional analyses and clarifications on analyses by further model adjustments as follows:
“Choi et al. [115], including 23,417 asymptomatic Korean adults from the Kangbuk Samsung Health Study into cross-sectional analysis, demonstrated in multivariable-adjusted models (of age, gender, center, year of examination, education level, PA-level, smoking, BMI, parental history of CVD, HTN, alcohol, intakes of total energy, fruits, vegetables and red/processed meats) eggs consumption ≥ 7 eggs/week compared to reference egg consumption < 1 egg/week significantly associates with greater any nonzero CACs ratios (by Tobit regression of Ln[CACs + 1], RR:1.80, CI:1.14–2.83), and higher prevalence of any CAC 1–100 (OR:1.21, CI:0.98–1.50, p-trend:0.03) and CAC > 100 (OR:1.75, CI:1.14–2.68, p-trend:0.02), and each increase of 1 egg/day associates with greater CACs ratios (RR:1.54, CI:1.11–2.14), and higher prevalence of CAC 1–100 (OR:1.16, CI:1.00–1.36) and CAC > 100 (OR:1.36, CI:0.99–1.89), moreover, further adjustment for TC, HDL-c, TG, glucose, HOMA-IR and DM didn’t modify the positive associations between egg intake and CACs ratios yet additional adjustment for total dietary cholesterol in further adjusted model attenuated these positive associations, nonetheless, in subgroup analysis positive association of egg intake with CACs ratios becomes stronger among participants with higher BMI and lower vegetable intake, however, in sensitivity analysis including participants consumes only egg-whites increasing intake at each 1 egg/day had nonsignificant CACs ratios (RR: 0.71, CI: 0.21–2.34).”
• On the page-7 at the paragraph-2 a slight error in Odds Ratios of ischemic heart disease (IHD) and major coronary events (MCE) in associations with egg consumption of 1 egg/day vs non-consuming were noticed. The relevant clause was edited with an added reveal for covariates of multivariable models as follows:
“(…) as 1 egg each day compared to almost non-consuming per week in multivariable adjusted model (of age, gender, education level, household income, marital status, alcohol, tobacco, physical activity, BMI, WHR, HTN, aspirin, FH of CVD, multivitamin supplementation, dietary pattern) associates with lower risk of CVD (OR:0.89), ischaemic heart disease (IHD; OR:0.88), major caronary event (MCE; OR:0.86) and ischaemic stroke (OR:0.90); (…)”  

**Addendum Section 9.2.4:**

• On the page-7 at the paragraph-5 for the cited reference study-137 model-adjustments weren’t revealed. This paragraph was edited as follows:
“Park [137] with average follow-up period of 4.2 years demonstrated across TyG index tertiles compared to reference T1-tertile Δ√Transformed CAC-score and Annualized-Δ√Transformed CAC-score in univariate models and in multivariable logistic regression (adjusted for age, gender, BMI, SBP, LDL-c, HDL-c, exercise, alcohol, smoking, DM, HTN, use of aspirin and statin, and baseline Ln[CACs + 1]) risk for CAC progression and incidence of CAC > 0 increased.”
• On the page-7 at the paragraph-6 covariates of multivariable adjusted model weren’t shown, and to illustrate the covariates the paragraph was edited as follows:
“Generoso [138] in cross-sectional analysis in multivariable adjusted model-2 (of model-1: age, gender and ethnicity + Model-2: behavioral covariates of smoking, alcohol and physical activity besides LDL-c) observed when model-2 further adjusted with TG-level per 1-SD decrease in Total HDL-c, HDL2-c and HDL3-c and HDL2-c/HDL3-c ratio have no significant association with Ln(CAC + 1), having CAC > 0 vs CAC = 0, having CAC ≥ 100 vs CAC < 100 and ln[CAC], whereas adjustment with LDL-c (in addition to model-1 + behavioral covariates) didn’t attenuated the relations of Total HDL-c, HDL2-c and HDL3-c with either Ln(CAC + 1) or CAC > 0 vs CAC = 0.”  

**Corrigendum/Erratum Section 9.3:**

• On the page-7 at the paragraph-11 was unwittingly subsequently repeated. Author apologizes for any inconveniences. Duplication was removed. Paragraph-11 with added reveal for adjusted covariates as follows:
“Hisamatsu et al. [117], recruiting 1019 the Shiga Epidemiological Study of Atherosclerosis (SESSA) participants derived from general Japanese population aged 40 to 79 years into the study analysis, in multivariable adjusted models (of age, BMI, SBP, TC, HDL-c, CRP, DM, medications for HTN and dyslipidemia, PA and alcohol intake per week) revealed current-smoking excepts for former-smoking significantly associated with higher prevalence of any non-zero CAC > 0 (OR:1.79), CAC ≥ 100 (OR:2.06) and CAC ≥ 400 (OR:2.64), cumulative exposure through pack-years of smoking associated with higher prevalence of non-zero CAC > 0; and attenuation of subclinical atherosclerosis as returning to normal never smoking status takes ≥ 10.4 years at a nonzero 1 < CAC < 100 score, and ≥24.4 years at both 400 > CAC ≥ 100 and CAC ≥ 400 scores.”  

**Addendum Section 9.3:**

• On the page-8 at the paragraph-3 a possible ambiguity was clarified with added pronoun and punctuation to prevent any potential confusions as follows:
“(…) in logistic regression models for each depressive symptom clusters and CES-D both adjusted for sociodemographic (gender, ethnicity and age), clinical (TC, SBP, DBP, BMI and Diabetes) and behavioral covariates (physical activity, alcohol use and depressive symptoms) at 25% cluster, CES-D scores with increasing cumulative smoking exposure > 0 of 10-packyears, 20-packyears and 30-packyears significantly associated with CACs > 0 through increasing odds compared to reference participants with CACs = 0 and 0-cluster score and most evident association was represented for somatic symptom clusters then CES-D score, and both scores reached highest odds at 50% cluster score with 30-packyears as OR:6.68 and OR:5.74, respectively, (…)”
• On the page-8 at the paragraph-4 for the cited reference study-72 covariates of adjusted models, p for trend analysis across the coffee consumption categories and the label of OR for coffee consumption category weren’t shown. This paragraph was edited as follows:
“According to Miranda et al. [72], which is a cross sectional study comprising 4426 individuals, coffee consumption (>3 cups/day vs never/almost never) inversely associate with CACS ≥ 100 at odds ratio of 0.33 (95% CI: 0.17–0.65) after adjustment (for age, gender, ethnicity, education level, BMI, PA-level, smoking, alcohol, saturated-fat intake, total energy intake, consumption of fruit, vegetable and tea, SBP, DBP, fasting glucose, HDL-c, LDL-c, TG, use of antihypertensive, antidiabetic and cholesterol lowering medications); however, deleterious effect of cigarette consumption overwhelms the benefits of coffe intake and only never smokers associate significantly with lower odds of coronary calcification (for >3 cups/day, odds ratio: 0.37, where CI: 0.15–0.91) for habitual coffe intake up to 3 cups/day (across ≤1, 1-3 and >3 cups/day p for trend: 0.036).”.
• On the page-8 at the paragraph-5 for the cited reference study-75 covariates of adjusted-models weren’t revealed. The relevant paragraph section was edited as follows:
“(…) McEvoy et al. [75] in multivariable-models (all models adjusted for age, ethnicity, MESA-site, BMI, HTN, DM, HR, LDL-c, HDL-c, TG, FH of MI, Level of education, Treatment for dyslipidemia) reported that compared to never smokers, current smoker had 80% higher odds of hs-CRP ≥ 2 mg/L and former smokers had 20% higher odds of hs-CRP ≥ 2 mg/L. (…).  

**Addendum Section 9.4:**

• On the page-9 at the paragraph-7 covariates of model adjustments weren’t revealed. This paragraph was edited with adjusted and crude odds ratios as follows:
“Baek [149] recruiting 10,568 Korean KNHANES registered adults and 9586 Korean KOICA registered adults in cross-sectional design with deriving retrospective patient records, demonstrated compared to no use of alcohol and smoking in KHANES participants those with smoking (OR-crude: 2.37, OR-adjusted: 1.56), alcohol use (OR-crude:3.09, OR-adjusted: 2.76) and concurrent use (OR-crude: 4.59, OR-adjusted: 4.33) had greater risk of having high TyG (Triglyceride Glucose) index in multivariable adjusted models (of model-1: age, gender, SBP, TC, HDL-c, regular exercise, smoking and alcohol + education level and WC), consistently also for KOICA registry smoking (OR-crude: 1.80, OR-adjusted: 1.33), alcohol use (OR-crude: 1.70, OR-adjusted: 1.42) and concurrent use (OR-crude: 1.94, OR-adjusted: 1.94) associated with greater risk of high-TyG in multivariable adjusted models (model-1 + BMI), moreover, in KOICA registry no regular exercise also posed greater odds of high-TyG (OR-adjusted: 1.26).”.  

**Corrigendum Section 9.4:**

• On the page-9 at the paragraph-10 strengths of associations in reviewed regression analysis were reported with its β value, however at weaker associations strengths of associations were realized unintentionally not-fully defined, that may cause a confusion with an inverse pattern of association. Reviewed analysis report for A1c(%) and CACs ≥ 1 among females were also extended with including simpler analysis in multivariable adjusted models of same covariates. Author apologizes for any inconvenience. The relevant clause was edited follows:
“(…) furthermore, in Instrumental Variables (IV) analysis of Alcohol flushing log-transformed Log_2_[(Alcohol + 1)/8] for males significantly associated with higher SBP (β = 1.251), DBP (β = 1.373), HDL-c (β = 1.476), TC (β = 2.293), CACs ≥ 1 (OR:1.11), CACs 1–100 (OR:1.10), CAC ≥ 101 (OR:1.21) and CACs in ordinal outcome (OR:1.11) along with other positive associations at lower strength of association as BMI (β = 0.169), WC (β = 0.560), Log-Transformed TG (β = 0.053), Log-Transformed FPG (β = 0.013), Log-Transformed Insulin (β = 0.018) and Log-Transformed HOMA-IR (β = 0.031), and for females significantly associated with higher DBP (β = 1.309) and HDL-c (β = 1.865) besides weaker positive association for Log-Transformed FPG (β = 0.010) and only in simpler analysis as in multivariable models of CV risk factors (adjusted for same covariates of age, education, monthly household income or socioeconomic status, cigarette smoking, physical activity, adult height, medications for DM, HTN and dyslipidemia) but not IV-analysis existing inverse weak association for A1c(%) (β = −0.015) and positive association for binary outcome of CACs ≥ 1 ( vs CACs < 1, OR:1.168); as a summary these analysis suggest facial flushing after alcohol intake could be used for alcohol consumption and health outcome assessments, and alcohol consumption significantly associated with CAC.”.  

**Corrigendum Section 9.5.1:**

• On the page-10 at the paragraph-1, at the last sentence the reference metabolic health category was unwittingly noted as SNO instead of SMH. The relevant paragraph section starting at the line-12 was edited with additional analyses, labeled reference categories at each HRs and adjusted model covariates as follows:
“(…) They observed that, compared to Metabolically Healthy Non-obese (MHNO), Metabolically Unhealthy Non-obese (MUNO) patients had greater probability of incident obesity (HR:1.88, Ref: MHNO), incident diabetes mellitus (HR:4.01, Ref: MHNO), incident hypertension (HR:1.74, Ref: MHNO), incident CVD (HR:1.86, Ref: MHNO), and incident CKD (HR:1.72, Ref: MHNO). MHO (metabolically healthy obese) patients had been observed becoming metabolically unhealthy (HR:1.79; Ref: MHNO), and in predisposition to develop incident DM (HR:3.59; Ref: MHNO), incident Hypertension (HR:2.11; Ref: MHNO) and incident CKD (HR:1.77, Ref: MHNO). As greatest risk category among 4 obesity subphenotypes, MUO (Metabolically Unhealthy Obese) patients had greatest odds for incidence of DM (HR:8.39; Ref: MHNO), hypertension (HR:3.00; Ref: MHNO), CVD (HR:2.40; Ref: MHNO), and CKD (HR:2.43; Ref: MHNO). Among obesity status non-obese patients variable BMI (VNO) compared to reference stable BMI non-obese (SNO) had associated with higher incidence of obesity (HR:2.63, Ref: SNO), metabolically Unhealthy (HR:1.67; Ref: SNO), DM (HR:1.58; Ref: SNO), and hypertension (HR:1.74; Ref: SNO) but not the incident CVD (Ref: SNO) and CKD (Ref: SNO), moreover, obese patients either of stable-BMI with obesity (SO) and variable-BMI with obesity (VO) compared to reference SNO significantly associated with incident metabolically unhealthy (HR:1.93, HR:2.35, respectively; Ref: SNO), DM (HR:2.92, HR:2.16; Ref: SNO) and HTN (HR:2.34, HR:2.31; Ref: SNO) without significant difference in variability of obesity/metabolic health, moreover, for incident CVD and CKD among obesity status only SO (HR:1.24, HR:1.55, respectively, Ref: SNO) had significant association compared to reference SNO, and variability in obesity/metabolic health among obesity status had only significant difference for VNO vs SNO in incident obesity, metabolically unhealthy state, DM and HTN but not CVD and CKD. Among metabolic health status VMH (Variable metabolic health, metabolically-healthy) vs SMH (Stable metabolic health, metabolically-healthy) had no significant association with either of 6 incident outcomes yet metabolically unhealthy patients compared to SMH (Stable Metabolic Health) either of stable metabolically unhealthy (SMU) and variable metabolically unhealthy (VMU) both associated with higher incident obesity (HR:1.66, HR:2.23, respectively), DM (HR:3.24, HR:2.44, Ref: SMH), CVD (HR:1.74, HR:2.24, Ref: SMH) and CKD (HR:1.50, HR:2.06, Ref: SMH), and for incident HTN only SMU (HR:1.61, Ref: SMH) had significant associations, but among metabolic health status only significant difference for variability in obesity/metabolic health was for SMU vs VMU in incident CVD, and for CKD variability in either of obesity and metabolic health had no significant difference. All models were adjusted with age, gender, examination cycle, education, smoking and physical activity.”.  

**Addendum Section 9.5.2:**

• On the page-10 at the paragraph-5 for the associations across AIP (Atherogenic Index of Plasma) tertiles model adjustment weren’t termed. The paragraph was edited as follows:
“J. S Nam [153] including 9581 Korean adults with average follow-up of 4.2 years demonstrated across increasing baseline Atherogenic Index of Plasma (AIP) Log_10_[TG/HDL-c] tertiles from T1 to T3 in univariate model significantly associated with increased Δ√Ln[CAC], annualized Δ√Ln[CAC] and higher rate of CAC-progression (either first non-zero CACs or increase of ≥2.5 units of Δ√Ln[CAC] over baseline) during the follow-up, moreover, for CACs progression the trend remained significant after adjustment for age and gender yet in additional adjustment with BMI, SBP, FPG, LDL-c, physical activity, alcohol, smoking, DM, HTN and baseline Ln[CACs + 1] this association attenuated.”.
• On the page-10 at the paragraph-7 for the associations of high vs low TG/HDL-c with atherosclerotic changes model adjustments weren’t termed yet remains significant after reported model adjustments. The relevant paragraph section was edited as follows:
“reported in univariate model High TG/HDL vs Low TG/HDL by cut off of 2.45 significantly associated with higher CACs (29.15 vs 0.0), higher prevalence of non-zero CACs > 0 (64.5% vs 45%) and higher percentages of 2 (29.5% vs 21.5%) or 3 (32.7% vs 20.9%) different vascular sites (spanning coronary, carotid and femoral arteries) with atherosclerotic changes, and these associations remained significant after model adjustment for age, gender, HbA1c, FPG, smoking, SBP, BMI and hs-CRP.”.  

**Corrigendum Section 9.5.3:**

• On the page-11 at the paragraph-1, on the relations of VAT (≥100 vs <100) and CACs (≥10 vs <10) with adipocytokines VAT ≥ 100 (vs VAT <100) likewise CACs ≥ 10 (vs CACs < 10) was realized unwittingly noted in null associations for adiponectin and leptin despite noted positive and inverse associations sequentially for leptin and adiponectin. Author apologizes for any inconvenience and the relevant paragraph section was edited additively with including these delineations also nonsignificant associations as follows:
“(…), in univariate models demonstrated VAT ≥ 100 vs <100, CACs ≥ 10 vs CACs < 10 and Diastolic dysfunction presence vs absence all significantly associated with older age, greater WC in men and lower eGDR (estimated-Glucose Disposal Rate), higher CACs yet all had no significant association with HbA1c (%), HbA1c (mmol/mol), DBP, TC, TG and IL-6 levels, smoking and presence of microvascular abnormalities of microalbuminuria and polyneuropathy; moreover, both VAT ≥ 100 vs <100 and CACs ≥ 10 vs <10 significantly associated with male gender, higher BMI, WC in both genders, SBP, lipid-lowering and antihypertensive drugs use, greater rate of having metabolic syndrome and lower GFR yet both nonsignificant for adipocytokine of IL-6, and besides, CACs (≥10 vs <10) was nonsignificant for TNF-α, adiponectin and leptin levels, too; however, only VAT ≥ 100 vs <100 (not CACs ≥ 10 or diastolic dysfunction) significantly associated with lower HDL-c, eIS (estimated-Insulin sensitivity) and adiponectin levels besides higher TNF-α and Leptin levels; nonetheless, both VAT ≥ 100 vs <100 and Diastolic dysfunction presence vs absence significantly associated with higher insulin level (yet borderline significance for patients with diastolic dysfunction, p = 0.053) and rate of having CACs ≥ 10; moreover, only CACs ≥ 10 vs <10 (not VAT ≥ 100 or Diastolic dysfunction) significantly associated with higher levels of LDL-c, rates of macrovascular abnormalities of carotid artery plaques and wall-motion abnormalities, and microvascular abnormality of retinopathy, in addition, CACs ≥ 10 also significantly associated with higher VAT, greater rates of VAT ≥ 100 and longer diabetes duration (in years), and LASSO (Least Absolute Shrinkage and Selection Operator Logistic Regression) identified age, diabetes duration and LDL-c as best predictors of CACs and additional to these factors male gender, use of lipid lowering drugs and MetS were revealed independent predictors of CACs ≥ 10 by multivariable logistic regression; furthermore, for VAT linear regression analysis demonstrated eIS was the only independent predictor of Log[VAT], and for diastolic dysfunction age and WC were revealed independent predictors, and then for diastolic dysfunction LASSO revealed diabetes duration had highest predictive value among cardiovascular risk factors reported within this study, in summary these findings may signify the importance of earlier glycemic control.”.  

**Addendum Section 9.5.3:**

• On the page-11 at the paragraph-5, for the absolute change in CACs during the follow-up period Δ (delta) sign of difference were added with revealing ORs for these associations within the relevant paragraph section as follows:
“(…) HbA1C of < 7.0% compared to non-OGC group as HbA1c ≥ 7.0% significantly associated with lower annual increase in CAC score (√Transformed) in univariate model, and lower prevalence of CAC progression as square-root of difference in CAC within inter-scan period becoming ≥ 2.5 and lower risk of progressing CAC in follow-up period exceeds with ΔCAC > 200 (OR:0.66) and ΔCAC > 300 (OR:0.62) in multivariable adjusted models (of age, gender, BMI, smoking, HTN, Dyslipidemia and baseline CACs categories).”.
• On the page-12 at the paragraph-1 to contribute the further discussions across the study unshown OR for the association of TyG-BMI Q4vsQ1 with CACs progression and the association of TyG Q2vsQ1 with CACs progression were added at the relevant paragraph section as follows:
“(…) only TyG-WC could significantly associated with CAC-progression for both Q4vsQ1 (OR:1.66) and Q3vsQ1 (OR:1.64), TyG-BMI could have significant association for only Q4vsQ1 (OR:1.62) and TyG had significant association only for Q2vsQ1 (OR:1.65); (…)”  

**Corrigendum Section 9.5.4:**

• On the page-12 at the 2’nd paragraph the inverse association of E/A index with MetS categories was unwittingly noted just oppositely as positive association, though the inverse association was noted just subsequently within the paragraph as in group 3 vs group 1 by multivariable logistic regression. The relevant clause was edited with removed hyphen between group 2 and 3 as follows:
“(…) group 2 or 3 vs group 1 ETT measured exercise duration (s), METs and E/A (ratio of peak early-diastolic to late-diastolic transmitral flow) decreased yet LCXA-CACs, RCA-CACs and echocardiography defined LVEDD (Left ventricular end-diastolic diameter, mm) and A (Peak late-diastolic transmitral-flow, cm/sec) increased by one-way ANOVA; (…)”.  

**Corrigendum Section 9.5.5:**

• On the page-12 at the 3rd paragraph muscle metabolite of Histidine was realized unintentionally reported among 10 metabolites directly associated with Ln[CAC + 1] rather than one of the metabolites inversely associated with Ln[CAC + 1], in spite of noted strong inverse association of histidine with log-transformed CACs below the paragraph. With additional reveals for the model adjustments in these significant associations, the relevant clauses starting with the 16th line were edited as follows:
“(…) the study reported in model-2 (adjusted for the conventional risk factors of model-1: age, gender, ethnicity + model-2: cohort, LDL, HDL, SBP, DM, smoking status and use of lipid or BP lowering drugs) metabolites directly associated with Ln[CAC + 1] were Alanine (mitochondrial metabolism, TCA cycle, aerobic energy metabolism), Glycine (1-carbon metabolism, Amino acid), Methionine (Sulphur Metabolism, Amino acid), 4 Polyol and Carbohydrate Metabolism metabolites (D-Glucose, 1,5-Anhydrosorbitol[insulin resistance], Mannose[prediabetes, T2DM and pro-inflammatory associations, lipoprotein glycation], Myoinositol), Acetyl Glycoproteins (secreted in pro-inflammatory state), Glycerol (Lipid and Fatty Acid Related Metabolism) and Acetaminophen-Glucoronide, while again in adjusted model-2 Ln[CAC + 1] inversely associated with Histidine (Muscle Metabolism) and 9 metabolites, which are 2 metabolites involve in regulation of [Glutathione] concentration (Glutamate and 5-oxoproline[Urea Cycle Metabolism]), Glutamine (Mitochondrial Metabolism), N,N-Dimethylglycine (1–carbonmetabolism), Lysine (Mitochondrial Metabolism), Phenylalanine (aromatic Amino acid), 3-hydroxybutyrate (Mitochondrial Metabolism), Citrate (TCA cycle) and Albumin; moreover, after further adjustment for CVD risk factors (model-1 + model-2) across pooled 3 cohorts based on permutation of spectral features in random allocation of CACs (Ln[CACs + 1]) outcomes to each study participants as MWAS (Metabolome Wide Association Studies) below Metabolome Wide Significance Level (MWASL) threshold (p = 2.4 * 10E–9 to 1.1 * 10E–5) with Ln[CACs + 1] mannose, alanine and acetaminophen-glucuronide strongly associated yet glutamate and histidine had strong inverse associations besides other significant metabolites also remained nominally significant (p < 0.05) but by 50% weaker association; furthermore, lipoprotein subcategories of total plasma cholesterol, total plasma apolipoprotein B and apolipoprotein B within total plasma LDL significantly associated with Ln[CACs + 1] in model-3 (further adjusted for model-1 + nonlipid CVD risk factors of SBP, DM, Smoking status, and use of medication for hypercholesterolemia, BP or DM).”.  

**Addendum Section 9.5.7:**

• On the page-13 at the paragraph-5 adjusted covariates of models for the associations of cholesterol synthesis and absorption markers with CACs > 0 vs. CACs = 0 weren’t revealed. The relevant paragraph section was edited as follows:
“(…) illustrated 3‘rd vs 1‘st tertile both cholesterol synthesis marker desmosterol (OR:3.241, 95% CI:[1,700–6.179]; rather than lathosterol) and cholesterol absorption marker campesterol (OR:1.858, 95% CI:[1.020–3.387]; rather than sitosterol) significantly associated with having a CACs > 0 in multivariable models (adjusted for Age, BMI, WC, TC, HOMA-IR, HTN and smoking) (…)”.  

**Corrigendum Section 9.5.7:**

• On the page-13 at the paragraph-7 extending to the page-14 the insignificant associations of discordant Apo-B to non-HDL-c was noted slightly different as interchangeably with HDL-c. Author apologizes for any inconvenience. The relevant 2 sections were edited as follows:
“A study by Cao et al. [170] including 4623 participants with MESA-registry along 8.8 years of median follow-up and reporting ApoB level discordance with LDL-c and non-HDL-c (quantified by subtracting HDL-c from TC), which is ApoB levels more/less than expected or residuals in linear regression models determined by LDL-c or non-HDL-c defined as (…)”.
“(…) discordant high Apo-B to non-HDL-c directly significantly associated yet in fully-adjustment model any discordant Apo-B to either LDL-c or non-HDL-c remained similar, (…)”.  

**Corrigendum Section 9.5.9:**

• On the page-14 at the 5th paragraph a typing error noting “quartiles” rather than “quantiles” was realized, and the relevant clause was edited as follows:
“(…) in analysis combining 2‘nd to 4‘th Quantiles as reference group female patients on contrary to males had significant U-shaped association (males only 1’st Q) yet (…)”.
• On the page-15 at the 1’st paragraph the inverse association between serum Magnesium quartiles and non-zero CACs > 0 was noted for the reviewed study within the paragraph, but for per 1-SD serum Magnesium increments the inverse character and OR were unwittingly unclarified. Author apologizes for any inconvenience. The relevant clause was edited as follows:
“(…) and again per 1-SD (0.17 mg/dL) increment of serum magnesium concentration significantly associated with lower rate of any non-zero coronary artery calcification (CACs > 0, OR:0.84) in multivariable adjustment.”.  

**Addendum Section 9.5.10:**

• On the page-15 at the paragraph-4 model adjustments and units of parameters in associations of 1-SD of FGF-21 level with baseline CACs and of CVD/CHD-endpoints weren’t shown, and in accordance the relevant paragraph section was edited as follows:
“moreover, FGF-21 level had graded significant response across quartiles for non-zero CACs > 0 vs CACs = 0 at baseline, and consistently association of each 1-SD of FGF-21 level (1.36 units in Ln-transformed FGF-21) with baseline non-zero CACs > 0 vs CACs = 0 (but not baseline continuous absolute Ln-transformed FGF-21 among CACs > 0) remained significant for adjustment of age, gender, race/ethnicity, family income, physical activity and education in model-1, however, couldn’t remain significant with baseline through either CACs > 0 or Ln[CACs] for further adjustments in either model-2 (model-1 + BMI, HR, use of lipid-lowering medication, HDLc, Ln[TG], Ln[HOMA-IR], eGFR, smoking status, pack-years of smoking, current alcohol intake, DM, SBP and use of antihypertensive medications) or model-3 (model-2 + CRP, Fibrinogen and IL-6); furthermore, non-linearly for FGF-21 ≥ 80.0 mg/dL vs FGF-21 < 80.0 mg/dL significantly associated with hard-CVD in model-1 (HR:1.34, 95% CI:1.12–1.60) and in borderline significance in model-2 (HR:1.20, 95% CI:1.00–1.45) besides hard-CHD in model-1 (HR:1.46, 95% CI:1.19–1.80) and model-2 (HR:1.26, p = 0.045) yet in model-3 no significant association was observed for both endpoints of CVD and CHD.”
• On the page-15 at the 7th paragraph hazard ratios at the comparisons of low adiponectin T1 vs T2-3 and high leptin T3 vs T1-2 for CACs > 0 and CACs > 100, respectively, were not shown. To contribute to the further discussions in the study HR were added at the relevant clauses as follows:
“(…) in fully adjusted model 2 when stratified by genders low adiponectin T1 vs T2-3 among female inverse association with CACs > 0 presented (HR:0.32, CI: 0.07–0.83) and (…)”.

“(…) when 243 statin users were excluded inverse association of high leptin T3 vs T1-2 with CACs > 100 (HR:0.24, CI: 0.13–0.81) among males became available (…)”.

• On the page-16 at the 1’st paragraph, the common threshold value of Log-transformed CAC Volume score for the definition of the CACs progression wasn’t shown, and the relevant clause was edited as follows:
“(…) after inter-scan period square root transformed-CAC volume score difference ≥ 2.5 or progression significantly inversely associated with only adiponectin among baseline adipokine measurements at each SD increase in adjusted model 5 (OR:0.68, 95 %CI:0.51–0.92) (…)”.
• On page-16 at paragraph-2 through the end of first column beta-coefficient indices for the strength of associations by linear regressions and the odds ratios for the associations of GlycA increments with prevalence of atherosclerosis (termed by plaque feature) weren’t shown. The relevant paragraph section was edited with these elaboration as follows:
“(…) furthermore, according to Poisson-regression analysis GlycA levels per 1-SD increase (without significant interaction of HIV-serostatus) significantly associated with CACs till highly adjusted model-4 (for demographics[model-1] + traditional risk factors[model-2] + inflammatory markers[model-3] and + monocyte activation markers) but not model-5 (model-4 + HIV-related factors) among overall-cohort (OR:1.09) and both HIV-serogroups, coronary stenosis ≥ 50% in model-3 among overall-cohort (OR:1.18) and model-4 among HIV-uninfected participants (OR:1.53), Calcified Plaques in model-4 among both overall-cohort (OR:1.15) and only HIV-uninfected participants (OR:1.22) and mixed plaques only in model-1 among overall-cohort (OR:1.12) yet GlycA-level per 1-SD increment remained similar for any plaque types and noncalcified plaques; in addition, among individuals with plaque score > 0 each 1-SD increase of GlycA levels significantly associated with transformed Ln[CACs] till model-3 adjustment in overall-cohort (β: 0.18), Ln[Total-Plaque score] till model-4 adjustment (β: 0.16) with stronger association among HIV-uninfected vs HIV-infected serogroups (β: 0.22 vs 0.10 in model-4, p-interaction:0.003), NCP-s (Non-calcified Plaque score) only in model-1 adjustment for HIV-uninfected serogroup (β: 0.13) without significant serogroup interaction (p-interaction:0.13) and MP-s (Mixed Plaque Score) till model-4 adjustment in overall-cohort (β: 0.15) with comparable stronger association for HIV-uninfected serogroup than whole cohort till model-2 adjustments (p-interaction: 0.03, all p for interactions in model-2 adjustments, β: 0.22 vs 0.12), but remained similar for Ln[Calcified Plaque score] in any adjusted-models.”  

**Corrigendum Section 9.5.13:**

• On the page-17 at the 5th paragraph an incomplete phrase was noticed, and the relevant clause was edited as follows:
“(…) while sarcopenia group had highest mean-age, highest prevalence of DM, highest level of FPG, and lowest hs-CRP and creatinine levels; (…)”  

**Addendum Section 9.5.13:**

• On the page-18 at the paragraph-2 positive association between RSFV/BMI and CACs > 10 (vs. CACs ≤ 10) was noticed may be interpreted as inverse association likewise eGFR. The relevant paragraph section was edited with additional analyses as follows:
“(…) reported in unadjusted models age, male gender (exceptionally by Chi-square test) and focal adipose tissue accumulation indices of RSFV (right Renal Sinus Fat Volume, cm3), RSFV/VATV ratio (RSFV to Visceral Adipose Tissue Volume), Log_10_[RSFV/VATV], V/S ratio (Ratio of Visceral Adipose Tissue area to subcutaneous adipose tissue area) and RSFV/BMI ratio positively but eGFR inversely associated with higher prevalence of CACs > 10 vs. CACs ≤ 10 (presence vs. absence of CAC) among both overall-study population and middle-aged participants by Student‘s *t*-test but not for elderly participants, moreover, CACs significantly correlated with only RSFV/VATV ratio index rather than RSFV, VATV, V/S ratio and RSFV/BMI ratio among study population (r = 0.23) and middle aged participants (r = 0.42) but no significant association for elderly, and relevantly in study population in univariate models RSFV/VATV ratio significantly correlated with pack-years of smoking (r = 0.18, but not for middle-aged or elderly participants; in contrast V/S-ratio for both groups and RSFV and VATV for elderly group correlated significantly), TG (r = −0.20, excepts middle-aged participants, yet VATV for both groups and V/S ratio for elderly group correlated), LDL-c (r = −0.22, excepts elderly) and BMI (r = −0.27, excepts elderly, but RSFV and VATV correlated for both groups); furthermore, by multivariable logistic regression analysis adjusted for conventional risk factors (age, gender, pack-years of smoking, DM, HTN, BMI, kidney volume[sign of renal functional impairment], BMI) among middle-aged participants Log_10_[RSFV/VATV] rather than RSFV and VATV significantly associated with greater prevalence of CACs > 10 vs CACs ≤ 10 (OR:15.9) and also RSFV/VATV ratio with absolute CACs (β:0.31, Coefficient:141.3) significantly associated regardless of conventional risk factors, and among elderly participants none of the focal adipose tissue accumulation indices (RSFV, VATV, RSFV/VATV, V/S-ratio and RSFV/BMI) had significant association; and these findings may suggest RSFV/VATV ratio index intricately linked with metabolic syndrome besides RSFV (also VATV) impairing renal functions, and RSFV/VATV could alternatively sign early coronary artery calcification.”.  

**Erratum Section 9.5.13:**

• On the page-17 at the paragraph-2 the link for cited reference study 187 through its doi number appeared not working due to an extra dot at the end of string. The doi number for cited reference study 187 as “https://doi.org/10.1371/journal.pone.0196328.” was edited into “https://doi.org/10.1371/journal.pone.0196328”
• On page-17 at the paragraph-3 through the final lines some editing notes added to manuscript during proof editing phase were unintentionally remained in the text. The relevant clause was edited as follows: “(…) moreover, according to multivariate logistic regression analysis EAT volume (per 10 cm^3^, OR:1.13), age (per year, OR:1.08), cumulative pack-years of tobacco use (OR:1.03), retinopathy (OR:1.89) and macrovascular disease (OR:3.94) significantly associated with CACs ≥ 100.”  

**Addendum Section 9.5.14:**

• On the page-18 at the 3rd paragraph significant trend of NLR across CACs categories wasn’t illustrated, and in accordance the relevant clause was edited as follows: “(…) within stratification by CACs-categories (CACs 0, Mild: CACs 1–100, Moderate: CACs 101–400 and Severe: CACs > 400) NLR had significant positive trend yet hematological tests of WBC-counts, absolute and relative neutrophil or lymphocyte counts and Platelet-absolute count remained similar, (…)”
• On the page-18 at the 5th paragraph among Quintiles the lowest cumulative incidence at the Q2 wasn’t shown. The relevant clause was edited as follows:
“(…) subsequent to 4‘th year Q5 overcome the minor difference with Q4 and toward the year of 10 stratification of cumulative incidence rates by increasing P-selectin Quintile categories became visible exceptionally for Q2 cumulative incidence rate remained lower than other Quintiles.”  

**Addendum Section 9.5.15:**

• On the page-19 at the 2’nd paragraph the strengths of associations between miRNA-8059 and gene pathways by P-values according to Kyoto encyclopedia of genes and genomes weren’t shown. To reveal the strengths of associations the relevant clause was edited as follows:
“(…) additionally according to DIANA Tools miRPath modulated mRNA pathways by miRNA-8059 were estimated as metabolism of xenobiotics by cytochrome P450 (p = 1.34E-20), biosynthesis of unsaturated fatty acids (p = 6.66E-11), fatty acid metabolism (p = 3.44E-06), chemical carcinogenesis (p = 0.0023), glycosaminoglycan biosynthesis-keratan sulfate (p = 0.0034), glycosaminoglycan degradation (p = 0.013), nucleotide excision repair (p = 0.013), glycosphingolipid biosynthesis (p = 0.015) and proteoglycans in cancer (p = 0.018).”  

**Addendum Section 9.5.16 Fatty Liver Disease:** Under section 9.5 Metabolic Health the subsection on fatty liver changes in association with subclinical cardiovascular disease progression was realized absent from the study text. Its absence wasn’t noticed at final edits. Since this section was typed at separate file and folder, somehow it was forgotten at the submission. The author apologizes for any inconvenience with this lacking section. The references for this section are listed as in **Addendum References**. The added subsection as follows:  

**Addendum Section 9.5.16. Fatty Liver Disease (FLD)**

**Addendum Section 9.5.16.1 NAFLD and Gender**

A cross-sectional study by Wu et al. [1](228) including 2345 subjects with mean age of 55.66 ± 7.79 years examining relationships between NAFLD (indicated by ultrasonography), subclinical atherosclerosis (of any CACs > 0) and the risk of CVD (defined by the CACs categories at thresholds of 0, ≤100, ≤400 and >400), reported participants with NAFLD vs without NAFLD significantly associated with greater prevalence of having CACs > 0 (OR:1.348) and higher CACs categories (OR:1.359) in fully-adjusted model (of without any adjustment in model-1 + additionally adjusted for model-2: age and gender + model-3: smoking status, HTN, DM + model-4: Hypercholesterolemia, LDL-c, physical activity, level of education and income) yet in gender stratification males (n = 1035) have no significant association between NAFLD and either CACs > 0 or CACs categories even for crude models, and comparably females (n = 1310) had more significant trends and associations, in which for association of higher CACs > 0 prevalence remained significant till model-3 adjustment (OR:1.568) and borderline significant for model-4, and for CACs categories remained significant even after model-4 adjustment (OR:1.526), moreover, among female subjects postmenopausal (n = 802) vs. premenopausal (n = 508) patients had significantly higher levels of BMI, abdominal circumference, hip circumference, TG, TC and LDL-c, and higher prevalence of DM, HTN, Hyperlipidemia, NAFLD and any CACs > 0.  

**Addendum Section 9.5.16.2 NAFLD and Plaque Features**

A general health examination cohort study by Lee et al. [2](229) including 5121 asymptomatic Korean adults aged mean 53.8 years to evaluate effects of NAFLD, which was detected with acquired images in ultrasonography and evaluated in terms of hepatic steatosis and fibrotic changes by FLI (Fatty liver index) and NFS (NAFLD fibrosis score) respectively, on the subclinical coronary atherosclerosis, which was defined by CACs and plaque compositions on contrast using Coronary CT-angiography (CCTA), demonstrated those with NAFLD vs without NAFLD significantly associated with higher prevalence of any atherosclerotic plaque (47.9% vs 36.2%, OR:1.62), calcified plaque (31.2% vs 24.0%, OR:1.44), non-calcified plaque (22.2% vs 15.9%, OR:1.50), mixed plaque (10.3% vs 6.4%, OR:1.52) and significant stenosis (10.3% vs 6.4%, OR:1.68) and higher mean CACs (46.0 vs 33.2, and CACs > 10 vs CACs = 0 OR:1.49) by both chi-square test and logistic regression of univariate model (reported respectively) besides higher CACs categories by chi-square test, however, in multivariate logistic regression adjusted for covariates of age, gender and CV-risk factors (of obesity, DM, HTN, Hyperlipidemia, current smoking, FH-CAD, hs-CRP ≥ 2 mg/L) associations of NAFLD with greater prevalence of CACs > 10, calcified plaque and mixed plaque attenuated but remained significant to any atherosclerotic plaque (OR:1.18, 95% CI:1.03–1.35), non-calcified plaque (OR:1.27, 95% CI:1.08–1.48) and significant stenosis (OR:1.28, 95% CI:1.02–1.61), moreover, in multivariate logistic regression models with same covariate adjustments stratifications by FLI ≥ 30 vs FLI < 30 significantly associated with any atherosclerotic plaque (OR:1.22, 95% CI:1.04–1.44) and non-calcified plaque (OR:1.37, 95% CI:1.14–1.65) yet stratification by NFS ≥ −1.455 vs NFS < −1.455 significantly associated with only non-calcified plaque (OR:1.20, 95% CI:1.08–1.42); in summary, these findings underlie effects of NAFLD or NAFLD severity by FLI or NFS on subclinical atherosclerosis mediated independent of traditional risk factors by increased non-calcified plaques, which is less stable, prone to rupture, and as discussed by authors suggestive for explaining sudden and unexpected CVE in asymptomatic patients with NAFLD, which necessitates early identification and interventions through lifestyle modification and medical treatments for NAFLD.  

**Addendum Section 9.5.16.3 NAFLD and MetS-Components**

A follow-up study by Cho et al. [3](230) involving asymptomatic 1173 Korean participants aged mean 54.1 with mean follow-up interval of 3.0 (2.0–3.8) years within 4 sub-categories based on presence or absence of NAFLD defined by ultrasonographic features and MetS by NCEP-ATP 3 criteria, demonstrated at baseline in multiple-logistic regression analysis of model-3 (adjusted for model-1: age, gender, BMI + model-2: smoking, drinking, exercise habits + model-3: follow-up interval period, LDL-c and hs-CRP) among 4 subgroups compared to reference category of NAFLD(−)MetS(−) participants with both abnormalities of NAFLD(+)MetS(+) but not either NAFLD(+)MetS(−) or NAFLD(−)MetS(+) significantly associated with baseline CACs > 0 vs CAC = 0 (OR:1.45, 95% CI:1.00–2.11, borderline significance), and after follow-up interval at the second examination CACs-progression, which was defined by either incident CACs > 0 or the increase of ≥2.5 units in square-root transformation of the CACs (√CACs) between two examination, significantly associated with older age, male gender, greater BMI, higher WC, SBP, DBP, FPG, HbA1c, TG, Uric acid, AST, ALT, GGT, FRS, baseline CACs, baseline CACs category, CACs at second examination and follow-up interval (years), lower HDL-c, less likely physically active (≥2-–3 times/week, borderline significance p = 0.088) and higher prevalence of current smoker, moderate drinker, DM and HTN; moreover, by multiple-logistic regression analysis in further adjusted model (of model-3 + baseline CACs) compared to reference category NAFLD-only participants (NAFLD(+)MetS(−), OR:1.53, 95% CI:1.05–2.23) and participants with both syndrome (NAFLD(+)MetS(+), OR:1.76, 95% CI:1.18–2.62) significantly associated with CACs progression but not for NAFLD(−)MetS(+); in summary significant CACs progression for NAFLD regardless of MetS may necessitate detection and treatment of NAFLD among metabolically healthy patients despite its insignificant association with baseline CACs but since atherosclerosis is a dynamic and longitudinal process monitored CACs progression may have greater prognostic value for patients’ future CVE risk than baseline CACs, and as authors discussed since both diseases share common pathophysiology (such as IR, ongoing tissue injury, low level chronic systemic inflammation, oxidative stress, endothelial dysfunction, hypercoagulable and prothrombotic state, central obesity, dyslipidemia, hypertension and dysglycemia) similar therapeutic approaches (life-style interventions, physical activities, weight loss, and medical therapies to the metabolic risk factors of hypertension, atherogenic dyslipidemia and dysglycemia) to both diseases might be effective.

A cross-sectional population-based study by Gummesson et al. [4](231) including 1015 subjects with mean 57.6 years of age into analysis whereas excluding patients with hepatic steatosis other than NAFLD secondary to chronic hepatitis, alcohol-induced liver disease, self-reported high alcohol consumptions out of daily limits, and use of oral corticosteroids, methotrexate or amiodarone to examine the associations between NAFLD-status indicated by liver CT and ASCVD defined by CACs, Carotid plaques and IMT, and besides for these associations the potential modifying effects of 7 metabolic risk factors, which includes Diabetes status (no diabetes, IFG and diabetes), HTN, LDL-c ≥ 4 mmol/L, HDL-c < 1.3 or 1.0 mmol/L, TG ≥ 1.7 mmol/L, hs-CRP ≥ 5 mg/dL and insulin ≥ 20 mU/L, potential confounders, which includes age, gender, education level, smoking status, daily alcohol intake, physical activity defined by % of the time in accelerometer measured moderate-to-vigorous physical activity (MVPA), sedentary time %, BMI, and gender specific Waist-circumference and Visceral Fat Area quartiles, and propensity score, which was derived from multivariable logistic regression model between NAFLD and potential confounders and was used to adequately eliminate as balancing the distribution of the observed confounders among participants with or without NAFLD for transparently designing and analyzing the observational data, reported those with NAFLD vs. without-NAFLD significantly associated with male gender (OR:3.16), greater BMI severity (kg/m^2^; within BMI 25–30 OR:6.15 and for a BMI ≥ 30 OR:10.3), higher quartiles of Visceral Fat Area (cm2; compared to 1st and 2nd quartile [M:0–195, F:0–116] 3rd Q [M:195–256, F:116–165] OR:4.79 and 4th Q [M:>256, F:>165] OR:9.86) in multivariable model, and having CACs > 0 (OR:2.11, 95% CI:1.32–3.39) but not with carotid plaques > 0 and cIMT in propensity score adjusted model and again association of NAFLD with CACs > 0 remained significant after further adjustment in model-3 (Propensity score model + for 7 metabolic risk factors; OR:1.77, 95% CI:1.07–2.94); furthermore, in subgroup analysis stratifying associations between NAFLD and CACs > 0 in model adjusted by propensity score at each 7 metabolic risk factors the association strengthened in the absence of each risk factor as No-diabetes vs IFG (OR:2.72 vs nonsignificant), No-HTN vs HTN (OR:3.53 vs nonsignificant), LDLc < 4 vs ≥4 mmol/L (OR:2.37 vs nonsignificant), HDLc ≥ 1.3/1.0 vs <1.3/1.0 (F/M, OR:2.79 vs nonsignificant), TG < 1.7 vs ≥1.7 mmol/L (OR:4.46 vs nonsignificant), hsCRP < 5 vs ≥5 mg/L (OR:2.53 vs nonsignificant) and insulin < 20 vs ≥20 mU/L (OR:2.27 vs nonsignificant), in addition, at categories of fewer number of metabolic risk factors stronger association of NAFLD to CACs > 0 exist as 0–1 risk factor at OR:5.94 (2.13–16.6), 2–3 risk factors at OR:2.33 (1.12–4.87) and nonsignificant at 4–7 risk factors; in summary, these findings may suggest NAFLD can predict CACs independently from classical metabolic risk factors, which are commonly associated with obesity, MetS or ASCVD, particularly among metabolically healthy patients by classical risk factors, and authors discussed the necessity for the broader range potential mediators alternative to conventional risk factors to explain the association of NAFLD with CAC for future studies.

A cross-sectional study by Kwak et al. [5](232) including 213 participants from 1750 recruited subjects with health screening at mean age of 59 years with indication of diabetes by either having FPG ≥ 126 mg/dL or HbA1c ≥ 6.5% into analysis, whereas excluding patients with chronic liver disease with cause of other than NAFLD, a history of symptomatic CVD and use of medications causing steatosis, to examine the association between NAFLD and CACs, reported participants with NAFLD vs without-NAFLD had significantly higher levels of median FPG (138 vs 130 mg/dL), TG (135.5 vs 92 mg/dL), ALT (33 vs 22 IU/L) and GGT (38.0 vs 28.0 IU/L), higher prevalence of MetS (68.6% vs 36.6%) and HTN (66.3% vs 48.0%), and greater BMI-median (26.2 vs 24.1 kg/m^2^) and WC-median (92 vs 87.5 cm), moreover, across HbA1c quartiles dose–response interaction of strengthening association was observed for the relation between NAFLD and Ln[CACs + 1], besides, in multivariate linear regression analysis (adjusted for covariates of age, gender, HTN, BMI, WC, HDL-c, TG) HbA1c ≥ 7% vs <7 significantly interacted with the association between NAFLD and Ln[CACs + 1], that in higher HbA1c ≥ 7% category NAFLD significantly associated with Ln[CACs + 1] independent of the covariates; in summary, these results may suggest sustained hyperglycemia can modify effects of NAFLD on CACs and stringent control of hyperglycemia simultaneously with intensive management of NAFLD might be effective to prevent ASCVD, and authors discussed the role of IR aggravated by poor glycemic control, which associates with oxidative stress, pro-inflammatory state, hypoadiponectinemia and abnormal lipoprotein metabolism, in the relation of NAFLD and CACs under hyperglycemic conditions.  

**Addendum Section 9.5.16.4 NAFLD and Obesity**

A retrospective follow-up study of occupational health check-ups by Kim et al. [6](233) recruiting 1575 young Korean participants aged mean 39.9 ± 5.4 years of age with consecutive examinations over period of 4 years into 4 subcategories based on baseline dichotomies of NAFLD presence or absence (indicated by ultrasonography with 4 features of hepatorenal contrast, liver brightness, deep attenuation and vascular blurring) and systemic inflammatory biomarker of hs-CRP at ≥0.06 mg/dL or <0.06 mg/dL by median threshold, showed both baseline NAFLD(+) vs NAFLD(−) and development of any CACs > 0 incidence during follow-up significantly associated with older age, male gender, conventional risk factors and IR (by HOMA-IR), furthermore, intricately CACs > 0 incidence significantly associated with baseline NAFLD(+) vs NAFLD(−) in incrementally increasing severity by hs-CRP at ≥ 0.06 mg/dL vs <0.06 mg/dL, besides significant trend of increasing CACs > 0 incidence across from NAFLD(−)hs-CRP < 0.06 mg/dL to NAFLD(+)hs-CRP ≥ 0.06 mg/dL, moreover, in multivariate logistic regression analysis of model 3 (adjusted for age, gender, ALT, smoking, FPG and LDL) at baseline categories only those with NAFLD(+) and hs-CRP ≥ 0.06 mg/dL vs reference category of NAFLD(−) overall hs-CRP or hs-CRP < 0.06 mg/dL significantly associated with CACs > 0 incidence (OR:1.67, 95% CI:1.01–2.77) yet no other 2 subgroups compared to reference category but after further adjustment beyond model 3 with BMI also attenuated this only association, which indicates effect of NAFLD on CACs is more like an obesity-related epiphenomenon than systemic inflammation assessed by hs-CRP, which could also be sourced by obesity, so dysfunctional adipose tissue along with visceral obesity and the accumulation of ectopic fat could mediated subclinical atherosclerosis as authors discussed.

A cross-sectional study by Lee et al. [7](234) including 21,335 Korean male adults aged mean of 41 years (22–88 years range) categorized into 4 subgroups upon presence or absence of NAFLD, which was indicated for participants with fat accumulation of ≥5% of liver-weight and daily alcohol intake < 20 g/day, and presence or absence of abdominal obesity defined by Weight-to-Hip ratio (WHR) threshold of 0.9, illustrated significant trend of worsening metabolic parameters across subgroups of NAFLD(+) vs NAFLD(−), which was aggravated by abdominal obesity within category, for TC, TG, HDL-c, LDL-c, AST, ALT, γ-GTP, rate of doing regular exercise, prevalence of ever smoked, HOMA-IR, HbA1c and prevalence of DM, exceptionally for rate of statin use, SBP and HTN prevalence abdominal obesity only participants compared to NAFLD-only had worser values but again participants with both disorder had worst, besides participants with either condition had similar FBS and hs-CRP, moreover, for age-adjusted mean Ln[CACs + 1] participants with both abnormality had highest level (0.624) and participants without either abnormality had lowest level (0.416) yet participants with either NAFLD-only or abdominal obesity only remained similar to each other (0.490 and 0.488, respectively); furthermore, in multivariable analysis for CACs > 0 independent variables of NAFLD vs without (OR:1.36) and abdominal obesity vs without (OR:1.22) significantly associated in adjusted model-2 (model-1:age + DM, HTN, smoking and physical activity) yet further adjustment in model-3 (model-2 + total cholesterol, HDL-c and HOMA-IR) only NAFLD(+) vs NAFLD(−) could remained significant (OR:1.16); in addition, in multivariable analysis comparing 3 subgroups with reference group without either disorders in only age adjusted model 3 subgroups had significant association yet further adjustment in model-2 attenuates abdominal obesity only group then in fully adjusted model-3 only subgroup of both NAFLD and abdominal obesity remained significant (OR:1.19); in summary, as authors discussed these analysis may suggest for CACs NAFLD had greater relevance than abdominal obesity independently from MetS and IR, conducive role of insulin-resistance and blood lipid profile to the effects of NAFLD on CACs but especially effects of abdominal obesity on CACs may signify the role of VAT (such as releasing pro-inflammatory adipocytokines and free fatty acids acting on local-milieu and distant tissue sites) on both hepatic fat accumulation through the intricacy of NAFLD with MetS as the manifestation in liver and the CAD, and the synergistic interaction of NAFLD and abdominal obesity on CACs may underlie a spectrum of progressing dysfunctional adipose tissue from VAT to greater fat accumulation or fibrosis in liver pose greater risk for subclinical atherosclerosis besides higher prevalence of 2 MetS components of DM and HTN.  

**Addendum Section 9.5.16.5 NAFLD and Body Composition**

A population-based cross-sectional study by Meng et al. [8](235) including 2238 participants aged > 40 years with mean age of 55.6 (±7.8) years from invited 9078 Jidong (China) residents into analysis and examining relationship between EAT-volume, which is CT-measurement of EAT (Epicardial Adipose Tissue) size as a visceral fat without a separating fascia interconnectedly adheres with coronary artery walls and surface of myocardium with common capillaries allowing direct interactions but under physiologic conditions it has metabolic, thermogenic brown tissue and mechanically cardioprotective characteristics, NAFLD, cardiovascular risk factors and subclinical atherosclerosis, reported across increasing quartiles of EAT-volume significantly associated with older age, higher male-gender ratio, larger hip and waist circumference, higher blood-pressure, FPG, TG, TC, LDLc, Creatinine and ALT levels, higher prevalence of DM, HTN, dyslipidemia and current/former smoker, and lower HDLc besides lower ideal cardiovascular health metrics and higher risk of CACs > 0, moreover, EAT-volume increase per 10 cm^3^ significantly associated with having CACs > 0 (OR:1.26, 95% CI:1.225, 1.295) in adjusted model-4 (of model-1: without adjustment + model-2: age, gender + model-3: smoking status, HTN, DM + model-4: BMI), and NAFLD vs no-NAFLD had significantly higher rate of elevated EAT-volume ≥ 127.46 cm^3^ (OR:1.407, 95% CI:1.117, 1.773) in multivariate logistic regression analysis of adjusted model (model-3 + level of education, BMI, hip-circumference, abdomen-circumference, TG, LDL, HDL, ALT and physical activity); in overall of these results, NAFLD significantly associated with progressed EAT-volume independently from MetS and Obesity, that EAT could mediate the contribution of NAFLD to subclinical atherosclerosis and CAD via sourcing proinflammatory activities-adipocytokines and free fatty acids, in which aggravating target organ damage by its intricate ectopic deposition to underlying myocardium and coronary arteries.  

**Addendum Section 9.5.16.6 Indexed Fatty-Liver Disease**

A retrospective cohort study by Sinn et al. [9](236) including 4731 participants with mean 52.2 years of age and 3.9 years of follow into analysis whereas excluding participants with daily alcohol intake more than gender-specific recommendations, history of cirrhosis, hepatitis (indicated by HBsAg or HCV-Antibody), CVD and cancer, and use of aspirin, warfarin or anti-thrombotic medications besides missing data to examine the longitudinal association of NAFLD and its severity indexed by NAFLD fibrosis score (NFS ≥ −1.455), which was defined by the formula −1.675 + 0.037*age (in years) + 0.094*BMI (kg/m^2^) + 1.13*impaired fasting glucose/diabetes (yes = 1 or no = 0) + 0.99*AST/ALT ratio – 0.013*Platelet Count (*10^9^/L) – 0.66*albumin (g/dL), with progression of CACs, reported in fully-adjusted model-4 (for model-1: age, gender + model-2: smoking status, alcohol intake status + model-3: BMI, SBP, TC, HDL-c, Ln[TG], DM, use of anti-hypertensive and lipid lowering medications, HbA1c and eGFR + model-4: time-dependent BMI, anti-hypertensive medications and lipid-lowering drugs) participants with NAFLD vs without NAFLD significantly associated with greater annual progression rate in Ln[CACs + 1] regardless of baseline CAC = 0 or CAC > 0 scores (Rate-ratio: 1.03, 95% CI:1.00 to 1.05), and greater average difference of annual progression in Agatston units on overall baseline (4.9, CI: 2.8 to 7.0), CACs = 0 baseline (0.4, CI:0.0 to 0.7) and CACs > 0 baseline (5.7, CI:2.1 to 9.3); moreover, again in adjusted model-4 across NAFLD severity dichotomy by NFS ≥ −1.455 compared to reference no NAFLD category significantly associated with greater rates of annual CAC progression in either Ln[CACs + 1] (rate-ratios: 1.02 and 1.06) and average difference of annual Agatston unit (2.1 and 10.8), furthermore, in subgroup analysis defined by conventional risk factors association between NAFLD and CACs progression had no significant interaction exceptionally only among elevated BP subgroup stratification (SBP ≥ 130 mm Hg and DBP ≥ 85 mm Hg or self-reported use of antihypertensive medication) patients without elevated BP had significantly greater ratio of annual progression rate for NAFLD (+) vs NAFLD (−); to sum-up, as the authors discussed, these findings suggest NAFLD longitudinally significantly associated with CACs progression independent of MetS and conventional risk factors, in which NAFLD associated with major mechanisms of atherosclerosis, and again underlying NAFLD is not the epiphenomenon of MetS severity or Obesity but hinting an early manifestation in liver due to common pathophysiological mechanisms with MetS (IR) and direct involvement in the progression of atherosclerosis as an independent risk factor.

A follow-up study by Chang et al. [10](237) including 105,328 participants of healthy young and middle aged of mean 40.8 (7.8) years with median follow-up of 3.0 years, excluding participants with missing data, history of malignancy, known liver disease or current use of medication for liver disease, positive serology for HBV or HCV, history of cirrhosis or findings on US, and use of medications causing hepatic steatosis within a year, to examine relations of fatty liver disease (FLD) categories of EAC (Excessive alcohol consumption in the absence of FLD), AFLD (alcoholic fatty liver disease, FLD in the presence of EAC) and NAFLD (non-alcoholic fatty liver disease, FLD in the absence of EAC) with coronary artery calcification at initial examination and progression during follow-up (estimated from linear mixed models with random intercepts and and random slopes) besides potential confounding roles of obesity (by BMI ≥ 25 kg/m^2^), severity of hepatic steatosis determined by ultrasonographic criteria, which are increased fine echoes in the liver parenchyma compared to kidney or spleen, deep beam attenuation and bright vessel walls, FLD severity by the fibrosis-4 (FIB-4) index, which is defined by the formula FIB-4 = age (in years) * AST (U/L) / (Platelet count (*10^9^/L) * √ALT (U/L)), and FLD severity by the aspartate transaminase-to-platelet ratio index (APRI), which is defined by the formula APRI = 100 * (AST/upper-limit of normal)/Platelet Count (*10^9^/L); reported with CACs > 0 at baseline compare to reference category (No-EAC and No-FLD) groups of EAC + FLD- (OR:1.25), AFLD (OR:1.20) and NAFLD (OR:1.10) significantly associated in fully-adjusted model-3 (of model-1: age and gender + model-2: center, year of screening exam, BMI, smoking status, Physical activity of whether Health enhancing activity-HEPA or not, education level, total calorie intake, FH of CVD, DM, HTN, LDL-c and use of medication for dyslipidemia + model-3: hs-CRP and HOMA-IR), although these associations had non-significant interaction trend for obesity status (p = 0.088), slightly stronger association for non-obese (BMI < 25 kg/m^2^ vs BMI ≥ 25 kg/m^2^) was observed at which among BMI ≥ 25 kg/m^2^ only AFLD remained significant for baseline any CACs > 0 in both adjusted model-2 (OR:1.14) and model-3 (OR:1.13), nevertheless, in multivariable adjusted model-3 compared to reference category with annual rate of CAC progression (in Ln[CACs + 1]) significantly associated with other 3 FLD categories of EAC (CACs-ratio: 1.0297), NAFLD (CACs-ratio: 1.0390) and AFLD (CACs-ratio: 1.0688) and these associations remained significant and similar regardless of obesity, but in subgroup analysis of age significantly interacted and the associations of FLD-categories with baseline any CACs strengthened for younger participants < 40 years (p < 0.001) especially for NAFLD group otherwise nonsignificant for patients with ≥40 years whereas gender, smoking status, physical activity (HEPA or not), HOMA-IR and hs-CRP had no significant interaction; furthermore, across FLD severity categories by noninvasive marker of fibrosis FIB-4 on low (<1.30) to intermediate/high categories (1.30 to <2.67 and ≥2.67 respectively) strengthened the associations of having higher baseline prevalence of CACs > 0 for both NAFLD (OR-low:1.09, OR-intermediate/high:1.14,) and AFLD (OR:1.17, OR:1.37, respectively) compare to reference group (no EAC and no FLD) in adjusted model-3, similarly across ultrasound assessed steatosis categories of mild and moderate/severe AFLD patients again more strongly associated with baseline presence of CACs > 0 compared to reference category for NAFLD (OR-mild: 1.09, OR-moderate/severe: 1.12) and exceptionally weaker for AFLD (OR: 1.20, OR:1.18, respectively), however, across APRI categories FLD had similar strength of associations with CACs > 0, moreover, in sensitivity analysis for the presence of any baseline CACs as either CACs 1–100 and CACs > 100 in multinomial regression model or Ln[CACs + 1] in Tobit regression model among FLD categories compared to reference category EAC had strongest associations than followed by AFLD or NAFLD, respectively; in summary, remaining associations of FIB-4 or hepatic steatosis severity with any CACs > 0 presence after adjustment for conventional risk factors besides systemic inflammatory marker hs-CRP and insulin-resistance index (HOMA-IR) and strengthening associations of FLD categories (especially NAFLD) with CACs in younger age category regardless of aging is a conventional CVD risk factor, may suggests inadequacy of CVD-risk factors to explain the association of FLD with subclinical atherosclerosis, besides, AFLD likewise NAFLD as a metabolic liver disease is an independent risk factor for the progression of subclinical atherosclerosis (CACs) regardless of conventional CVD risk factors with slightly stronger association than NAFLD, in which for the relation of FLD and CACs authors discussed the mechanisms as hepatic steatosis leading to dysfunctional secretory patterns of hepatokines, proatherogenic factors and proinflammatory cytokines towards interplay between unfavorable adipokine profiles, oxidative stress, low-grade inflammation, abnormal lipoprotein metabolism and IR (Systemic and hepatic), that FLD is required to be screened and treated to prevent ASCVD.

A prospective single-center study by Niikura et al. [11](238) including 101 biopsy-indicated NAFLD patients of 41 NAFLD-only and 60 NASH patients with mean age of 52.7 years into initial cross-sectional analysis, reported NASH patients vs. NAFLD-only patients in histological examination significantly associated with greater number lobular inflammation foci per 200*Field, greater number of ballooning hepatocytes, greater NAFLD-activity score (NAS) and more severe fibrosis stage (mostly stage 2 and 3) whereas two patient groups remained similar for BMI, AST, ALT, CRP, FPG, Fasting-insulin, HbA1c, DM, HTN, dyslipidemia and steatosis grade yet NASH patients were more prevalently male and with older age; CCTA measured CAS (Coronary Artery Stenosis by SCCT grading scale for degree of luminal stenosis), CACs (AU) and non-calcified plaque (with any of the signs of positive-remodeling, CT-attenuation < 30 HU, napkin-ring sign or spotty calcium) significantly associated with greater hepatocyte ballooning grade (0 to 2 as none, few and many) and increased fibrosis stage (o to 4 as none, perisinusoidal or periportal, perisinusoidal-portal/periportal, bridging fibrosis and cirrhosis) but not with steatosis grade (1–3 as 5–33%, 33–66% and >66%) and lobular inflammation grade (0–3 as none, <2 foci, 2–4 foci and >4 foci), moreover, in multiple regression analysis of potential risk factors of coronary artery stenosis (age, gender, BMI, smoking score, ALT, GGT, prevalence of renal dysfunction, DM, HTN, Dyslipidemia and NASH, and fibrosis stage) patients with CAS vs absent-CAS significantly associated with older age, higher smoking-score, fibrosis stage and greater prevalence for NASH and dyslipidemia, furthermore patients with CACs > 0 vs CACs = 0 had significantly older age and higher prevalence for DM and dyslipidemia but not NASH and HTN prevalence and fibrosis stage yet all significant in univariate, in addition patients with non-calcified plaque presence vs absent had significantly higher prevalence of dyslipidemia and NASH but not older age, fibrosis stage and DM and HTN prevalence yet all significant in univariate; in-summary of these findings patients with NASH compare to NAFLD-only patients had greater risk of unexpected CVD-events, worser atherosclerotic lesions, higher unstable non-calcified plaque burden and more intense hepatic derangements of ballooning and fibrosis, in which, as authors discussed, NASH exacerbate atherogenicity of lipoprotein subpopulation with lower microsomal expression of TG transfer protein causing excess TG in hepatocytes to increased levels of TG-rich VLDL-1 and sdLDL in serum, along with insulin resistance, oxidative stress and chronic inflammation.

A cross-sectional study by Song et al. [12](239) including 665 subjects with fatty liver disease indicated by ultrasound examination aged mean of 51.5 ± 9.3 years predominantly male (73.5%) into analysis to examine the associations of noninvasive serum fibrosis markers of NFS, FIB-4, APRI and Forn’s index, which is defined by the formula Forn’s Index = 7.811–3.131*Ln[Platelet Count, 10^9^/L] + 0.781*Ln[GGT, IU/L] + 3.467*Ln[age, years]-0.014*Cholesterol (mg/dL), and noninvasive serum steatosis markers of HSI (Hepatic Steatosis Index), which is defined by the formula HIS = 8*ALT/AST + BMI+(2, if T2 DM)+(2, if female), and FLI (Fatty Liver Index), which is defined by the formula FLI = e^y^/(1 + e^y^)*100 where y = 0.953*Ln[TG, mg/dL] + 0.139*BMI(kg/m^2^) + 0.718*Ln[GGT, U/L] + 0.053*WC(waist circumference, cm)-15.745, with CACs, reported across increasing CACs categories of 0, 0–100 and >100 had significant trend with older age, higher prevalence of DM, HTN, Lipid-lowering medication use and MetS, and increased levels of SBP, FPG, Creatinine, NAFLD-fibrosis score, FIB-4 score, Forn’s Index and WC, in addition, CACs significantly and positively correlated with non-invasive fibrosis markers of NFS (r = 0.157), FIB-4 score (r = 0.152) and Forn’s index (r = 0.120), moreover, in multinomial logistic regression model (of conventional risk factors and noninvasive markers of steatosis and fibrosis) compared to reference category of CACs = 0 participants both CACs 0–100 and CACs > 100 significantly associated with having age > 55 years (OR:2.069, OR:5.784), hypertension (OR:1.874, OR:1.958), diabetes (OR:2.134, OR:2.660), NAFLD-fibrosis score (>−2.745; OR:1.675, OR:7.863), FIB-4 score (>0.85; OR:1.788, OR:5.475) and Forn’s Index (>3.8); OR:1.711, OR:3.635, respectively, while Bilirubin > 1.0 mg/dL (OR = 0.599) and TG ≥ 150 mg/dL (OR:1.653) significantly associated for CACs 0–100 vs CACs = 0 and BMI ≥ 25 kg/m^2^ (OR:1.884), eGFR < 90 mL/min/1.73 m2 (OR:2.699), Fatty liver index (OR:1.019) and APRI (>0.219; OR:2.843) had significant association for CACs > 100 vs CACs = 0, furthermore, in multivariate binary logistic regression analysis, which was derived from significant or marginally significant conventional risk factors for CACs > 100 by univariate analysis, for CACs > 100 remained significant with Age > 55 years (OR:4.809), BMI ≥ 25 kg/m^2^ (OR:2.072) and eGFR < 90 mL/min/1.73 m2 (OR:2.149), and in multi-variate analysis adjusted by these 3 risk factors (besides gender) for the associations of noninvasive fibrosis and steatosis markers to CACs > 100 NFS (OR:3.91), FIB-4 score (OR:2.573) and APRI (OR:2.151) significantly associated whereas Forn’s index, Hepatic steatosis index and Fatty liver index had no significant association, in addition, re-formulation of noninvasive fibrosis markers based on adjusted factors of multi-variable logistic regression model improved AUROC model prediction of CACs > 100 for NFS (from 0.689 to 0.797), FIB-4 score (from 0.683 to 0.785) and APRI index (from 0.595 to 0.782) but with slightly decreased sensitivity in return of distinctly increased specificity, moreover, age of ≥55 years had significant interaction with the associations of CACs > 100 for noninvasive fibrosis markers of NFS (OR:7.929), FIB-4 score (OR:4.645) and APRI-index (OR:2.794) whereas for age < 55 years no significant association of CACs > 100 with any of the non-invasive fibrosis scores was observed; and in brief non-invasive fibrosis markers could independently predicted advanced CACs > 100, which is associated with CVE, especially for older patients aged ≥ 55 years.

A retrospective cross-sectional study by Hsiao et al. [13](240) including 817 subjects aged mean of 54.2 ± 10.0 years and examining relationship between abdominal ultrasound assessed severity of NAFLD (depending on the liver parenchyma echogenicity relative to kidney/spleen, as normal, mild, moderate and severe) and the subclinical atherosclerosis measured by CCTA, which are defined by CACs ≥ 100, CACs ≥ 400, CAC-RADS ≥ 3 (50–69% moderate occlusion in any of the triple coronary arteries) and vulnerable plaques (with any of the 4 features as positive remodeling, low attenuation plaques, napkin ring sign and spotty calcium), reported across 4 categories of NAFLD (normal to severe) significantly associated by one-way ANOVA analysis in positive general trends for the rate of male gender, BMI, prevalence of DM and HTN, TG and HbA1c levels, the rate of having Framingham Risk score of FRS ≥ 6% vs <6% and the risk of having subclinical atherosclerosis in measurements of CACs ≥ 100, CACs ≥ 400, CAD-RADS ≥ 3 and vulnerable plaques and in inverse trend for TC and HDL-c levels yet for LDL-c, Body-fat percentage, eGFR, smoking habits and pack-years of smoking remained similar, in addition, by Bonferroni post-hoc tests participants with mild vs normal NAFLD had no significant association with any of the SCVD measurements but participants with mild NAFLD were slightly younger than normal participants; besides, moderate vs normal or moderate vs mild had significantly higher odds of CACs ≥ 100 and participants with severe vs moderate NAFLD significantly associated only with CACs ≥ 400 among SCVD measurements but again participants in severe NAFLD were slightly younger than in moderate NAFLD, moreover, severe vs mild NAFLD participants had significant association in any of 4 SCVD measurements and severe vs normal NAFLD participants had significant associations for any of the 3 SCVD measurements excepting CAD-RADS ≥ 3, furthermore, in binary logistic regression analysis compare to reference category (consisting of normal to moderate NAFLD participants) patients with severe NAFLD had significantly higher OR for CACs ≥ 100 (2.946), CACs ≥ 400 (OR:4.402), CAD-RADS ≥ 3 (OR:4.091) and presence of vulnerable plaques (OR:2.906) after adjustment for FRS (Framingham risk score) and body-fat percentage; as a summary, these results may suggest severe degree NAFLD is an independent risk factor for SCVD (by CACs ≥ 100, CACs ≥ 400, CAD-RADS ≥ 3 and vulnerable plaques) regardless of FRS and Body-fat percentage, and significant linear trend of the NAFLD severity with SCVD, metabolic risk factors and FRS.

A follow-up study by Ichikawa et al. [14](241) including 529 patients (of are 143 NAFLD, which was indicated by non-contrast abdominal CT-scan, and 386 non-NAFLD patients aged mean 60 ± 12 and 67 ± 12, respectively), who had no CVD event, predefined liver disease and cancer comorbidity histories, no use of oral corticosteroids or amiodarone, daily alcohol intake < 20 g but had referral due to suspected CAD, aged mean of 65 years with a median follow-up period (after CT examination) of 4.4 years into analysis to examine the predictive values of CACs, FRS and NAFLD in the endpoint of CVE (Cardiovascular events), revealed patients with NAFLD vs without NAFLD at baseline significantly associated with lower CACs categories, younger age, greater rates of very-high-risk patients by ESC, higher levels of BMI, visceral adipose tissue area, eGFR, AST, ALT and TG, higher prevalence of dyslipidemia and obesity, higher rates of using oral antihyperglycemic drugs, metformin and DPP-4 inhibitors, and lower levels of creatinine and HDL-c, furthermore, by Kaplan-Meier curves for non-zero CACs categories of 1–99 (2.25% vs. 0.33%) and ≥100 (6.40% vs. 1.78%) patients with NAFLD vs no-NAFLD had significantly higher incidence of CVE yet for patients with CACs = 0 remained similar independent of NAFLD status for the incidence of CVE (0.00% vs. 0.30%), nonetheless, in overall population without stratification by CACs categories patients with NAFLD compared to without NAFLD had significantly higher incidence of CVE, moreover, multivariate COX-regression analysis indicated NAFLD (HR:5.43), CACs (Log[CACs + 1], HR:1.56) and FRS (HR:1.23) significantly predicted CVEs, besides, for predicting CVE adding NAFLD status to model of CACs and FRS improved model estimate by global χ2 score from 27.0 to 49.7, ROC analysis from 0.71 to 0.80, and also the net reclassification index; and these findings may underlie both baseline NAFLD and CACs independently associates with CVEs and combined use might incrementally improves the predictivity of the novel risk marker CACs in revealing higher CVE risk especially for the patients at intermediate risk by FRS.  

**Addendum Section 9.6:**

• On the page-19 at the paragraph 4 and 5 elaborating the association between FH and CVD-events by further adjustment and revealing the adjusted covariates for the multivariable analysis of COX proportional hazard models between mortality rates and CACs were deemed contributory to cultivate the further discussions across reviewed data within the study; and then in accordance the relavant clauses were edited respectively as follows:
“(…) observed family history of CHD associated with higher odds of both CVD (HR:1.73) and CHD (HR:1.60) events on adjustment for age and gender, and further adjustment for FRS and baseline use of statin and aspirin only CVD-events (HR:1.72) remained significant.”
“(…) and in multi-variable analysis of COX proportional hazard models (adjusted for HTN, DM, Hyperlipidemia, smoking status, gender and race) CAC > 100 compared to CAC = 0 had higher risk for all-cause mortality in 2.2-fold, CVD-specific mortality in 4.3-fold, and CHD-specific mortality in 10.4-fold.”
• On the page-20 at the paragraph-1 testing the possible interaction by the source of FH in CACs progression after adjustment for baseline CACs was deemed contributory for further discussions, and in accordance the relevant paragraph section was edited as follows:
“(…) and regarding the source of FH sibling FH of premature CHD had significant median CACs progression (17.0 in CAC-volume score) but either parenteral FH or combined sibling and parenteral FH didn’t have significant association, nevertheless after further adjustment for logarithm of baseline CACs no significant interaction by the source of FH was observed in CACs progression.”  

**Corrigendum Section 9.6:**

• On the page-19 at the paragraph-7, event rate data for CVD were realized lacking labeling for categories of participants as late onset FH of CHD and without FH of CHD. The relevant paragraph section was edited as follows:
“(…) reported participants with premature FH of CVD as 7.24 (per 100 person-years), late onset family history as 6.56 (per 100 person-years) and no family history as 5.87 (per 100 person-years), (…)”
• On the same paragraph-7 (p.19), in definition of model adjustment by its covariates a conjunction error was realized, that instead of “or” “and” was used. So correct definition will be as follows:
“(…) yet premature CHD in siblings couldn‘t have significant association after adjustment beyond demographics for conventional risk factors or FRS (…)”
• On the page-20 at the 4th paragraph, an absent exponential sign in reporting genome-wide significance was noticed, and the relavant clause was edited as follows:
“(…) reported GWAS of discovery set in additive genetic model of logistic regression model (adjusted for age, gender, HTN and DM) identified only one SNP (rs10757272) on chromosome 9p21.3 within intronic region of CDKN2B-AS1 (Cyclin-dependent kinase inhibitor 2B anti-sense RNA gene) passing Bonferroni correction at p = 7.55E–08 (OR:3.24, 95 %CI: 2.11–4.97) and this SNP remained significant in replication set by PCR-assay (p = 0.036).”  

**Corrigendum Section 9.7:**

• On the page-20 at the paragraph-5 typing errors noting “CVD” instead of “CHD” and a duplicated phrase were realized, and edited with the underlying inverse association between CACs and event-free survival rates in the relevant clause as follows:
“(…) who stratified study population into age groups (defined by 45-54, 55-64, 65-74 and 75-84) and CAC groups (0, 1–100 and > 100), observed across increasing age groups as 75–84 years old age group compared to 45–54 years old age group, proportion of non-zero CAC score, CAC > 100 and CHD-event risk increased, but at each age-group increase in CAC from 0 to >100 resulted in similar increase in CHD-event rate and similar reciprocal decrease in CHD-event free survival by Kaplan-Meier curves as such age-related increase in CHD-event risk (in any levels of sequentially adjusted models of unadjusted + gender, ethnicity and MESA-site + HTN, DM, LDL-c, HDL-c, TG, smoking, FH of CHD, BMI, mean-HR, anti-hypertensives, lipid lowering medications and education level) attenuates significantly after further adjustment for CAC-score with similar survival rates (…)”  

**Addendum Section 9.7:**

• On the page-20 at the paragraph-6 delineating the associations of high CACs > 100 vs CACs = 0 with CVD, CHD and all-cause mortality rates as independent of multivariable adjustment were deemed contributory and the relevant clause was edited as follows:
“(…) CAC > 100 compared to CAC = 0 significantly associated with higher prevalence of each of the 5 traditional risk factors as hypertension, hyperlipidemia, current smoking family history of premature-CHD and diabetes, higher cumulative number of presenting risk factors, and then higher risk of CVD, CHD and all-cause mortalities regardless of multivariable adjustments for these 5 traditional risk factors, age and gender.”  

**Addendum Section 9.8:**

• On the page-20 at the paragraph-8 further detailing the associations between long-term exposure to neighborhood environment components and incidence of T2DM with it’s further adjustment for neighborhood socioeconomic status (SES) was considered contributory for the discussion across this section, and the relavant clause was edited as follows:
“(…) however, summary measures for social environment had largely unassociated, and after further adjustment of the multivariable model with neighborhood SES (Socioeconomic Status, which was determined by the principal component analysis of 21 census variables estimated by American Community Survey) the association of IQR increase of summary healthy food environment attenuated yet IQR increase of summary physical activity measures remained significant.”
• On the page-20 at the paragraph-9 for two cited studies covariates of model adjustments weren’’t revealed. The relevant paragraph section was edited to include these covariates as follows:
“(…) Brenner et al. [79] revealed that one standard deviation increases in neighborhood SES associated with a reduction in the probability of current alcohol use for both genders (all models adjusted for age, ethnicity, marital status, income, education, job status and study site; for weekly alcohol use in gender stratified negative binomial regression models) while Brenner et al. [78] discerned that, living in a neighborhood in the highest disadvantaged tertile associated with a lower probability of current alcohol use (all models adjusted for age, ethnicity, marital status, income, education, job status, study site and significant interactions between time in invariant predictors and time since baseline in hybrid effect models). (…)”.
• On the page-21 at the paragraph-2 the intrinsic limitation for the econometric fixed effect model, time-varying nature of covariates in model adjustments, the sensitivity analysis in the econometric fixed effect model and identifications of the ArcGIS determined stores and facilities weren’t shown to remain concise. The relevant paragraph sections were edited to include these elucidations respectively as follows:
“(…) furthermore, in econometric fixed effects model allowing testing simultaneous mean changes within person in exposures and outcomes per 1-SD increase, which has limited efficiency when within-person variability in exposures or outcomes is low, only density of healthy food stores among presented 5 neighborhood characteristics significantly associated with within-person changes in CACs in models adjusted with time-varying covariates as progressively more proximal to outcome more like a causal pathway at −19.99 in model-1 (age, marital status, income, working status and CT-scanner type), −18.99 in model-2 (model-1 + moderate/vigorous physical activity and cigarette smoking), −19.41 in model-3 (model-2 + Center for Epidemiological Studies Depression Scale score) and − 17.60 in model-4 (model-3 + TC/HDL-c, BMI, HTN, DM and lipid-lowering medication use), and in sensitivity analysis further adjustment for either neighborhood socioeconomic status or moving indicator didn’t materially changed this association.”.
“(…) through comparing geographic information systems (ArcGIS) determined density of healthy food stores (Fruits and vegetable markets and supermarkets ≥ $2 M annual sales or ≥ 25 employees) and recreational facilities (indicated by 114 standard industrial classification codes) within 1 mile of participants‘ home (according to National Establishment Time Series database and Nielsen TDLinx Service Supermarket Retail Category Database) by normal kernel estimation (units per square mile) and survey-based neighborhood scales per participants of rating (1to5) for availability healthy food, walking environment, safety and social cohesion (combined into a summary social environment index due to high correlation between safety and social cohesion, Cronbach α = 0.77) within 1 mile of participants‘ home with CACs imaging (…)”.
• On the page-21 at the paragraph-4 the covariates of the adjusted models weren’t revealed. This paragraph was edited to extend these covariates as follows:
“Dragano et al. [100] with 11,263 participants in bivariate models by multivariate hierarchical regression models (to differentiate the contextual effects as geographic or social units and individual level effects in the two-level data structure) revealed male patients living in an area close to a major road ≤ 100 m as a measurement of traffic-exposure and high neighborhood unemployment rate had 2.12 times higher risk of CAC ≥ 75th percentile compared to reference group of men living in area with low-unemployment and distant to a major road > 100 m (adjusted for age and individual education level), men with ≤13 years educational attainment and living in high traffic exposure (≤100 m) versus men with ≥14 years education and living in low traffic exposure (≥100 m) 1.85 odds of CAC ≥ 75th% (adjusted for age and neighborhood unemployment rate); moreover, among women with low traffic-exposure high neighborhood-unemployment rate versus low rate with 1.31 odds of having CAC ≥ 75th% (adjusted for age and individual education level) and low-individual income versus high individual income with 1.36 odds of CAC ≥ 75th% (adjusted for age and neighborhood unemployment rate) were presented.”  

**Corrigendum Section 9.8:**

• On the page-21 at the paragraph-3, on the association between personality trait of anxiety and CACs progression in univariate linear regression a typing error was realized, in which “anxiety” was confused with “personality trait of anger” and author apologizes for any inconvenience. The relavant paragraph section was edited with additionally extending the null associations of personality traits with either baseline or incident CACs > 0 in sequentially adjusted models as follows:
“(…) reported in any sequentially adjusted models (for age, gender, ethnicity + socioeconomic factors + personality trait covariates+ and CVD risk factors) CACs > 0 at baseline had no significant association with any of the trait likewise incident CACs > 0 had also no significant association with any of the trait per unit increase even regardless of adjustment, moreover, difference in CACs between initial examination and final outcome or ΔCACs had no significant association with any of the personality trait in any adjusted model, nonetheless, in univariate linear regression only anxiety had significant association with ΔCACs.”
• On the page-21 at the paragraph-6, comparisons of 2 nominal variables were noticed unintentionally labeled categorically inverse to the analysis. Author apologizes for any inconvenience. The relevant paragraph section was edited as follows:
“(…) reported FSM compared to USM significantly associated with lower serum cholesterol level (207.1 mg/dL vs 225.3 mg/dL), lower risk of cholesterol ≥ 200 mg/dl (59.8% vs 74.6%), lower serum LDL-c (129.7 mg/dL vs 142.2 mg/dL), lower probability of LDL-c ≥ 130 mg/dl (46.7% vs 60.5%), lower fasting plasma glucose level (99.1 mg/dL vs 104.1 mg/dL), lower prevalence of impaired glucose tolerance (11.2% vs 26.3%), lower probability of pre-diabetes mellitus status (6.5% vs 19.3%) and lower exposure to air pollutants of SO_2_, NO, NO_2_, NO_x_, CO and PM10, and higher O_3_ but similar to outdoor-urban environment in univariate models, and in multivariable adjusted models (of age, gender, fasting sugar, SBP, BMI, LDL-c, smoking and alcohol) self-reported better physical health domain in WHO-Health quality assessment questionnaire, higher working hours per week, and lower amount of coffee consumption in contrast higher consumption of tea and alcohol in univariate models, moreover, the study measured in forest group compared to urban group significantly lower ABI, lower mean c-IMT in ICA, and lower maximum and mean of IMT in multivariable adjusted models.”
• On the page-21 at the paragraph-7, for the associations between absolute CACs and air pollutant O_3_ (per 15 µg/m^3^) and distance to major road per 50% decrease the results were noticed typed incorrectly that the CACs change for O_3_ was confused with the upper limit of CI for the association between NO_2_ and CACs and similarly the percentage of change in CACs for major road per 50% decrease was confused with the result of O_3_. Author apologizes for any inconveniences. The relevant paragraph section was edited with further delineations of the model adjustments, the prediction of severe CACs > 400 by exposure variables and trend analysis across quartile scores as follows:
“(…) reported in fully-adjusted single pollutant models (of age, gender, BMI, smoking, pack-years, cigarettes per day, alcohol, education, exercise, urbanization, region, distance to hospital and Beijing residence) long term exposure to air pollutants and traffic at each increasing level for PM_2.5_ per 30 µg/m^3^, NO_2_ per 20 µg/m^3^, O_3_ per 15 µg/m^3^ and distance to major road per 50% decrease significantly associated with both higher absolute CAC scores (log[CACs + 1]) as 29.6%, 33.2%, 10.8% and 3.2% respectively, and higher rates of presence of any non-zero CAC with OR:1.28, OR:1.27, OR:1.12 and OR:1.04 respectively, in addition, both PM_2.5_ and NO_2_ significantly associated with severe CACs > 400 (OR:1.59 and OR:1.60, respectively) in fully-adjusted single pollutant models (remaining significant even after further adjustment for either antihypertensive and statin use or conventional lipid panel and hs-CRP) and had significant trend of % change in CACs across quartile scores; moreover, they demonstrated male gender, older age > 60 years, and diabetes history (only for PM_2.5_ per 30 µg/m^3^) pronounced the associations between CAC score and exposures to PM2.5 and NO_2_.”  

**Erratum Section 9.8:**

• On the page-21 at the paragraph-5 the link for the cited reference study was noticed not working due to an absent dot in the string of doi number. Noted doi number of “https://doi.org/10.1161/JAHA.115002965” was changed by “https://doi.org/10.1161/JAHA.115.002965”  

**Addendum Section 9.9:**

• On the page-22 at the 1’st paragraph, for analyses as in sequentially adjusted multivariable models revealing adjusted covariates were deemed contributory for further discussions, and in accordance the relavant paragraph section was edited as follows:
“(…) reported incidence rates for non-cardiovascular diseases of cancer, CKD, pneumonia, COPD, DVT/PE, dementia and hip fracture had significant linear trend across increasing CAC stratums (CAC = 0, CAC = 1–400 and CAC > 400), and in the multivariable adjusted model-5 (of Model-1: unadjusted + Model-2: age, gender, race, health insurance status and socioeconomic status by educational attainment of completed high school and income level + Model-3: BMI, physical activity, diet, smoking status, pack-years + Model-4: total number of medication use + Model-5: traditional cardiovascular risk factors of SBP, DBP, TC, HDL-c, DM, antihypertensive use, lipid-lowering therapy and aspirin use) doubling of Log-transformed CACs (log_2_(CACs + 1)) significantly associated with higher HRs for cancer (1.04), CKD (1.07), pneumonia (1.07), COPD (1.10), dementia (1.06), hip-fracture (1.10) and any non-CVD (1.06), and in age sensitive interval analysis (of model-6: model-5 + ≥65 years vs <65 years) despite non-significant interaction because of limited power in < 65 years doubling of CAC only among ≥ 65 years of age group remained significant with increased hazards of cancer (1.04), CKD (1.07), pneumonia (1.08), COPD (1.09) and any non-CVD (1.06) with attenuated associations for dementia and hip-fracture, moreover, CAC > 400 vs CAC = 0 had significantly increased hazards of cancer (1.53), CKD (1.70), pneumonia (1.97), COPD (2.71), hip-fracture (4.29) and any non-CVD (1.80) in the model-5 adjustment, and a CAC = 0 compared to any CAC > 0 had significantly lower incident cancer (0.76), CKD (0.77), COPD (0.61), hip-fracture (0.31) and any non-CVD (0.75) in the model-5 adjustment.”
• On the page-22 at the paragraph-2 age adjustment for dementia incidence and p for trend weren’t shown. The relevant paragraph section was edited as follows:
“(…) but exceptionally among white women increasing CAC-strata significantly associated with increased risk of dementia culminated at CAC > 400 vs CACs = 0 (age-adjusted incidence of 102 vs 31 per 1000 person-years, p-for trend: 0.044).”
• On the page-22 at the 4th paragraph, at each non-zero CACs categories compared to CAC = 0 hazard ratios of incident diseases weren’t shown, and in accordance the relavant clauses were edited as follows:
“(…) moreover, compared to reference CAC = 0 at each subsequent CAC-score categories defined by score points of 1, 100 and 300, associated significantly with higher CVD (adjusted HR:1.44, HR:2.26 and HR:3.68, respectively), CHD (adjusted HR:1.49, HR:2.74 and HR:4.80, respectively) and non-CVD mortality (adjusted HR:1.19, HR:1.39 and HR:1.85, respectively) risks in unadjusted and multivariable adjusted models (of age, gender, HTN, Hyperlipidemia, smoking, diabetes and FH of CHD) except cancer mortality rate remained similar for a CAC-score < 300 in adjusted models, nonetheless a CAC ≥ 300 had significantly higher cancer mortality risk than the zero-CAC score in multivariable adjusted model (HR:1.30).”  

**Corrigendum Section 10.1:**

• On page-23 at the paragraph-2, several unwitting slight errors in derivation and report of the NNT analysis, that wouldn’t substantially differ e.g., for NNT-5 as 27 vs 28, were noticed. Author apologizes for any inconvenience and the relevant clause was edited as follows:
“(…) furthermore, for participants with CACs > 100 13.65% CVD event rate at 5.8 years occurred through Kaplan-Meier statistics with estimated NNT-5 years (with Rosuvastatin treatment, HR:0.56) for CVD as 19 or adjusted NNT-5 years (with moderate statin 31% CVD-event risk reduction) with the Altman-Anderson method as 27 or projected NNT-10 years (with moderate statin 31% CVD-event risk reduction) as 15.”  

**Corrigendum Section 10.2:**

• On page-25 at the paragraph-2, aspirin effect estimates in terms of RRI of bleeding and RRR of ASCVD to evaluate absolute effects of aspirin on 10-year ASCVD risk prevention in return of increased 10-year bleeding risk were noticed unintentionally just inversely labeled for the outcomes of ASCVD and Bleeding risks. Author apologizes for any inconvenience due to this error and the relevant clause was edited as follows:
“A cohort study by Ajufo et al. [213] with mean follow-up period of 12.2 (1.9) years involving 2191 participants aged mean 44.4 (9.1) without already existing ASCVD and baseline aspirin use recruited from the Dallas Heart Study to investigate role of CACs in guiding aspirin allocation through ASCVD (RRR of 10%) and bleeding events (RRI of 39%), (…)”  

**Addendum References:** Since the section 9.5.16 Fatty Liver Disease (FLD) was absent from the study, cited references within this section are required to be listed as follows:

(228) R. Wu, et al., Nonalcoholic Fatty Liver Disease and Coronary Artery Calcification in a Northern Chinese Population: a Cross Sectional Study, Scientific Reports, 2017, https://doi.org/10.1038/s41598-017-09851-5.

(229) S. B. Lee, et al., Association between non-alcoholic fatty liver disease and subclinical coronary atherosclerosis: An observational cohort study, Journal of Hepatology, 2018, https://doi.org/10.1016/j.jhep.2017.12.012.

(230) Y. K. Cho, et al., The impact of non-alcoholic fatty liver disease and metabolic syndrome on the progression of coronary artery calcification, Scientific Reports, 2018, https://doi.org/10.1038/s41598-018-30465-y.

(231) A. Gummesson, et al., Non-alcoholic fatty liver disease is a strong predictor of coronary artery calcification in metabolically healthy subjects: A cross-sectional, population-based study in middle-aged subjects, PloS ONE, 2018, https://doi.org/10.1371/journal.pone.0202666.

(232) M.S. Kwak, et al., Nonalcoholic fatty liver disease is associated with coronary artery calcium score in diabetes patients with higher HbA1c, Diabetology & Metabolic Syndrome, 2015, https://doi.org/10.1186/s13098-015-0025-4.

(233) J. Kim, et al., Increased risk for development of coronary artery calcification in subjects with non-alcoholic fatty liver disease and systemic inflammation, PLoS ONE, 2017, https://doi.org/10.1371/journal.pone.0180118.

(234) M. K. Lee, et al., Higher association of coronary artery calcification with non-alcoholic fatty liver disease than with abdominal obesity in middle-aged Korean men: the Kangbuk Samsung Health Study, Cardiovascular Diabetology, 2015, https://doi.org/10.1186/s12933-015-0253-9.

(235) X. Meng, et al., Epicardial adipose tissue volume is associated with non-alcoholic fatty liver disease and cardiovascular risk factors in the general population, Therapeutics and Clinical Risk Management, 2018, https://doi.org/10.2147/TCRM.S168345.

(236) D. H. Sinn, et al., Non-alcoholic fatty liver disease and progression of coronary artery calcium score: a retrospective cohort study, Hepatology, 2016, https://doi.org/10.1136/gutjnl-2016-311854.

(237) Y. Chang, et al., Alcoholic and non-alcoholic fatty liver disease and associations with coronary artery calcification: evidence from the Kangbuk Samsung Health Study, Hepatology, 2019, https://doi.org/10.1136/gutjnl-2018-317666.

(238) T. Niikura, et al., Coronary Artery Disease is More Severe in Patients with Non-Alcoholic Steatohepatitis than Fatty Liver, Diagnostics, 2020, https://doi.org/10.3390/diagnostics10030129.

(239) D. S. Song, et al., Noninvasive Serum Fibrosis Markers are Associated with Coronary Artery Calcification in Patients with Nonalcoholic Fatty Liver Disease, Gut and Liver, 2019, https://doi.org/10.5009/gnl18439.

(240) C. C. Hsiao, et al., Severe, but not mild to moderate, non-alcoholic fatty liver disease associated with increased risk of subclinical coronary atherosclerosis, BMC Cardiovascular Disorders, 2021, https://doi.org/10.1186/s12872-021-02060-z.

(241) K. Ichikawa, et al., Prognostic value of non-alcoholic fatty liver disease for predicting cardiovascular events in patients with diabetes mellitus with suspected coronary artery disease: a prospective cohort study, Cardiovascular Diabetology, 2021, https://doi.org/10.1186/s12933-020-01192-4.

(242) P. H. Joshi, et al., The 10-Year Prognostic Value of Zero and Minimal CAC, JACC: Cardiovascular Imaging (2017), https://doi.org/10.1016/j.jcmg.2017.04.016.  

**Erratum References:**

• For the reference study-2 the cited doi number as “https://doi.org/10.1093/eurheart/ehw106” changed into “https://doi.org/10.1093/eurheartj/ehw106”.
• For the reference study-7 the citation note as “P. Arthur, DeMarzo, Using impedance cardiography (…)” was edited into “Arthur P. DeMarzo MS, Using impedance cardiography (…)”
• For the reference study-18 the cited doi number as “https://doi.org/10.1161/CIRCULATIONAHA.109.192703.” changed into “https://doi.org/10.1161/JAHA.116.004894”. The citation note as “Donald M. Lloyd-Jones et al., Defining and Setting (…)” was edited into “Donald M. Lloyd-Jones, et al., Defining and Setting (…)”.
• For the reference study-19 the cited doi number as “https://doi.org/10.1161/CIRCULATIONAHA.109.192278.” changed into “https://doi.org/10.1161/CIRCULATIONAHA.109.192278”. The citation note as “Mark A. Hlatky et al., Criteria for (…)” was edited into “Mark A. Hlatky, et al., Criteria for (…)”.
• For the reference study-21 the cited doi number as “https://doi.org/10.5551/jat.RV160005” changed into “https://doi.org/10.5551/jat.RV16005”.
• For the reference study-24 the cited doi number as “https://doi.org/10.1161/CIR.0000000000000659.” changed into “https://doi.org/10.1161/CIR.0000000000000659”. The citation note as “Emelia J. Benjamin et al.,(…)” was changed into “Emelia J. Benjamin, et al.,(…)
• For the reference study-27 the cited doi number as “https://doi.org/10.1161/CIRCULATIONAHA.111.070722/_/DC1.” changed into “https://doi.org/10.1161/CIRCULATIONAHA.111.070722”. The citation note as “Mark D. Huffman et al., Cardiovascular Health Behavior (…)” was edited into “Mark D. Huffman, et al., Cardiovascular Health Behavior (…)”.
• For the reference study-41 the citation note of “(…) Cardiovascular Imaging (2017; https://doi.org/10.1016/j.jcmg.2017.05.007.)” changed into “(…) Cardiovascular Imaging (2017), https://doi.org/10.1016/j.jcmg.2017.05.007”.
• For the reference study-47 the cited doi number as “https://doi.org/10.1016/j.jcmg.2017.04.016” changed into “https://doi.org/10.1016/j.jcmg.2017.04.018”. The study with doi number of “https://doi.org/10.1016/j.jcmg.2017.04.016” was added to references as the reference study 242
• For the reference study-57 the cited doi number as “https://doi.org/10.1007/978-1-4339-7274-6_28.” changed into “https://doi.org/10.1007/978-1-61779-555-8_27”.
• For the reference study-70 the cited doi number as “https://doi.org/10.1017/S0007114518002513” changed into “https://doi.org/10.1017/S0007114518003513”.
• For the reference study-74 the citation note as “Association between hsCRP ≥ 2, Coronary Artery Calcium, and Cardiovascular Events- Implications for the JUPITER Population: Multi-Ethnic Study of Atherosclerosis (MESA) (…)” was changed into “Associations between C-reactive protein, coronary artery calcium, and cardiovascular events: implications for the JUPITER population from MESA, a population-based cohort study (…)”.
• For the reference study-89 the cited doi number as “https://doi.org/10.1177/2047487318788930.” changed into “https://doi.org/10.1177/2047487318788930”. The citation note as “Bittencourt et al., Implications (…) was edited into “Bittencourt, et al., Implications (…)”.
• For the reference study-97 the cited doi number as “https://doi.org/10.1016/j.jcmg.2014.04.004.” changed into “https://doi.org/10.1016/j.jcmg.2014.04.004”. The citation note as “Andre R. M. Paixao et al., Coronary Artery Calcification (…) was edited into “Andre R. M. Paixao, et al., Coronary Artery Calcification (…)”.
• For the reference study-101 the cited doi number as “https://doi.org/10.1161/JAHA.115002965” changed into “https://doi.org/10.1161/JAHA.115.002965”.
• For the reference study-109 the citation note as “(…) The Relationship of Coronary Artery Calcium To Coronary Heart Disease Events is Similar in Young and Elderly Participants in The Multi-Ethnic Study of Atherosclerosis: A secondary Analysis of a Prospective Population-based Cohort (…)” was edited into “(…) Association of coronary artery calcium and coronary heart disease events in young and elderly participants in the multi-ethnic study of atherosclerosis: a secondary analysis of a prospective, population-based cohort (…)”.
• For the reference study-127 the cited doi number as “https:// https://doi.org/10.1001/jamacardio.2018.4628.” changed into “https:// https://doi.org/10.1001/jamacardio.2018.4628”. The citation note as “LF-DeFina et al., Association of All-Cause and (…)” was edited into “LF-DeFina, et al., Association of All-Cause and (…)”.
• For the reference study-128 the cited doi number as “https://doi.org/10.1016/j.mayocpiqo.2020.02.005.” changed into “https://doi.org/10.1016/j.mayocpiqo.2020.02.005”. The citation note as “Rozanski et al., Associations Among (…)” was edited into “Rozanski, et al., Associations Among (…)”.
• For the reference study-151 the cited doi number as “https://doi.org/10.1016/j.amjcard.2019.08.029.” changed into “https://doi.org/10.1016/j.amjcard.2019.08.029”. The citation note as “Pedrosa at al., Relation of Thoracic Aortic and (…)” was edited into “Pedrosa, et al., Relation of Thoracic Aortic and (…)”.
• For the reference study-168 the citation note as “V. S. Nunes, The coronary artery calcium score is linked to plasma cholesterol synthesis and absorption markers: Brazilian Longitudinal Study of Adult Health, Bioscience Reports, 2020, 0.1042/BSR20201094.” was edited into “V. S. Nunes, et al., The coronary artery calcium score is linked to plasma cholesterol synthesis and absorption markers: Brazilian Longitudinal Study of Adult Health, Bioscience Reports (2020), https://doi.org/10.1042/BSR20201094”
• For the reference study-187 the citation note as “N. Ei Ei Khaing et al., Epicardial and visceral adipose tissue in relation to subclinical atherosclerosis in a Chinese population, PLOS ONE, 2018, https://doi.org/10.1371/journal.pone.0196328.” was edited into “N. Ei Ei Khaing, et al., Epicardial and visceral adipose tissue in relation to subclinical atherosclerosis in a Chinese population, PLOS ONE (2018), https://doi.org/10.1371/journal.pone.0196328”
• For the reference study-204 the cited doi number as “https:// https://doi.org/10.1002/9781119536604.” changed into “https:// https://doi.org/10.1002/9781119536604”. The citation note as “Higgins et al., Cochrane Handbook for (…)” was edited into “Higgins, et al. Cochrane Handbook for (…)”.
• For the reference study-209 the citation note as “(…) JACC: Cardiovascular Imaging (2017, 10.106/j.jcmg.2017.04.014.)” was edited into “(…) JACC: Cardiovascular Imaging (2017), https://doi.org/10.1016/j.jcmg.2017.04.014”.
• For the reference study-212 the citation note as “M. Cainzos-Achirica et al., Coronary Artery Calcium for Personalized Allocation of Aspirin in Primary Prevention of Cardiovascular Disease in 2019: The MESA Study, Circulation, 2020, https://doi.org/10.1161/CIRCULATIONAHA.119.045010.” was edited into “M. Cainzos-Achirica, et al., Coronary Artery Calcium for Personalized Allocation of Aspirin in Primary Prevention of Cardiovascular Disease in 2019: The MESA Study (Multi-Ethnic Study of Atherosclerosis), Circulation (2020), https://doi.org/10.1161/CIRCULATIONAHA.119.045010”.
• For the reference study-215 the citation note as “N. k. Kalia et al., Motivational effects of coronary artery calcium scores on statin adherence and weight loss, Coronary Artery Disease, 2015, 101097/MCA.0000000000000207.” was edited into “N. k. Kalia, et al., Motivational effects of coronary artery calcium scores on statin adherence and weight loss, Coronary Artery Disease (2015), https://doi.org/10.1097/MCA.0000000000000207”.
• For the reference study-216 the citation note as “(…) Atherosclerosis (2021, 10.106/j.atherosclerosis.2021.08.002.)” was edited into “(…) Atherosclerosis (2021), https://doi.org/10.1016/j.atherosclerosis.2021.08.002”.  

**Correction of Abstract:**

The phrase as “due to hypertension” for endothelial damage was parenthesized as one of the causal mechanisms of ongoing endothelial damage. The explanation mark wasn’t noticed. Author apologizes for any inconvenience.

“ASCVD are the leading causes of mortality and morbidity among Globe. Evaluation of patients’ comprehensive and personalized risk provides risk management strategies and preventive interventions to achieve gain for patients. Framingham Risk Score (FRS) and Systemic Coronary Risk Evaluation Score (SCORE) are two well studied risk scoring models, however, can miss some (20–35%) of future cardiovascular events. To obtain more accurate risk assessment recalibrating risk models through utilizing novel risk markers have been studied in last 3 decades and both ESC and AHA recommends assessing Family History, hs-CRP, CACS, ABI, and CIMT. Subclinical Cardiovascular Disease (SCVD) has been conceptually developed for investigating gradually progressing asymptomatic development of atherosclerosis and among these novel risk markers it has been well established by literature that CACS having highest improvement in risk assessment. This review study mainly selectively discussing studies with CACS measurement. A CACS = 0 can down-stratify risk of patients otherwise treated or treatment eligible before test and can reduce unnecessary interventions and cost, whereas CACS ≥ 100 is equivalent to statin treatment threshold of ≥7.5% risk level otherwise statin ineligible before test. Since inflammation, insulin resistance, oxidative stress, dyslipidemia and ongoing endothelial damage (e.g., due to hypertension) could lead to CAC, ASCVD linked with comorbidities. Recent cohort studies have shown a CACS 100–300 as a sign of increased cancer risk. Physical activity, dietary factors, cigarette use, alcohol consumption, metabolic health, family history of CHD, aging, exposures of neighborhood environment and non-cardiovascular comorbidities can determine CACs changes.”
